# 2-(Piperidin-4-yl)acetamides as Potent Inhibitors of Soluble Epoxide Hydrolase with Anti-Inflammatory Activity

**DOI:** 10.3390/ph14121323

**Published:** 2021-12-17

**Authors:** Juan Martín-López, Sandra Codony, Clara Bartra, Christophe Morisseau, María Isabel Loza, Coral Sanfeliu, Bruce D. Hammock, José Brea, Santiago Vázquez

**Affiliations:** 1 Laboratori de Química Farmacèutica (Unitat Associada al CSIC), Facultat de Farmàcia i Ciències de l′Alimentació, Universitat de Barcelona, Avinguda Joan XXIII 27–31, 08028 Barcelona, Spain; juanxi.martin@gmail.com (J.M.-L.); sandra.codony@ub.edu (S.C.); 2 Institute of Biomedicine (IBUB), Universitat de Barcelona, Avinguda Joan XXIII 27–31, 08028 Barcelona, Spain; 3 Institut d’Investigacions Biomèdiques de Barcelona (IIBB), CSIC and IDIBAPS, C/Roselló 161, 08036 Barcelona, Spain; clara.bartra@iibb.csic.es (C.B.); coral.sanfeliu@iibb.csic.es (C.S.); 4 Department of Entomology and Nematology, University of California Davis, One Shields Avenue, Davis, CA 95616, USA; chmorisseau@ucdavis.edu (C.M.); bdhammock@ucdavis.edu (B.D.H.); 5 Comprehensive Cancer Center, University of California Davis, One Shields Avenue, Davis, CA 95616, USA; 6 Drug Screening Platform/Biofarma Research Group, CIMUS Research Center, Departamento de Farmacoloxía, Farmacia e Tecnoloxía Farmacéutica, University of Santiago de Compostela (USC), 15782 Santiago de Compostela, Spain; mabel.loza@usc.es

**Keywords:** amide, benzohomoadamantane, DMPK properties, piperidine, soluble epoxide hydrolase

## Abstract

The pharmacological inhibition of soluble epoxide hydrolase (sEH) has been suggested as a potential therapy for the treatment of pain and inflammatory diseases through the stabilization of endogenous epoxyeicosatrienoic acids. Numerous potent sEH inhibitors (sEHI) have been developed, however many contain highly lipophilic substituents limiting their availability. Recently, a new series of benzohomoadamantane-based ureas endowed with potent inhibitory activity for the human and murine sEH was reported. However, their very low microsomal stability prevented further development. Herein, a new series of benzohomoadamantane-based amides were synthetized, fully characterized, and evaluated as sEHI. Most of these amides were endowed with excellent inhibitory potencies. A selected compound displayed anti-inflammatory effects with higher effectiveness than the reference sEHI, TPPU.

## 1. Introduction

**Figure 1:**
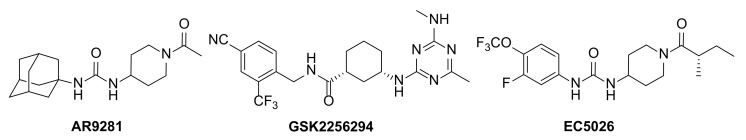
Structure of the three sEHI that have entered human clinical trials.

Soluble epoxide hydrolase (sEH, EPHX2, EC 3.3.2.3) metabolizes epoxyeicosatrienoic acids (EETs), potent endogenous anti-inflammatory mediators [1,2,3], into the corresponding dihydroxyeicosatrienoic acids, which are much less biologically active [4,5]. It is now well established that sEH pharmacological inhibition in vivo stabilizes the concentration of EETs and other epoxy fatty acids, reducing pain and inflammatory states, suggesting sEH as a potential pharmacological target for the treatment of a variety of inflammatory diseases [6,7,8]. Indeed, several sEH inhibitors (sEHI) have reached clinical trials: AR9281, developed by Arête Therapeutics for the treatment of hypertension in diabetic patients [9]; GSK2256294, developed by GlaxoSmithKline for chronic obstructive pulmonary disease [10] and aneurysmal subarachnoid hemorrhage [11]; and EC5026, developed by EicOsis, which has recently successfully finished Phase 1a clinical trials for the treatment of neuropathic pain [12,13] (Figure 1).

**Figure 2:**
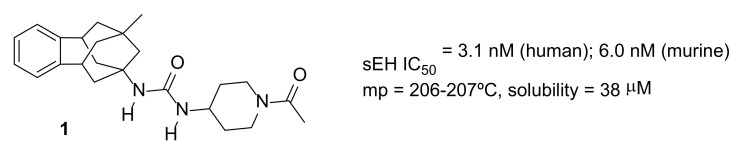
Structure and some properties of **1**. The microsomal stability (% remaining at 1 h) of **1** was 1% in human and in 0.5% in mouse.

Taking into account that several adamantane-based and benzene-based ureas are endowed with very potent activity as sEHI [14,15,16,17], that both AR9281 and EC5026 feature an acylpiperidine unit, and that the highly hydrophobic pocket of sEH seems able to accommodate very large hydrophobic groups [18], we have recently designed, synthesized and pharmacologically evaluated a novel series of ureas featuring the benzohomoadamantane scaffold as hydrophobic moiety, that merges in its polycyclic structure an adamantane-related core with an aromatic ring [19]. Several of these novel sEHI-based ureas were low nanomolar inhibitors of the human and murine sEH, but most of them, as **1**, showed high melting points, limited solubility, and unacceptably low microsomal stabilities (Figure 2) [19]. Taking into account that amides are good pharmacophores for sEHI [20], the aim of the present study was to replace the urea moiety of **1** by an amide group in order to improve the physical properties and, particularly, its very poor microsomal stability to improve availability and in vivo efficacy.

## 2. Results and Discussion

### 2.1. Synthesis of the New sEHI

**Scheme 1:**
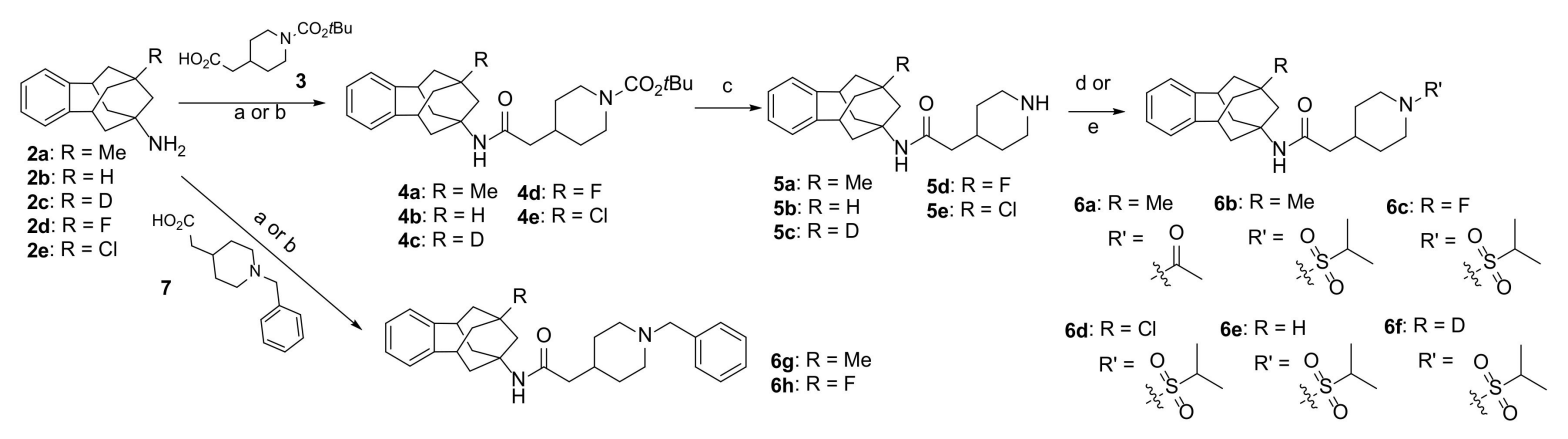
Synthesis of sEHI amides **6a**–**h** from polycyclic amines **2a**–**e**. (**a**) EDC·HCl, HOBt, Et_3_N, EtOAc, rt, 24 h (**4a**–**b**, **4d**); (**b**) HATU, DIPEA, DMF, rt, overnight (**4c**, **4e**, **6g**–**h**); (**c**) HCl/Dioxane, rt, 2 h (**5a**–**e**); (**d**) acetyl chloride, Et_3_N, DCM, 0 °C to rt, overnight (**6a**); (**e**) propane-2-sulfonyl chloride, Et_3_N, DCM, 0 °C to rt, overnight (**6b**–**f**).

The targeted amides were easily synthesized in low to moderate yields from known benzohomoadamantane amines **2a**–**e [21,22,23]**. The coupling of these amines with carboxylic acids **3** and **7**, using either EDC·HCl and HOBt or HATU, provided carbamates **4a**–**e** and amides **6g**–**h**, respectively. Compounds **4a**–**e** were deprotected using HCl in dioxane to give amides **5a**–**e**. Reaction of **5a** with acetyl chloride furnished amide **6a**. Finally, piperidines **5a**–**e** were reacted with 2-propanesulfonyl chloride to obtain amides **6b**–**f** (Scheme 1).

**Scheme 2:**
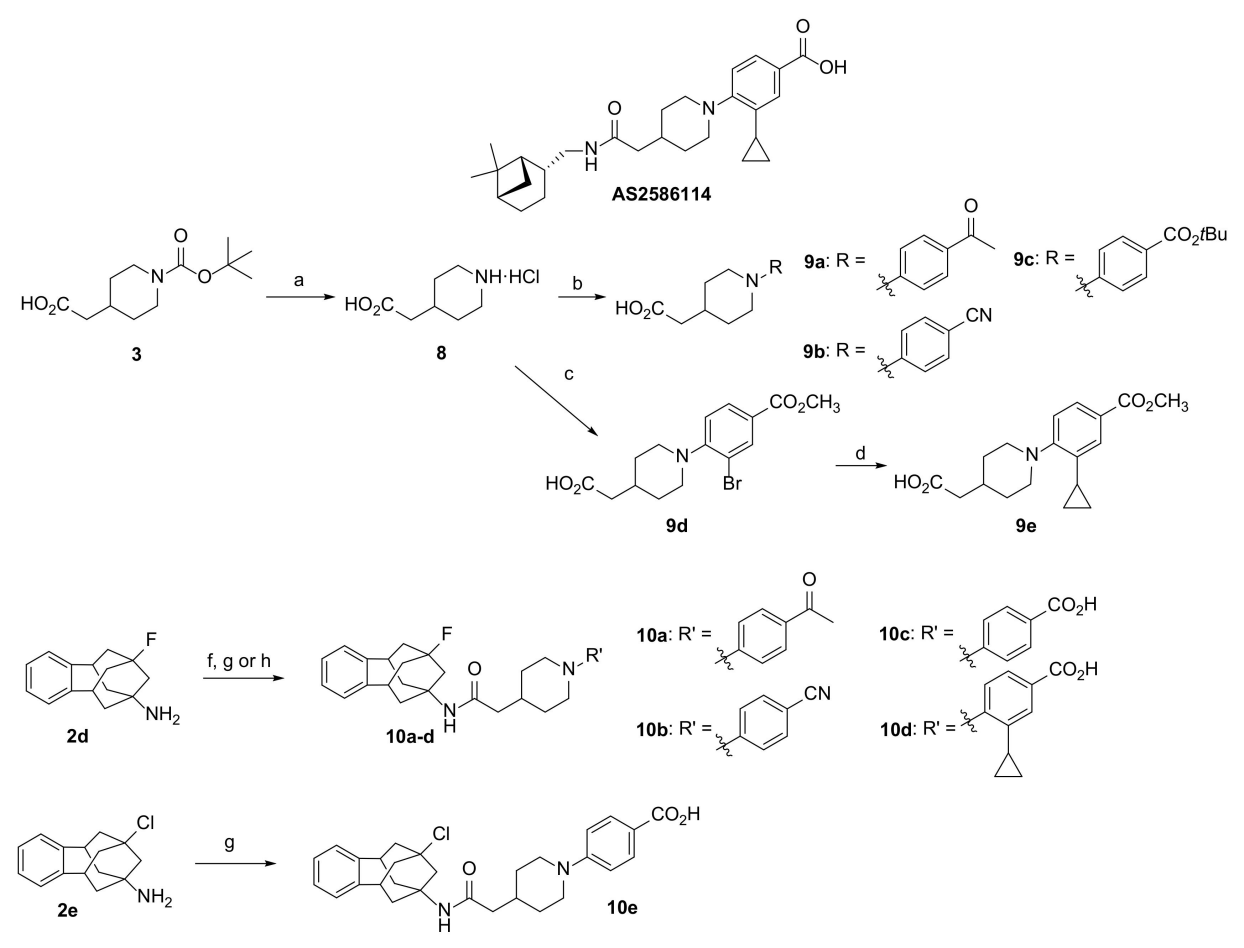
Structure of known AS2586114 and synthesis of novel sEHI **10a–e**. (**a**) HCl/Dioxane 4 M, rt, 2 h; (**b**) 4-fluoroacetophenone (for **9a**), 4-fluorobenzonitrile (for **9b**) or t-butyl 4-fluorobenzoate (for **9c**), K_2_CO_3_, DMSO, 100 °C, overnight; (**c**) methyl 3-bromo-4-fluorobenzoate, K_2_CO_3_, DMF, 100 °C, overnight; (**d**) cyclopropylboronic acid, Pd(Ph_3_)_4_, K_3_PO_4_, dioxane, 100 °C, overnight; (**f**) **9a** or **9b**, HATU, DIPEA, DMF, rt, overnight; (**g**) **9c**, HATU, DIPEA, DMF, rt, overnight; then HCl/dioxane 4 M, H_2_O, rt, 2 h. (**h**) **9e**, HATU, DIPEA, DMF, rt, overnight; then methanol, KOH, rt, overnight.

On the other hand, inspired by the structure of amide AS2586114, a very potent sEHI developed by Astellas [24,25], we explored the series of N-aryl derivatives **10a–e** (Scheme 2). The removal of the Boc group from the commercially available carboxylic acid **3** using HCl in dioxane, as previously reported [26], provided amino acid **8** [27]. Afterwards, nucleophilic aromatic substitution reactions furnished compounds **9a–d** in moderate yields. Reaction of **9d** with cyclopropylboronic acid via a Suzuki coupling afforded **9e** a good yield. The coupling of amine **2d** with carboxylic acids **9a–b** yielded final compounds **10a–b**. Furthermore, the reaction of **2d** with carboxylic acids **9c** and **9e** followed by hydrolysis of the intermediate carboxylic ester furnished carboxylic acids **10c** and **10d**, respectively. Similarly, the reaction of amine **2e** with **9c** followed by hydrolysis led to **10e**.

### 2.2. sEH Inhibition and Microsomal Stability

The potency of all the new compounds as inhibitors of the human and murine sEH was tested using a previously reported sensitive fluorescent-based assay using baculovirus expressed recombinant human and mouse sEH [28]. As shown in Table 1, known urea **1** is a low nanomolar inhibitor of the human (IC_50_ = 3.1 nM) and murine sEH (IC_50_ = 6.0 nM) but endowed with very poor microsomal stability. Previous studies with other families of sEHI have shown that amides are suitable pharmacophores for sEHI. For these families of compounds, the amide function significantly improves the physical properties compared to the corresponding ureas, such as increasing solubility and reducing melting points [17,20]. For this reason, we first explored the effect of replacing the urea group of **1** by an amide, leading to compound **6a**. Typically, previous structure–activity relationship (SAR) studies indicated that the inhibition potencies of amides do not affect mouse sEH compared to the corresponding ureas, whereas this change leads to reduced inhibition potency for human sEH [20]. Indeed, amide **6a** was a low nanomolar inhibitor of murine sEH (IC_50_ = 0.4 nM) but was less potent against the human enzyme (IC_50_ = 34.5 nM). Furthermore, as expected, the melting point of **6a** (mp 85–86 °C) was lower than that of the corresponding urea **1** (mp 206–207 °C). Unfortunately, microsomal stability of **6a** was still very low (Table 1).

Next, we briefly explored the right-hand side (RHS) of the molecule. Thus, the acyl group of **6a** was replaced by either an isopropylsulfonyl group or a benzyl group to obtain compounds **6b** and **6g**, respectively. Both compounds showed potencies in the same order than **6a**, but again presented unacceptable microsomal stabilities.

Separately and noteworthy, we have recently found in a related series of sEHI that the substitution of the methyl group at C-9 of the polycyclic structure in **11a** by halogen atoms, as in **11b** and **11c**, led to a significative increase in the microsomal stability, particularly in murine microsomes (Table 2) [19].

Thus, we synthesized and evaluated halogenated compounds **6c** and **6d** with the aim of improving the microsomal stability of **6b**. Interestingly, this modification resulted in an improvement in the potency on the human enzyme, but only a very moderate enhancement in the microsomal stability (Table 1). Two further derivatives were synthesized, **6e** and **6f**, bearing a hydrogen and a deuterium atom at C-9, respectively. Once again, both compounds were shown to be excellent sEHI in the human and murine enzymes, but the microsomal activity did not improve.

Considering that within this series of amides, **6c** was the compound with the highest human and murine microsomal stability, we selected the fluorinated amine **2d** as the starting material for further SAR studies, keeping constant the left-hand side of the molecule and modifying the RHS. First, we replaced the isopropylsulfonyl group of **6c** by a benzyl unit, leading to **6h**. Unfortunately, this change led to a reduction in the potency and, once again, no improvement over the stabilities of the C-methyl analog, **6g**, was observed.

Next, we explored the series of N-aryl derivatives **10a–d** (Scheme 2). All these compounds were endowed with sub-nanomolar potency against human and murine sEH, and, interestingly, benzoic acid **10c** emerged as a better compound, with improved microsomal stabilities at human and mice. Finally, we synthesized the chlorinated analog **10e**, another potent sEH inhibitor, but with lower microsomal stabilities than **10c** (Table 1).

### 2.3. Cytotoxicity and Anti-Inflammatory Properties of ***10c***

Taking into account the high potency and improved microsomal stability of **10c**, we evaluated its cytotoxicity in SH-SY5Y neuroblastoma cells by propidium iodide (PI) staining after 24 h of incubation. Neither **10c** nor the well-known sEHI TPPU [29] showed cytotoxicity at the highest concentration tested (100 µM). Namely, the calculated percentages of cell death were similar to that of the control treatment (vehicle: DMSO 0.1%) for both tested compounds, **10c** and TPPU (n = 10–12).

It is well known that the pro-inflammatory agent lipopolysaccharide (LPS), a component of the bacterial wall, induces a phenotypic change in the macrophages and in the brain microglia, the main players of the innate immune system. These cells become reactive to fight the infection and show increased phagocytic activity, release of oxygen species and release of inflammatory mediators, including nitric oxide, pro-inflammatory cytokines, and eicosanoids. However, dysregulation in the immune response with age and infections may result in age-related ailments and progressive neurodegeneration [30]. Of note, nitric oxide is a key signaling molecule, which increased generation by the inducible nitric oxide synthase (iNOS) enzyme may initiate deleterious inflammatory processes [31].

**Figure 3:**
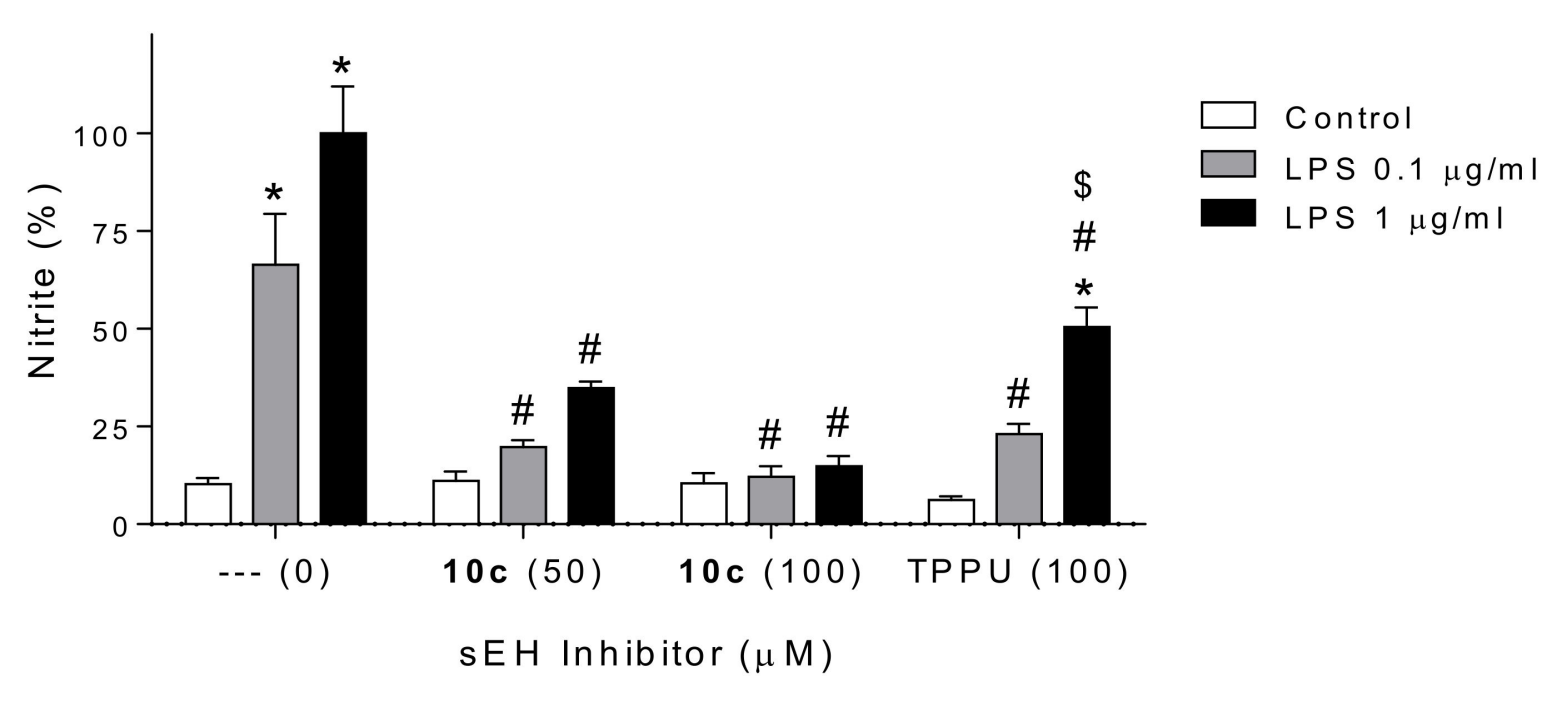
Anti-inflammatory effects of **10c** in activated BV2 microglial cells. Nitrite levels in the culture media, that indicate nitric oxide generation induced by LPS, were decreased to cell resting levels by co-incubation with **10c**. However, TPPU was less effective and lead to a partial decrease. Values are mean ±SEM of n = 10–15. Statistics: * *p* < 0.001 compared to the corresponding control group without LPS; # *p* < 0.001 compared to the corresponding LPS group without anti-inflammatory agents; ^$^ *p* < 0.001 compared to the corresponding LPS concentration treated with **10c**.

In order to evaluate the ability of the selected **10c** in inhibiting the nitric oxide generation by activated glial cells [32] we used microglial BV2 cells activated with LPS. As shown in Figure 3, treatment with LPS led to an increased release of nitric oxide as indicated by the analysis of nitrite levels in the conditioned media. Gratifyingly, co-incubation with **10c** completely inhibited the pro-inflammatory effects of LPS. Statistical comparison of means showed that **10c** at 50 µM or 100 µM reduced nitric oxide generation to levels indistinguishable from control levels despite the presence of LPS. In contrast, the reference compound TPPU was less effective and only partially inhibited LPS effects. TPPU at 100 µM reduced the nitric oxide release induced by LPS to approximately 50%, which was significantly higher than control wells. Notably, the statistical comparison of nitrite levels between both sEHI compounds showed significantly higher protection by **10c** than TPPU against inflammatory cell injury by 1 µg/mL of LPS.

## 3. Materials and Methods

### 3.1. Chemical Synthesis

#### 3.1.1. General Methods

Commercially available reagents and solvents were used without further purification unless stated otherwise. Preparative normal phase chromatography was performed on a CombiFlash Rf 150 (Teledyne Isco) with pre-packed RediSep Rf silica gel cartridges. Thin-layer chromatography was performed with aluminum-backed sheets with silica gel 60 F254 (Merck, ref 1.05554), and spots were visualized with UV light and 1% aqueous solution of KMnO_4_. Melting points were determined in open capillary tubes with an MFB 595010M Gallenkamp. Next, 400 MHz 1H and 100.6 MHz 13C NMR spectra were recorded on a Varian Mercury 400 or on a Bruker 400 Avance III spectrometers. The chemical shifts are reported in ppm (δ scale) relative to internal tetramethylsilane, and coupling constants are reported in Hertz (Hz). Assignments given for the NMR spectra of selected new compounds have been carried out on the basis of DEPT, COSY ^1^H/^1^H (standard procedures), and COSY ^1^H/^13^C (gHSQC and gHMBC sequences) experiments. IR spectra were run on Perkin-Elmer Spectrum RX I, Perkin-Elmer Spectrum TWO or Nicolet Avatar 320 FT-IR spectrophotometers. Absorption values are expressed as wavenumbers (cm^−1^); only significant absorption bands are given. High-resolution mass spectrometry (HRMS) analyses were performed with an LC/MSD TOF Agilent Technologies spectrometer. The elemental analyses were carried out in a Flash 1112 series Thermofinnigan elemental microanalyzer (A5) to determine C, H and N. The structure of all new compounds was confirmed by elemental analysis and/or accurate mass measurement, IR, ^1^H NMR, and ^13^C NMR (check them in Supplementary Materials). The analytical samples of all the new compounds, which were subjected to pharmacological evaluation, possessed purity ≥95% as evidenced by their elemental analyses.

#### 3.1.2. Synthesis of t-Butyl 4-(2-((9-Methyl-5,6,8,9,10,11-hexahydro-7*H*-5,9:7,11-dimethanobenzo[9]annulen-7-yl)amino)-2-oxoethyl)piperidine-1-carboxylate, **4a**

To a suspension of 9-methyl-5,6,8,9,10,11-hexahydro-7*H*-5,9:7,11-dimethanobenzo[9]annulen-7-amine hydrochloride (500 mg, 1.89 mmol), **2a**, in EtOAc (5 mL), 2-(1-(*t*-butoxycarbonyl)piperidin-4-yl)acetic acid (461 mg, 1.89 mmol), **3**, HOBt (384 mg, 2.84 mmol), EDC·HCl (440 mg, 2.84 mmol), and Et_3_N (767 mg, 7.58 mmol) were added. The mixture was stirred at room temperature for 24 h. Water (10 mL) and DCM (20 mL) were added to the resulting suspension and the two phases were separated. The organic phase was washed with sat. NaHCO_3_ aqueous solution (10 mL), brine (10 mL), 2N HCl solution (10 mL) and 2N NaOH (10 mL), dried over anh. Na_2_SO_4_, filtered, and concentrated under vacuum to give **4a** as a yellow solid (515 mg, 60% yield). ^1^H-NMR (400 MHz, CDCl_3_) δ: 0.92 (s, 3 H), 1.11 (dq, *J* = 4.4 Hz, *J’* = 11.6 Hz, 2 H), 1.4 (s, 9 H), 1.54 (d, *J* = 13.6 Hz, 2 H), 1.63–1.68 (complex signal, 4 H), 1.84 (s, 2 H), 1.91 (m, 1 H), 1.97 (s, 2 H), 2.0 (d, *J* = 12.8 Hz, 2 H), 2.14–2.18 (complex signal, 2 H), 2.69 (t, *J* = 13.2 Hz, 2 H), 3.06 (t, *J* = 6 Hz, 2 H), 4.06 (broad signal, 2 H), 5.14 (s, 1 H), 7.02–7.08 (complex signal, 4 H).

#### 3.1.3. Synthesis of *N*-(9-Methyl-5,6,8,9,10,11-hexahydro-7*H*-5,9:7,11-dimethanobenzo[9]annulen-7-yl)-2-(piperidin-4-yl)acetamide, **5a**

To a solution of **4a** (250 mg, 0.55 mmol) in DCM (4 mL) was added 4M HCl in 1,4-dioxane (0.5 mL). The reaction mixture was stirred at room temperature for 3 days. Then, the solvent was evaporated under vacuum and the residue was dissolved in DCM (10 mL) and washed with 5N NaOH solution, dried over anh. Na_2_SO_4_, filtered, and concentrated under vacuum to give **5a** as a yellow solid (189 mg, 97% yield). ^1^H-NMR (400 MHz, CDCl_3_) δ: 0.91 (s, 3 H), 1.12 (dq, *J* = 4 Hz, *J’* = 12.0 Hz, 2 H), 1.53 (d, *J* = 13.2 Hz, 2 H), 1.62–1.71 (complex signal, 4 H), 1.84 (s, 2 H), 1.88 (m, 1 H), 1.95–2.01 (complex signal, 4 H), 2.14–2.19 (complex signal, 2 H), 2.6 (dt, *J* = 2.8 Hz, *J’* = 12.0 Hz, 2 H), 3.00–3.07 (complex signal, 4 H), 5.15 (s, 1 H), 7.02–7.09 (complex signal, 4 H).

#### 3.1.4. Synthesis of *N*-(5,6,8,9,10,11-Hexahydro-7*H*-5,9:7,11-dimethanobenzo[9]annulen-7-yl)-2-(piperidin-4-yl)acetamide, **5b**

To a suspension of 5,6,8,9,10,11-hexahydro-*7H*-5,9:7,11-dimethanobenzo[9]annulen-7-amine (90 mg, 0.42 mmol) **2b**, in DMF (2 mL), 2-(1-(*t*-butoxycarbonyl)piperidin-4-yl)acetic acid (123 mg, 0.51 mmol), **3**, HATU (239 mg, 0.63 mmol), and DIPEA (162 mg, 1.26 mmol) were added. The mixture was stirred at room temperature for 24 h. Solvent was concentrated *in vacuo* and EtOAc (10 mL) was added. The mixture was washed with NaHCO_3_ sat. (2 × 30 mL). The organic phase was dried over anh. Na_2_SO_4_, filtered, and concentrated under vacuum to give carbamate **4b** (185 mg) as a yellow gum that was used as such without further purification. HCl 4 M in dioxane (2 mL) and dioxane (2 mL) were added to the previous gum of **4b** and the mixture was stirred at room temperature for 2 h. Na_2_CO_3_ sat. was added until pH = 12 followed by EtOAc (15 mL) and the mixture was partitioned. The aqueous layer was extracted with EtOAc/MeOH 9/1 (2 × 10 mL). All organic phases were joined, dried over anh. Na_2_SO_4_, filtered, and concentrated under vacuum. The resulting crude was purified by column chromatography in silica gel (SiO_2_, DCM/Methanol mixtures) and gave **5b** as an orangish solid (108 mg, 72% overall yield), mp 185–186 °C. IR (ATR) *v*: 3301, 2907, 2855, 2349, 1653, 1556, 1450, 1356, 1320, 1280, 1137, 1046, 951, 929, 794, 765, 737, 660, 631, 616 cm^−1^. ^1^H-NMR (400 MHz, CD_3_OD) δ: 1.20 [m, 2 H, 3′′(5′′)-H_ax_], 1.63–1.77 [complex signal, 4 H, 10′(13′)-H_ax_, 3′′(5′′)-H_eq_], 1.83 (m, 1 H, 4′′-H), 1.98 [m, 2 H, 10′(13′)-H_eq_], 2.02 (d, *J* = 7.2 Hz, 2 H, 8′-H), 2.06 (m, 2 H, 2-H), 2.12–2.26 [complex signal, 4 H, 6′(12′)-H], 2.32 (m, 1 H, 9′-H), 2.59 [m, 2 H, 2′′(6′′)-H_ax_], 2.98–3.08 [complex signal, 4 H, 5′(11′)-H, 2′′(6′′)-H_eq_], 7.03 [s, 4 H, 1′(4′)-H, 2′(3′)-H]. ^13^C-NMR (100.6 MHz, CD_3_OD) δ: 32.6 (CH, C9′), 33.1 [CH_2_, C3′′(5′′)], 35.0 (CH, C4′′), 35.7 [CH_2_, C10′(13′)], 40.4 [CH_2_, C6′(12′)], 41.5 (CH_2_, C2), 42.6 [CH, C5′(11′)], 45.2 (CH_2_, C8′), 46.7 [CH_2_, 2′′(6′′)], 53.8 (C, C7′), 127.3 [CH, C2′(3′)], 129.0 [CH, C1′(4′)], 148.0 [C, C4a’(11a’)], 173.9 (C, CO). HRMS: Calcd for [C_22_H_30_N_2_O + H]^+^: 339.2438, found: 339.2431.

#### 3.1.5. Synthesis of *N*-(5,6,8,9,10,11-Hexahydro-7*H*-5,9:7,11-dimethanobenzo[9]annulen-7-yl-9-d)-2-(piperidin-4-yl)acetamide, **5c**

To a suspension of 5,6,8,9,10,11-hexahydro-7*H*-5,9:7,11-dimethanobenzo[9]annulen-9-d-7-amine (90 mg, 0.42 mmol), **2c**, in DMF (2 mL), 2-(1-(*t*-butoxycarbonyl)piperidin-4-yl)acetic acid (123 mg, 0.51 mmol), **3**, HATU (239 mg, 0.63 mmol), and DIPEA (162 mg, 1.26 mmol) were added. The mixture was stirred at room temperature for 24 h. Solvent was concentrated *in vacuo* and EtOAc (10 mL) was added. The mixture was washed with NaHCO_3_ sat. (2 x 30 mL). The organic phase was dried over anh. Na_2_SO_4_, filtered, and concentrated under vacuum to give **4c** (185 mg) as a yellow gum that was used as such without further purification. HCl 4 M in dioxane (2 mL) and dioxane (2 mL) were added to the aforementioned gum of **4c** (185 mg, 0.42 mmol), and the mixture was stirred at room temperature for 2 h. Na_2_CO_3_ sat. was added until pH = 12 followed by EtOAc (15 mL) and the mixture was partitioned. The aqueous layer was extracted with EtOAc/MeOH 9/1 (2 × 10 mL). All organic phases were joined, dried over anh. Na_2_SO_4_, filtered, and concentrated under vacuum. The resulting crude was purified by column chromatography in silica gel (SiO_2_, DCM/Methanol mixtures) and gave **5c** as an orangish solid (129 mg, 90% yield), mp: 188–189 °C. IR (ATR) *v*: 3300, 2909, 2852, 2351, 1654, 1555, 1492, 1449, 1356, 1297, 1280, 1046, 928, 840, 794, 764, 737, 655, 616 cm^−1^. ^1^H-NMR (400 MHz, CD_3_OD) δ: 1.24 [m, 2 H, 3′′(5′′)-H_ax_], 1.69–1.76 [complex signal, 4 H, 10′(13′)-H_ax_, 3′′(5′′)-H_eq_], 1.86 (m, 1 H, 4′′-H), 1.99 [m, 2 H, 10′(13′)-H_eq_], 2.02–2.07 (complex signal, 4 H, 2-H, 8′-H), 2.19 [m, 4 H, 6′(12′)-H], 2.66 (dt, *J* = 12.6 Hz, *J’* = 2.8 Hz, 2 H, 2′′(6′′)-H_ax_), 3.04 [m, 2 H, 5′(11′)-H], 3.09 [dt, *J* = 12.6 Hz, *J’* = 2.8 Hz, 2 H, 2′′(6′′)-H_eq_], 7.03 [s, 4 H, 1′(4′)-H, 2′(3′)-H]. ^13^C-NMR (100.6 MHz, CD_3_OD) δ: 32.4 [CH_2_, C3′′(5′′)], 32.5 (m, CD, C9′), 34.5 (CH, C4′′), 35.6 [CH_2_, C10′(13′)], 40.4 [CH_2_, C6′(12′)], 41.4 (CH_2_, C2), 42.6 [CH, C5′(11′)], 44.9 (CH_2_, C8′), 46.4 [CH_2_, 2′′(6′′)], 53.8 (C, C7′), 127.3 [CH, C2′(3′)], 129.0 [CH, C1′(4′)], 148.0 [C, C4a’(11a’)], 173.7 (C, CO). HRMS: Calcd for [C_22_H_29_DN_2_O + H]^+^: 340.2494, found: 340.2500.

#### 3.1.6. Synthesis of *N*-(9-Fluoro-5,6,8,9,10,11-hexahydro-7*H*-5,9:7,11-dimethanobenzo[9]annulen-7-yl)-2-(piperidin-4-yl)acetamide, **5d**

To a suspension of 9-fluoro-5,6,8,9,10,11-hexahydro-7*H*-5,9:7,11-dimethanobenzo[9]annulen-7-amine hydrochloride (200 mg, 0.75 mmol), **2d**, in DMF (3 mL), 2-(1-(*t*-butoxycarbonyl)piperidin-4-yl)acetic acid (218 mg, 0.90 mmol), HATU (430 mg, 1.13 mmol), and DIPEA (386 mg, 3.00 mmol) were added. The mixture was stirred at room temperature for 24 h. Solvent was concentrated in vacuo and EtOAc (15 mL) was added. The mixture was washed with NaHCO_3_ sat. (2 × 30 mL). The organic phase was dried over anh. Na_2_SO_4_, filtered, and concentrated under vacuum. The resulting crude of **4d** was used as such without further purification. ^1^H-NMR (400 MHz, CDCl_3_) δ: 1.10 (m, 2 H), 1.44 (s, 9 H), 1.47 (s, 2 H), 1.66 (d, *J* = 13.5 Hz, 2 H), 1.87–2.05 (complex signal, 6 H), 2.09–2.24 (complex signal, 5 H), 2.27 (d, *J* = 6.2 Hz, 2 H), 2.68 (m, 2 H), 3.23 (m, 2 H), 5.23 (s, 1 H), 7.05–7.15 (complex signal, 4 H). HCl 4 M in dioxane (2 mL), and dioxane (2 mL) were added to **4d** (341 mg, 0.75 mmol) and the mixture was stirred at room temperature for 4 h. Na_2_CO_3_ sat. was added until pH = 12 followed by EtOAc (20 mL) and the mixture was partitioned. The aqueous layer was extracted with EtOAc/MeOH 9/1 (2 × 15 mL). All organic phases were joined, dried over anh. Na_2_SO_4_, filtered, and concentrated under vacuum. The resulting crude was purified by column chromatography in silica gel (SiO_2_, DCM/Methanol/NH_3_ mixtures) and gave **5d** as a yellowish solid (186 mg, 70% yield). ^1^H NMR (400 MHz, DMSO-*d*_6_) δ: 0.98 (m, 2 H), 1.50 (m, 2 H), 1.72 (m, 2 H), 1.90 (d, *J* = 7.2 Hz, 2 H), 1.98–2.02 (complex signal, 4 H), 2.06–2.13 (complex signal, 4 H), 2.39 (dm, *J* = 12.0 Hz, 2 H), 2.86 (dm, *J* = 12.0 Hz, 2 H), 3.21 (s, 2 H), 7.11 (s, 4 H), 7.61 (s, 1 H).

#### 3.1.7. Synthesis of *N*-(9-Chloro-5,6,8,9,10,11-hexahydro-7*H*-5,9:7,11-dimethanobenzo[9]annulen-7-yl)-2-(piperidin-4-yl)acetamide Hydrochloride, **5e**

To a suspension of 9-chloro-5,6,8,9,10,11-hexahydro-7*H*-5,9:7,11-dimethanobenzo[9]annulen-7-amine hydrochloride (178 mg, 0.63 mmol), **2e**, in DMF (3 mL), 2-(1-(*t*-butoxycarbonyl)piperidin-4-yl)acetic acid (183 mg, 0.75 mmol), HATU (357 mg, 0.94 mmol), and DIPEA (326 mg, 2.52 mmol) were added. The mixture was stirred at room temperature for 6 h. Solvent was concentrated in vacuo and EtOAc (15 mL) was added. The mixture was washed with NaHCO_3_ sat. (2 × 30 mL). The organic phase was dried over anh. Na_2_SO_4_, filtered, and concentrated under vacuum. The resulting crude of **4e** was used as such without further purification. ^1^H-NMR (400 MHz, CDCl_3_) δ: 1.44 (s, 9 H), 1.69 (m, 2 H), 1.91 (m, 1 H), 1.95–2.01 (complex signal, 4 H), 2.04 (s, 1 H), 2.12–2.32 (complex signal, 6 H), 2.38 (m, 2 H), 2.53 (s, 1 H), 2.70 (d, *J* = 12.1 Hz, 2 H), 3.17 (t, *J* = 6.5 Hz, 2 H), 4.07 (m, 2 H), 5.21 (s, 1 H), 7.04–7.15 (complex signal, 4 H). HCl 4 M in dioxane (2 mL) and dioxane (2 mL) were added to **4e** (296 mg, 0.63 mmol) and the mixture was stirred at room temperature overnight. Saturated aqueous NaHCO_3_ solution (20 mL) was added followed by EtOAc (15 mL) and the mixture was partitioned. The aqueous phase was further extracted with EtOAc/MeOH 9/1 (2 × 15 mL). All organic phases were joined, dried over anh. Na_2_SO_4_, filtered, and solvents were concentrated under vacuum. The resulting crude was purified by column chromatography in silica gel (SiO_2_, DCM/MeOH/NH_3_ mixtures). Fractions containing the desired product were collected and concentrated *in vacuo* to afford a reddish solid. HCl 2 M in Et_2_O (5 mL) was added to form its hydrochloride, followed by filtration of the solid to afford **5e** as an orange solid (84 mg, 33% yield), mp: > 300 °C. IR (ATR) *v*: 3300, 2937, 1655, 1557, 1493, 1450, 1357, 1298, 1280, 1206, 1089, 930, 793, 765, 632, 614 cm^−1^. ^1^H-NMR (400 MHz, CD_3_OD) δ: 1.47 [m, 2 H, 3′′(5′′)-H_ax_], 1.90 [d, *J* = 14.1 Hz, 2 H, 3′′(5′′)-H_eq_], 1.99–2.09 [complex signal, 5 H, 6′(12′)-H_ax_, 10′(13′)-H_ax_, 4′′-H], 2.14 (d, *J* = 7.2 Hz, 2 H, 2-H), 2.22 [dd, *J* = 13.2 Hz, *J’* = 6.0 Hz, 2 H, 6′(12′)-H_eq_], 2.38 [dd, *J* = 13.2 Hz, *J’* = 6.0 Hz, 2 H, 10′(13′)-H_eq_], 2.49 (s, 2 H, 8′-H), 2.97 [t, *J* = 12.5 Hz, 2 H, 2′′(6′′)-H_ax_], 3.18 [broad t, *J* = 6.2 Hz, 2 H, 5′(11′)-H], 3.36 [d, *J* = 12.4 Hz, 2 H, 2′′(6′′)-H_eq_], 7.03–7.14 [complex signal, 4 H, 1′(4′)-H, 2′(3′)-H]. ^13^C-NMR (100.6 MHz, CD_3_OD) δ: 29.5 [CH_2_, C3′′(5′′)], 32.7 (CH, C4′′), 38.9 [CH_2_, C6′(12′)], 42.5 [CH, C5′(11′)], 43.7 (CH_2_, C2), 45.1 [CH_2_, 2′′(6′′)], 45.9 [CH_2_, C10′(13′)], 50.9 (CH_2_, C8′), 57.6 (C, C7′), 70.2 (C, C9′), 127.9 [CH, C2′(3′)], 129.1 [CH, C1′(4′)], 146.1 [C, C4a’(11a’)], 173.1 (C, CO). Anal. Calcd for C_22_H_29_ClN_2_O · 1 HCl: C 64.54, H 7.39, N 6.84. Calcd for C_22_H_29_ClN_2_O · 2 HCl: C 59.27, H 7.01, N 6.28. Found: C 59.30, H 7.00, N 6.37. HRMS: Calcd for [C_22_H_29_ClN_2_O + H]^+^: 373.2041, found: 373.2047.

#### 3.1.8. Synthesis of 2-(1-Acetylpiperidin-4-yl)-*N*-(9-methyl-5,6,8,9,10,11-hexahydro-7*H*-5,9:7,11-dimethanobenzo[9]annulen-7-yl)acetamide, **6a**

To a solution of **5a** (200 mg, 0.57 mmol) in anh. DCM (5 mL) under argon atmosphere was added anh. Et_3_N (69 mg, 0.68 mmol). The mixture was cooled down to 0°C and acetyl chloride (45 mg, 0.57 mmol) was added dropwise. Then, the reaction mixture was stirred at room temperature overnight and quenched by addition of 2N HCl solution (3 mL). The two phases were separated, and the aqueous layer was extracted with EtOAc (2 × 20 mL). The combined organic phases were washed with 2N NaOH solution, dried over anh. Na_2_SO_4_, filtered, and concentrated under vacuum. Column chromatography (SiO_2_, Hexane/EtOAc mixtures) gave **6a** as a white solid (134 mg, 60% yield), mp 85–86 °C. IR (NaCl disk): 3315, 3060, 3017, 2916, 2860, 1631, 1544, 1493, 1450, 1361, 1304, 1273, 1241, 1197, 1165, 1138, 1096, 1048 cm^−1^. ^1^H-NMR (400 MHz, CDCl_3_) δ: 0.91 (s, 3 H, C9′′-CH_3_), 1.10 [m, 2 H, 3′(5′)-H_ax_], 1.53 [d, *J* = 13.6 Hz, 2 H, 10′′(13′′)-H_ax_], 1.65 [dm, *J* = 12.8 Hz, 10′′(13′′)-H_eq_], 1.71 (d, *J* = 12.8 Hz, 1 H, 5′-H_eq_ or 3′-H_eq_), 1.76 (d, *J* = 12.4 Hz, 1 H, 3′-H_eq_ or 5′-H_eq_), 1.83 (s, 2 H, 8′′-H), 1.96–2.04 [complex signal, 5 H, 2-H_2_, 4′-H, 6′′(12′′)-H_ax_], 2.06 (s, 3 H, COCH_3_), 2.15 [m, 2 H, 6′′(12′′)-H_eq_], 2.53 (t, *J* = 12.4 Hz, 1 H, 2′-H_ax_ or 6′-H_ax_), 3.03 (m, 1 H, 6′-H_ax_ or 2′-H_ax_), 3.08 [broad t, *J* = 6.0 Hz, 2 H, 5′′(11′′)-H], 3.76 (d, *J* = 13.6 Hz, 1 H, 6′-H_eq_ or 2′-H_eq_), 4.58 (d, *J* = 13.2 Hz, 1 H, 2′-H_eq_ or 6′-H_eq_), 5.25 (s, 1H, NH), 7.03 [m, 2 H, 1′′(4′′)-H], 7.06 [m, 2 H, 2′′(3′′)-H]. ^13^C-NMR (100.6 MHz, CDCl_3_) δ: 21.5 (CH_3_, COCH_3_), 31.6 (CH_2_, C5′ or C3′), 32.2 (CH_3_, C9-CH_3_), 32.4 (CH_2_, C3′ or C5′), 33.56 (C or CH, C9′′ or C4′), 33.57 (CH or C, C4′ or C9′′), 39.0 (CH_2_, C6′′ or C12′′), 39.1 (CH_2_, C12′′ or C6′′), 40.9 [CH, C5′′(11′′)], 41.1 [CH_2_, C10′′(13′′)], 41.7 (CH_2_, C2′ or C6′), 44.4 (CH_2_, C2), 46.5 (CH_2_, C6′ or C2′), 47.2 (CH_2_, C8′′), 54.7 (C, C7′′), 126.3 [CH, C2′′(3′′)], 128.0 [CH, C1′′(4′′)], 146.1 [C, C4a’’(11a’’)], 168.7 (C, COCH_3_), 170.4 (C, NHCO). Anal. Calcd for C_25_H_34_N_2_O_2_ · 0.5 H_2_O: C 74.41, H 8.74, N 6.94. Found: C 74.36, H 8.79, N 6.74. HRMS: Calcd for [C_25_H_34_N_2_O_2_ + H]^+^: 395.2693; Found: 395.2691.

#### 3.1.9. Synthesis of 2-[1-(Isopropylsulfonyl)piperidin-4-yl]-*N*-(9-methyl-5,6,8,9,10,11-hexahydro-7*H*-5,9:7,11-dimethanobenzo[9]annulen-7-yl)acetamide, **6b**

To a solution of **5a** (185 mg, 0.52 mmol) in DCM (5 mL) was added Et_3_N (63 mg, 0.63 mmol). The mixture was cooled down to 0 °C and propane-2-sulfonyl chloride (74 mg, 0.52 mmol) was added dropwise. Then, the reaction mixture was stirred at room temperature overnight and quenched by an addition of 2N HCl solution (3 mL). The two phases were separated, and the aqueous phase was extracted with EtOAc (2 × 20 mL). The combined organic phases were washed with 5N NaOH solution, dried over anh. Na_2_SO_4_, filtered, and concentrated under vacuum to give a yellow solid. Column chromatography (SiO_2_, Hexane/EtOAc mixtures) gave **6b** as a white solid (145 mg, 60% yield). The analytical sample was obtained by crystallization from hot EtOAc, mp 172 -173 °C. IR (NaCl disk): 3365, 3319, 3059, 3018, 2916, 2852, 1648, 1536, 1493, 1452, 1361, 1323, 1309, 1265, 1190, 1167, 1138, 1091, 1044, 1011, 993, 945, 905, 881, 801, 759, 732, 702, 665 cm^−1^. ^1^H-NMR (400 MHz, CDCl_3_) δ: 0.91 (s, 3 H, C9′′-CH_3_), 1.25 [dq, *J* = 12.0 Hz, *J’* = 4.0 Hz, 2 H, 3′(5′)-H_ax_], 1.31 [d, *J* = 6.8 Hz, 6 H, CH(CH_3_)_2_], 1.53 [d, *J* = 13.6 Hz, 2 H, 10′′(13′′)-H_ax_], 1.65 [dm, *J* = 13.6 Hz, 2 H, 10′′(13′′)-H_eq_], 1.74 [dm, *J* = 12.0 Hz, 2 H, 3′(5′)-H_eq_], 1.82 (s, 2 H, 8′′-H_2_), 1.93 (m, 1 H, 4′-H), 1.97–2.04 [complex signal, 4 H, 2-H_2_, 6′′(12′′)-H_ax_], 2.15 [dd, *J* = 11.6 Hz, *J’* = 6.0 Hz, 6′′(12′′)-H_eq_], 2.85 [dt, *J* = 12.4 Hz, *J’* = 2.4 Hz, 2 H, 2′(6′)-H_ax_], 3.06 [t, *J* = 6.0 Hz, 2 H, 5′′(11′′)-H], 3.15 [hept, *J* = 6.8 Hz, 1 H, CH(CH_3_)_2_], 3.80 [dt, *J* = 12.8 Hz, *J’* = 2.0 Hz, 2 H, 2′(6′)-H_eq_], 5.22 (s, 1 H, NH), 7.03 [m, 2 H, 1′′(4′′)-H], 7.07 [m, 2 H, 2′′(3′′)-H]. ^13^C-NMR (100.6 MHz, CDCl_3_) δ: 16.8 [CH_3_, CH(CH_3_)_2_], 32.2 (CH_3_, C9-CH_3_), 32.3 [CH_2_, C3′(5′)], 33.1 (CH, C4′), 33.6 (C, C9′′), 39.1 [CH_2_, C6′′(12′′)], 41.0 [CH, C5′′(11′′)], 41.1 [CH_2_, C10′′(13′′)], 44.4 (CH_2_, C2), 46.5 [CH_2_, C2′(6′)], 47.2 (CH_2_, C8′′), 53.2 [CH, CH(CH_3_)_2_], 54.7 (C, C7′′), 126.3 [CH, C2′′(3′′)], 128.0 [CH, C1′′(4′′)], 146.1 [C, C4a’’(11a’’)], 170.3 (C, CO). Anal. Calcd for C_26_H_38_N_2_O_3_S: C 68.09, H 8.35, N 6.11. Found: C 67.75 H 8.62, N 5.74. HRMS: Calcd for [C_26_H_38_N_2_O_3_S + H]^+^: 459.2676; Found: 459.2675.

#### 3.1.10. Synthesis of *N*-(9-Fluoro-5,6,8,9,10,11-hexahydro-7*H*-5,9:7,11-dimethanobenzo[9]annulen-7-yl)-2-(1-(isopropylsulfonyl)piperidin-4-yl)acetamide, **6c**

To a solution of **5d** (186 mg, 0.52 mmol) and triethylamine (63 mg, 0.63 mmol) in anh. DCM (2 mL) was added 2-propanesulfonyl chloride (89 mg, 0.63 mmol). Then, the mixture was stirred at room temperature for 24 h. NaHCO_3_ sat. (15 mL) was added followed by EtOAc (15 mL) and the mixture was partitioned. The aqueous layer was extracted again with EtOAc (2 x 15 mL). Both organic phases were joined, dried over Na_2_SO_4_ anh., filtered, and solvents were concentrated under vacuum. The resulting crude was purified by column chromatography in silica gel (SiO_2_, Hexane/EtOAc mixtures) and gave **6c** as a white solid (123 mg, 51% yield), mp: 192–193 °C. IR (ATR) *v*: 3342, 2914, 2855, 1663, 1536, 1449, 1316, 1138, 1045, 1017, 998, 953, 940, 865, 761, 753, 732, 652 cm^−1^. ^1^H-NMR (400 MHz, CDCl_3_) δ: 1.24 [m, 2 H, 3′′(5′′)-H_ax_], 1.31 [d, *J* = 6.9 Hz, 6 H, 2′′′(3′′′)-H], 1.73 [m, 2 H, 3′′(5′′)-H_eq_], 1.88–2.04 [complex signal, 7 H, 2-H, 6′(12′)-H_ax_, 10′(13′)-H_ax_, 4′′-H], 2.10–2.23 [complex signal, 4 H, 6′(12′)-H_eq_, 10′(13′)-H_eq_], 2.26 (d, *J* = 6.3 Hz, 2 H, 8′-H), 2.85 [m, 2 H, 2′′(6′′)-H_ax_], 3.14 (hept, *J* = 6.9 Hz, 1 H, 1′′′-H), 3.23 [m, 2 H, 5′(11′)-H], 3.80 [m, 2 H, 2′′(6′′)-H_eq_], 5.42 (broad s, 1 H, NH), 7.07 [m, 2 H, 1′(4′)-H], 7.13 [m, 2 H, 2′(3′)-H]. ^13^C-NMR (100.6 MHz, CDCl_3_) δ: 16.9 [CH_3_, C2′′′(3′′′)], 32.4 [CH_2_, C3′′(5′′)], 33.2 (CH, C4′′), 38.7 [CH_2_, C6′(12′)], 39.6 [d, *^3^J_CF_* = 13.3 Hz, CH, C5′(11′)], 40.2 [d, *^2^J_CF_* = 20.2 Hz, CH_2_, C10′(13′)], 44.3 (CH_2_, C2), 46.1 (d, *^2^J_CF_* = 18.5 Hz, CH_2_, C8′), 46.7 [CH_2_, C2′′(6′′)], 53.4 (CH, C1′′′), 58.0 (d, *^3^J_CF_* = 11.4 Hz, C, C7′), 94.2 (d, *^1^J_CF_* = 177.6 Hz, C, C9′), 127.1 [CH, C2′(3′)], 128.3 [CH, C1′(4′)], 144.8 [C, C4a(11a)], 170.6 (C, CO). ^19^F-NMR (376 MHz, CDCl_3_) δ: −124.92 (m, 1 F). Anal. Calcd for C_25_H_35_FN_2_O_3_S: C 64.91, H 7.63, N 6.06. Found: C 65.08, H 7.97, N 5.74. HRMS: Calcd for [C_25_H_35_FN_2_O_3_S + H]^+^: 463.2425, found: 463.2425.

#### 3.1.11. Synthesis of *N*-(9-Chloro-5,6,8,9,10,11-hexahydro-7*H*-5,9:7,11-dimethanobenzo[9]annulen-7-yl)-2-(1-(isopropylsulfonyl)piperidin-4-yl)acetamide, **6d**

To a solution of **5d** (75 mg, 0.18 mmol) and triethylamine (73 mg, 0.72 mmol) in anh. DCM (2 mL) was added 2-propanesulfonyl chloride (39 mg, 0.27 mmol). Then, the mixture was stirred at room temperature for 24 h. NaHCO_3_ sat. (15 mL) was added followed by EtOAc (10 mL) and the mixture was partitioned. The aqueous layer was extracted again with EtOAc (2 × 10 mL). Both organic phases were joined, dried over Na_2_SO_4_ anh., filtered, and solvents were concentrated under vacuum. The resulting crude was purified by column chromatography in silica gel (SiO_2_, Hexane/EtOAc mixtures) and gave **6d** as a reddish solid (37 mg, 40% yield), mp: 163–164 °C. IR (ATR) *v*: 3305, 2922, 2906, 2858, 1643, 1548, 1356, 1321, 1276, 1196, 1138, 1058, 1047, 952, 935, 799, 764, 738, 658 cm^−1^. ^1^H-NMR (400 MHz, CD_3_OD) δ: 1.22 [m, 2 H, 3′′(5′′)-H_ax_], 1.29 [d, *J* = 6.8 Hz, 6 H, 2′′′(3′′′)-H], 1.72 [m, 2 H, 3′′(5′′)-H_eq_], 1.88 (m, 1 H, 4-H’’), 2.02–2.12 [complex signal, 6 H, 2-H, 6′(12′)-H_ax_, 10′(13′)-H_ax_], 2.22 [m, 2 H, 6′(12′)-H_eq_], 2.40 [m, 2 H, 10′(13′)-H_eq_], 2.48 (s, 2 H, 8′-H), 2.90 [m, 2 H, 2′′(6′′)-H_ax_], 3.19 [m, 2 H, 5′(11′)-H], 3.28 (hept, *J* = 6.9 Hz, 1 H, 1′′′-H), 3.76 [m, 2 H, 2′′(6′′)-H_eq_], 7.05–7.14 [complex signal, 4 H, 1′(4′), 2′(3′)-H]. ^13^C NMR (100.6 MHz, CD_3_OD) δ: 17.0 [CH_3_, C2′′′(3′′′)], 33.3 [CH_2_, C3′′(5′′)], 34.6 (CH, C4′′), 38.9 [CH_2_, C6′(12′)], 42.6 [CH, C5′(11′)], 44.4 (CH_2_, C2), 45.9 [CH_2_, C10′(13′)], 47.4 [CH_2_, C2′′(6′′)], 51.0 (CH_2_, C8′), 54.0 (CH, C1′′′), 57.6 (C, C7′), 70.3 (C, C9′), 128.0 [CH, C2′(3′)], 129.1 [CH, C1′(4′)], 146.2 [C, C4a(11a)], 173.8 (C, CO). Anal. Calcd for C_25_H_35_ClN_2_O_3_S: C 62.68, H 7.36, N 5.85. Found: C 62.63, H 7.31, N 5.68. HRMS: Calcd for [C_25_H_35_ClN_2_O_3_S + H]^+^: 479.2130, found: 479.2143.

#### 3.1.12. Synthesis of *N*-(5,6,8,9,10,11-Hexahydro-7*H*-5,9:7,11-dimethanobenzo[9]annulen-7-yl)-2-(1-(isopropylsulfonyl)piperidin-4-yl)acetamide, **6e**

To a solution of **5b** (90 mg, 0.27 mmol) and triethylamine (109 mg, 1.08 mmol) in anh. acetonitrile (2 mL) was added 2-propanesulfonyl chloride (76 mg, 0.53 mmol). Then, the mixture was stirred at room temperature for 24 h. NaHCO_3_ sat. (10 mL) was added followed by EtOAc (10 mL) and the mixture was partitioned. The aqueous phase was extracted again with EtOAc (10 mL). Both organic phases were joined, dried over anh. Na_2_SO_4_, filtered, and concentrated under vacuum. The resulting crude was purified by column chromatography in silica gel (SiO_2_, Hexane/EtOAc mixtures) and gave **6e** as a white solid (68 mg, 55% yield), mp: 136–137 °C. IR (ATR) *v*: 3301, 2922, 2858, 1642, 1548, 1355, 1320, 1276, 1195, 1137, 1047, 951, 935, 799, 764, 738, 684, 657, 618 cm^−1^. ^1^H-NMR (400 MHz, CD_3_OD) δ: 1.24 [m, 2 H, 3′′(5′′)-H_ax_], 1.29 [d, *J* = 6.9 Hz, 6 H, 2′′′(3′′′)-H], 1.68–1.77 [complex signal, 4 H, 10′(13′)-H_ax_, 3′′(5′′)-H_eq_], 1.86 (m, 1 H, 4′′-H), 2.00 [m, 2 H, 10′(13′)-H_eq_], 2.02–2.09 (complex signal, 4 H, 2-H, 8′-H), 2.16 [m, 2 H, 6′(12′)-H_ax_], 2.23 [m, 2 H, 6′(12′)-H_eq_], 2.32 (m, 1 H, 9′-H), 2.90 [m, 2 H, 2′′(6′′)-H_ax_], 3.04 [m, 2 H, 5′(11′)-H], 3.26 (hept, *J* = 6.9 Hz, 1 H, 1′′′-H), 3.76 [m, 2 H, 2′′(6′′)-H_eq_], 7.03 [s, 4 H, 1′(4′)-H, 2′(3′)-H]. ^13^C-NMR (100.6 MHz, CD_3_OD) δ: 17.0 [CH_3_, C2′′′(3′′′)], 32.6 (CH, C9′), 33.3 [CH_2_, C3′′(5′′)], 34.6 (CH, C4′′), 35.7 [CH_2_, C10′(13′)], 40.4 [CH_2_, C6′(12′)], 41.4 (CH_2_, C2), 42.6 [CH, C5′(11′)], 44.5 (CH_2_, C8′), 47.4 [CH_2_, 2′′(6′′)], 53.9 (CH, 1′′′C), 54.0 (C, C7′), 127.3 [CH, C2′(3′)], 129.0 [CH, C1′(4′)], 148.0 [C, C4a’(11a’)], 173.6 (C, CO). Anal. Calcd for C_25_H_36_N_2_O_3_S: C 67.53, H 8.16, N 6.30. Calcd for C_25_H_36_N_2_O_3_S · 0.2 H_2_O: C 66.99, H 8.19, N 6.25. Found: C 67.04, H 8.12, N 6.09. HRMS: Calcd for [C_25_H_36_N_2_O_3_S + H]^+^: 445.2528, found: 445.2519.

#### 3.1.13. Synthesis of *N*-(5,6,8,9,10,11-Hexahydro-7*H*-5,9:7,11-dimethanobenzo[9]annulen-7-yl-9-d)-2-(1-(isopropylsulfonyl)piperidin-4-yl)acetamide, **6f**

To a solution of **5c** (111 mg, 0.33 mmol) and triethylamine (134 mg, 1.32 mmol) in anh. Acetonitrile (2 mL) was added 2-propanesulfonyl chloride (93 mg, 0.65 mmol). Then, the mixture was stirred at room temperature for 24 h. NaHCO_3_ sat. (20 mL) was added followed by EtOAc (15 mL) and the mixture was partitioned. The aqueous layer was extracted again with EtOAc (15 mL). Both organic phases were joined, dried over Na_2_SO_4_ anh., filtered, and solvents were concentrated under vacuum. The resulting crude was purified by column chromatography in silica gel (SiO_2_, Hexane/EtOAc mixtures) and gave **6f** as a white solid (97 mg, 63% yield), mp: 138–139 °C. IR (ATR) *v*: 3299, 2922, 2857, 1643, 1549, 1321, 1276, 1138, 1047, 952, 934, 764, 738, 656 cm^−1^. ^1^H-NMR (400 MHz, CD_3_OD) δ: 1.22 [m, 2 H, 3′′(5′′)-H_ax_], 1.29 [d, *J* = 6.9 Hz, 6 H, 2′′′(3′′′)-H], 1.68–1.76 [complex signal, 4 H, 10′(13′)-H_ax_, 3′′(5′′)-H_eq_], 1.86 (m, 1 H, 4′′-H), 1.99 [m, 2 H, 10′(13′)-H_eq_], 2.03–2.08 (complex signal, 4 H, 2-H, 8′-H), 2.16 [m, 2 H, 6′(12′)-H_ax_], 2.23 [m, 2 H, 6′(12′)-H_eq_], 2.89 [m, 2 H, 2′′(6′′)-H_ax_], 3.03 [m, 2 H, 5′(11′)-H], 3.25 (hept, *J* = 6.9 Hz, 1 H, 1′′′-H), 3.76 [m, 2 H, 2′′(6′′)-H_eq_], 7.03 [s, 4 H, 1′(4′)-H, 2′(3′)-H]. ^13^C-NMR (100.6 MHz, CD_3_OD) δ: 17.0 [CH_3_, C2′′′(3′′′)], 32.1 (CD, C9′), 33.3 [CH_2_, C3′′(5′′)], 34.6 (CH, C4′′), 35.6 [CH_2_, C10′(13′)], 40.4 [CH_2_, C6′(12′)], 41.3 (CH_2_, C2), 42.6 [CH, C5′(11′)], 44.5 (CH_2_, C8′), 47.4 [CH_2_, 2′′(6′′)], 53.9 (CH, 1′′′C), 54.0 (C, C7′), 127.3 [CH, C2′(3′)], 129.0 [CH, C1′(4′)], 148.0 [C, C4a’(11a’)], 173.6 (C, CO). Anal. Calcd for C_25_H_35_DN_2_O_3_S: C 67.38, H 8.37, N 6.29. Calcd for C_25_H_35_DN_2_O_3_S · 0.3 H_2_O: C 66.58, H 7.96, N 6.21. Found: C 66.68, H 8.05, N 6.10. HRMS: Calcd for [C_25_H_35_DN_2_O_3_S + H]^+^: 446.2582, found: 446.2589.

#### 3.1.14. Synthesis of 2-(1-Benzylpiperidin-4-yl)-*N*-(9-methyl-5,6,8,9,10,11-hexahydro-7*H*-5,9:7,11-dimethanobenzo[9]annulen-7-yl)acetamide, **6g**

To a suspension of 9-methyl-5,6,8,9,10,11-hexahydro-7*H*-5,9:7,11-dimethanobenzo[9]annulen-7-amine hydrochloride (250 mg, 0.95 mmol), **2a**, in EtOAc (5 mL), 2-(1-benzylpiperidin-4-yl)acetic acid hydrochloride (255 mg, 0.95 mmol), **7**, HOBt (192 mg, 1.42 mmol), EDC·HCl (220 mg, 1.42 mmol) and Et_3_N (480 mg, 4.74 mmol) were added. The mixture was stirred at room temperature for 24 h. Water (10 mL) and DCM (10 mL) were added to the resulting suspension and the 2 phases were separated. The organic phase was washed with sat. NaHCO_3_ aqueous solution (10 mL), brine (10 mL), dried over anh. Na_2_SO_4_, filtered, and concentrated under vacuum to give a yellow gum (479 mg). Column chromatography (SiO_2_, DCM/Methanol mixtures) gave **6g** as a white solid (280 mg, 67% yield). The analytical sample was obtained by crystallization from hot EtOAc and Et_2_O, mp 145–146 °C. IR (NaCl disk): 3302, 3060, 3025, 2917, 2842, 2799, 2756, 1641, 1545, 1493, 1452, 1361, 1343, 1309, 1279, 1211, 1185, 1144, 1078, 1009, 974, 944, 917, 794, 757, 737, 698 cm^−1^. ^1^H-NMR (400 MHz, CDCl_3_) δ: 0.91 (s, 3 H, C9′′-CH_3_), 1.27 [dq, *J* = 12.4 Hz, *J’* = 3.6 Hz, 2 H, 3′(5′)-H_ax_], 1.53 [d, *J* = 13.2 Hz, 2 H, 10′′(13′′)-H_ax_], 1.62–1.70 [complex signal, 4 H, 3′(5′)-H_eq_, 10′’(13′’)-H_eq_], 1.77 (m, 1 H, 4′-H), 1.84 (s, 2 H, 8′′-H), 1.94–2.02 (complex signal, 6 H, 6′′(12′′)-H_ax_, 2-H, 6′(2′)-H_ax_], 2.16 [dd, *J* = 12 Hz, *J’* = 6 Hz, 6′′(12′′)-H_eq_], 2.86 [dt, *J* = 11.6 Hz, *J’* = 2.8 Hz, 2 H, 2′(6′)-H_eq_], 3.06 [t, *J* = 6 Hz, 2 H, 5′′(11′′)-H], 3.48 (s, 2 H, CH_2_-C_6_H_5_), 5.20 (s, 1 H, NH), 7.03 [m, 2 H, 1′′(4′′)-H], 7.06 [m, 2 H, 2′′(3′′)-H], 7.23 (m, 1 H, 4′′′-H), 7.27–7.32 [complex signal, 4 H, 2′′′(6′′′)-H, 3′′′(5′′′)-H]. ^13^C-NMR (100.6 MHz, CDCl_3_) δ: 32.0 [CH_2_, C3′(5′)], 32.2 (CH_3_, C9′′-CH_3_), 33.4 (C, C9′′), 33.5 (CH, C4′), 39.1 [CH_2_, C6′′(12′′)], 40.9 [CH, C5′′(11′′)], 41.1 [CH_2_, C10′′(13′′)], 44.9 (CH_2_, C2), 47.1 (CH_2_, C8′′), 53.5 (CH_2_, C2′(6′)], 54.4 (C, C7′′), 63.3 (CH_2_, CH_2_-C_6_H_5_), 126.2 [CH, C2′′(3′′)], 126.9 (CH, Ar-CH*_para_*), 127.9 [CH, C1′′(4′′)], 128.1 [CH, C3′′′(5′′′)], 129.2 [CH, C2′′′(6′′′)], 138.3 (C, Ar-C*_ipso_*), 146.1 [C, C4a’’(11a’’)], 171.0 (C, NHCO). HRMS: Calcd for [C_30_H_38_N_2_O + H]^+^: 443.3057; Found: 443.3061.

#### 3.1.15. Synthesis of 2-(1-Benzylpiperidin-4-yl)-*N*-(9-fluoro-5,6,8,9,10,11-hexahydro-7*H*-5,9:7,11-dimethanobenzo[9]annulen-7-yl)acetamide, **6h**

To a solution of 9-fluoro-5,6,8,9,10,11-hexahydro-7*H*-5,9:7,11-dimethanobenzo[9]annulen-7-amine (71 mg, 0.31 mmol), **2d**, in DMF (2 mL), 2-(1-benzylpiperidin-4-yl)acetic acid hydrochloride (100 mg, 0.37 mmol), **7**, HATU (176 mg, 0.46 mmol), and DIPEA (119 mg, 0.92 mmol) were added. The mixture was stirred at room temperature for 24 h. Solvent was concentrated in vacuo and EtOAc (10 mL) was added. The mixture was washed with NaHCO_3_ sat. (2 × 20 mL). The organic phase was dried over anh. Na_2_SO_4_, filtered, and concentrated under vacuum. The resulting crude was purified by column chromatography in silica gel (SiO_2_, DCM/MeOH mixtures) and gave **6h** as a brown solid (72 mg, 52% yield), mp: 164–165 °C IR (ATR) *v*: 3317, 2938, 2857, 2808, 2766, 1637, 1548, 1447, 1423, 1360, 1317, 1281, 1135, 1068, 1000, 974, 864, 840, 764, 733, 697, 663, 643, 571, 592 cm^−1^. ^1^H-NMR (400 MHz, CD_3_OD) δ: 1.30 [m, 2 H, 3′′(5′′)-H_ax_], 1.68 [dm, *J* = 12.7 Hz, 2 H, 3′′(5′′)-H_eq_], 1.74 (m, 1 H, 4′′-H), 1.81 [broad d, *J* = 12.8 Hz, 2 H, 10′(13′)-H_ax_], 2.04 (d, *J* = 7.1 Hz, 2 H, 2-H), 2.06–2.17 [complex signal, 8 H, 6′(12′)-H_2_, 10′(13′)-H_eq_, 2′′(6′′)-H_ax_], 2.19 (d, *^2^J_HF_* = 6.5 Hz, 2 H, 8′-H_2_), 2.94 [broad d, *J* = 12.1 Hz, 2 H, 2′′(6′′)-H_eq_], 3.23 [broad s, 2 H, 5′(11′)-H], 3.57 (s, 2 H, CH_2_-C_6_H_5_), 7.06–7.10 [complex signal, 4 H, 1′(4′)-H, 2′(3′)-H], 7.28 (m, 1 H, 4′′′-H), 7.31–7.36 [complex signal, 4 H, 2′′′(6′′′)-H, 3′′′(5′′′)-H]. ^13^C-NMR (100.6 MHz, CD_3_OD) δ: 32.2 [CH_2_, C3′′(5′′)], 34.5 (CH, C4′′), 39.3 [CH_2_, C6′(12′)], 41.0 [d, *^3^J_CF_* = 13.1 Hz, CH, C5′(11′)], 41.4 [d, *^2^J_CF_* = 20.1 Hz, CH_2_, C10′(13′)], 44.5 (CH_2_, C2), 46.9 (d, *^2^J_CF_* = 18.3 Hz, CH_2_, C8′), 54.4 [CH_2_, C2′′(6′′)], 58.8 (d, *^3^J_CF_* = 11.2 Hz, C, C7′), 64.1 (CH_2_, CH_2_-C_6_H_5_), 94.7 (d, *^1^J_CF_* = 177.1 Hz, C, C9′), 127.9 [CH, C2′(3′)], 128.7 (CH, C4′′′), 129.2 [CH, C1′(4′)], 129.4 [CH, C3′′′(5′′′)], 131.0 [CH, C2′′′(6′′′)], 137.5 (C, C1′′′), 146.3 [C, C4a(11a)], 174.1 (C, CONH). Anal. Calcd for C_29_H_35_FN_2_O: C 77.99, H 7.90, N 6.27; Calcd for C_29_H_35_FN_2_O · 0.6 H_2_O: C 76.15, H 7.98, N 6.12. Found: C 75.99, H 7.81, N 6.06. HRMS: Calcd for [C_29_H_35_FN_2_O + H]^+^: 447.2806, found: 447.2804.

#### 3.1.16. Synthesis of 2-(1-(4-Acetylphenyl)piperidin-4-yl)acetic Acid, **9a**

To a solution of 2-(piperidin-4-yl)acetic acid hydrochloride (200 mg, 1.11 mmol), **8**, and 4′-fluoroacetophenone (163 µL, 185 mg, 1.34 mmol) in DMSO (3 mL) was added K_2_CO_3_ (612 mg, 4.44 mmol) and the mixture was stirred at 100 °C overnight. Water was added (20 mL) followed by HCl 1 M until pH = 4. The mixture was extracted with EtOAc (20 mL). The organic layer was washed again with water at pH = 4 (20 mL). Then, the organic layer was dried over anh. Na_2_SO_4_, filtered, and solvents were concentrated *in vacuo* to afford **9a** as an orangish solid that was used as such without further purification (132 mg, 45% yield) [33]. ^1^H-NMR (400 MHz, CD_3_OD) δ: 1.34 (qd, *J* = 12.3 Hz, *J’* = 4.0 Hz, 2 H), 1.86 (m, 2 H), 2.01 (m, 1 H), 2.26 (d, *J* = 7.1 Hz, 2 H), 2.49 (s, 3 H), 2.91 (td, *J* = 12.8 Hz, *J’* = 2.6 Hz, 2 H), 3.98 (m, 2 H), 6.94 (d, *J* = 9.1 Hz, 2 H), 7.86 (d, *J* = 9.0 Hz, 2 H).

#### 3.1.17. Synthesis of 2-(1-(4-Cyanophenyl)piperidin-4-yl)acetic Acid, **9b**

To a solution of 2-(piperidin-4-yl)acetic acid hydrochloride (800 mg, 4.45 mmol), **8**, and 4-fluorobenzonitrile (647 mg, 5.34 mmol) in DMSO (15 mL) was added K_2_CO_3_ (2.45 g, 17.76 mmol) and the mixture was stirred at 100 °C overnight. Water was added (50 mL) followed by HCl 1 M until pH = 4. The mixture was extracted with EtOAc (30 mL). The organic layer was washed again with water at pH = 4 (50 mL). Then, the organic layer was dried over anh. Na_2_SO_4_, filtered, and solvents were concentrated *in vacuo* to afford **9b** as a beige solid that was used as such without further purification (708 mg, 65% yield) [34]. ^1^H-NMR (400 MHz, CD_3_OD) δ: 1.33 (qd, *J* = 12.7 Hz, *J’* = 4.0 Hz, 2 H), 1.86 (m, 2 H), 2.00 (m, 1 H), 2.26 (d, *J* = 7.1 Hz, 2 H), 2.89 (td, *J* = 12.7 Hz, *J’* = 2.6 Hz, 2 H), 3.94 (m, 2 H), 6.98 (d, *J* = 9.1 Hz, 2 H), 7.48 (d, *J* = 9.0 Hz, 2 H).

#### 3.1.18. Synthesis of 2-(1-(4-(t-Butoxycarbonyl)phenyl)piperidin-4-yl)acetic Acid, **9c**

To a solution of 2-(piperidin-4-yl)acetic acid hydrochloride (369 mg, 2.06 mmol), **8**, and *t*-butyl 4-fluorobenzoate (443 mg, 2.26 mmol) in DMSO (8 mL) was added K_2_CO_3_ (1.14 g, 8.22 mmol) and the mixture was stirred at 130 °C for 48 h. Water was added (20 mL) followed by HCl 1 M until pH = 5. The mixture was extracted with EtOAc (20 mL). The organic layer was washed again with water at pH = 5 (20 mL). Then, the organic layer was dried over anh. Na_2_SO_4_, filtered, and solvents were concentrated under vacuum. The resulting crude was purified by column chromatography in silica gel (SiO_2_, Hexane/EtOAc mixtures) and gave **9c** as a white solid (255 mg, 39% yield). ^1^H-NMR (400 MHz, CD_3_OD) δ: 1.40 (qd, *J* = 12.6 Hz, *J’* = 4.0 Hz, 2 H), 1.57 (s, 9 H), 1.87 (d, *J* = 13.1 Hz, 2 H), 2.02 (m, 1 H), 2.33 (d, *J* = 7.0 Hz, 2 H), 2.86 (td, *J* = 12.4 Hz, *J’* = 2.6 Hz, 2 H), 3.84 (d, *J* = 12.5 Hz, 2 H), 6.85 (d, *J* = 8.9 Hz, 2 H), 7.86 (d, *J* = 9.1 Hz, 2 H).

#### 3.1.19. Synthesis of 2-(1-(2-Bromo-4-(methoxycarbonyl)phenyl)piperidin-4-yl)acetic Acid, **9d**

To a solution of 2-(piperidin-4-yl)acetic acid hydrochloride (200 mg, 1.11 mmol), **8**, and methyl 3-bromo-4-fluorobenzoate (311 mg, 1.34 mmol) in DMF (3 mL) was added K_2_CO_3_ (612 mg, 4.44 mmol) and the mixture was stirred at 100 °C for 24 h. Water was added (20 mL) followed by HCl 1 M until pH = 4. The mixture was extracted with EtOAc (20 mL). The organic layer was washed again with water at pH = 4 (20 mL). Then, the organic layer was dried over anh. Na_2_SO_4_, filtered, and solvents were concentrated under vacuum. The resulting crude was purified by column chromatography in silica gel (SiO_2_, DCM/MeOH mixtures) and gave **9d** as a white solid (200 mg, 50% yield). ^1^H-NMR (400 MHz, CDCl_3_) δ: 1.51 (m, 2 H), 1.85 (d, *J* = 13.0 Hz, 3 H), 2.32 (d, *J* = 6.9 Hz, 2 H), 2.71 (t, *J* = 11.7 Hz, 2 H), 3.46 (d, *J* = 11.5 Hz, 2 H), 3.88 (s, 3 H), 7.01 (d, *J* = 8.5 Hz, 1 H), 7.91 (dd, *J* = 8.4 Hz, *J’* = 2.0 Hz, 1 H), 8.21 (d, *J* = 2.0 Hz, 1 H).

#### 3.1.20. Synthesis of 2-(1-(2-Cyclopropyl-4-(methoxycarbonyl)phenyl)piperidin-4-yl)acetic Acid, **9e**

A suspension of 2-(1-(2-bromo-4-(methoxycarbonyl)phenyl)piperidin-4-yl)acetic acid (200 mg, 0.56 mmol), **9d**, cyclopropylboronic acid (96 mg, 1.12 mmol) and K_3_PO_4_ (360 mg, 1.70 mmol) in dioxane (5 mL) was degassed bubbling with N_2_ for 10 min. Then, tetrakis(triphenylphosphine)palladium(0) (65 mg, 0.06 mmol) was added and the mixture was heated at 100 °C and stirred for 24 h. Water was added (20 mL) followed by HCl 1 M until pH = 4. The mixture was extracted with EtOAc (20 mL). The aqueous layer was extracted again with EtOAc (20 mL). Both organic layers were dried over anh. Na_2_SO_4_, filtered, and solvents were concentrated under vacuum. The resulting crude was purified by column chromatography in silica gel (SiO_2_, DCM/MeOH mixtures) and gave **9e** as a green solid. ^1^H-NMR (400 MHz, CDCl_3_) δ: 0.79 [m, 2 H, 2′′′(3′′′)-H_ax_], 1.02 [m, 2 H, 2′′′(3′′′)-H_eq_], 1.52 [qd, *J* = 12.0 Hz, *J’* = 3.8 Hz, 2 H, 3′(5′)-H_ax_], 1.89 [m, 2 H, 3′(5′)-H_ax_], 1.99 (m, 1 H, 4′-H), 2.13 (m, 1 H, 1′′′-H), 2.39 [d, *J* = 7.0 Hz, 2 H, 3′(5′)-H_eq_], 2.73 [td, *J* = 12.0 Hz, *J’* = 2.2 Hz, 2 H, 2′(6′)-H_ax_], 3.45 [d, *J* = 11.9 Hz, 2 H, 2′(6′)-H_eq_], 3.86 (s, 3 H, CH_3_), 6.97 (d, *J* = 8.4 Hz, 1 H, 6′′-H), 7.45 (d, *J* = 2.1 Hz, 1 H, 3′′-H), 7.78 (dd, *J* = 8.4 Hz, *J’* = 2.1 Hz, 1 H, 5′′-H).

#### 3.1.21. Synthesis of 2-(1-(4-Acetylphenyl)piperidin-4-yl)-*N*-(9-fluoro-5,6,8,9,10,11-hexahydro-7*H*-5,9:7,11-dimethanobenzo[9]annulen-7-yl)acetamide, **10a**

To a solution of 9-fluoro-5,6,8,9,10,11-hexahydro-7*H*-5,9:7,11-dimethanobenzo[9]annulen-7-amine (50 mg, 0.22 mmol), **2d**, in DMF (1 mL), 2-(1-(4-acetylphenyl)piperidin-4-yl)acetic acid (68 mg, 0.26 mmol), **9a**, HATU (123 mg, 0.32 mmol), and DIPEA (85 mg, 0.66 mmol) were added. The mixture was stirred at room temperature for 24 h. Solvent was concentrated *in vacuo* and EtOAc (10 mL) was added. The mixture was washed with NaHCO_3_ sat. (2 x 20 mL). The organic phase was dried over anh. Na_2_SO_4_, filtered, and concentrated under vacuum. The resulting crude was purified by column chromatography in silica gel (SiO_2_, Hexane/EtOAc mixtures) and gave **10a** as an off-white solid (33 mg, 32% yield), mp: 229–230 °C. IR (ATR) *v*: 3318, 2931, 2854, 1659, 1644, 1605, 1551, 1514, 1443, 1380, 1359, 1282, 1233, 1180, 1169, 1080, 1011, 998, 960, 865, 835, 818, 767, 632, 603, 593, 569 cm^−1^. ^1^H-NMR (400 MHz, CDCl_3_) δ: 1.30 [m, 2H, 3′′(5′′)-H_ax_], 1.79 [dm, *J* = 12.7 Hz, 2 H, 3′′(5′′)-H_eq_], 1.93 [m, 2 H, 10′(13′)-H_ax_], 1.97–2.06 [complex signal, 5 H, 2-H_2_, 6′(12′)-H_ax_, 4′′-H], 2.14–2.26 [complex signal, 4 H, 6′(12′)-H_eq_, 10′(13′)-H_eq_], 2.29 (d, *^2^J_HF_* = 6.4 Hz, 2 H, 8′-H_2_), 2.50 (s, 3 H, CH_3_), 2.88 [td, *J* = 12.7 Hz, *J’* = 2.6 Hz, 2 H, 2′′(6′′)-H_ax_], 3.24 [m, 2 H, 5′(11′)-H], 3.87 [m, 2 H, 2′′(6′′)-H_eq_], 5.30 (broad s, 1 H, NH), 6.84 [d, *J* = 9.0 Hz, 2 H, 2′′′(6′′′)-H], 7.08 [m, 2 H, 1′(4′)-H], 7.13 [m, 2 H, 2′(3′)-H], 7.84 [d, *J* = 9.0 Hz, 2 H, 3′′′(5′′′)-H]. ^13^C-NMR (100.6 MHz, CDCl_3_) δ: 26.2 (CH_3_, COCH_3_), 31.4 [CH_2_, C3′′(5′′)], 33.6 (CH, C4′′), 38.7 [CH_2_, C6′(12′)], 39.6 [d, *^3^J_CF_* = 13.3 Hz, CH, C5′(11′)], 40.2 [d, *^2^J_CF_* = 20.1 Hz, CH_2_, C10′(13′)], 44.6 (CH_2_, C2), 46.1 (d, *^2^J_CF_* = 18.4 Hz, CH_2_, C8′), 48.0 [CH_2_, C2′′(6′′)], 58.0 (d, *^3^J_CF_* = 11.4 Hz, C, C7′), 94.2 (d, *^1^J_CF_* = 177.6 Hz, C, C9′), 113.6 [CH, C2′′′(6′′′)], 127.1 [CH, C2′(3′)], 128.3 [CH, C1′(4′)], 130.6 [CH, C3′′′(5′′′)], 144.8 [C, C4a(11a)], 154.1 (C, C1′′′), 170.8 (C, CONH), 196.6 (C, COCH_3_). The signal from C4′′′ was not observed. Anal. Calcd for C_30_H_35_FN_2_O_2_: C 75.92, H 7.43, N 5.90; Calcd for C_30_H_35_FN_2_O_2_ · 0.25 H_2_O: C 75.21, H 7.47, N 5.85. Found: 75.34, H 7.31, N 5.69. HRMS: Calcd for [C_30_H_35_FN_2_O_2_ + H]^+^: 475.2755, found: 475.2763.

#### 3.1.22. Synthesis of 2-(1-(4-Cyanophenyl)piperidin-4-yl)-*N*-(9-fluoro-5,6,8,9,10,11-hexahydro-7*H*-5,9:7,11-dimethanobenzo[9]annulen-7-yl)acetamide, **10b**

To a solution of 9-fluoro-5,6,8,9,10,11-hexahydro-7*H*-5,9:7,11-dimethanobenzo[9]annulen-7-amine (200 mg, 0.86 mmol), **2d**, in DMF (4 mL), 2-(1-(4-cyanophenyl)piperidin-4-yl)acetic acid (252 mg, 1.03 mmol), **9b**, HATU (490 mg, 1.29 mmol), and DIPEA (333 mg, 2.58 mmol) were added. The mixture was stirred at room temperature for 24 h. Solvent was concentrated in vacuo and EtOAc (20 mL) was added. The mixture was washed with brine (2 × 40 mL). The organic phase was dried over anh. Na_2_SO_4_, filtered, and concentrated under vacuum. The resulting crude was purified by column chromatography in silica gel (SiO_2_, Hexane/EtOAc mixtures) and gave **10b** as an off-white solid (23 mg, 6% yield), mp: 180–181 °C. IR (ATR) *v*: 3317, 2926, 2857, 2212, 1645, 1603, 1515, 1445, 1359, 1306, 1239, 1177, 1112, 1089, 1010, 863, 819, 762, 732, 680, 645, 569, 560 cm^−1^. ^1^H-NMR (400 MHz, CDCl_3_) δ: 1.29 [m, 2 H, 3′′(5′′)-H_ax_], 1.80 [broad d, *J* = 10.3 Hz, 2 H, 3′′(5′′)-H_eq_], 1.93 [m, 2 H, 10′(13′)-H_ax_], 1.97–2.08 [complex signal, 5 H, 2-H_2_, 6′(12′)-H_ax_, 4′′-H], 2.11–2.25 [complex signal, 4 H, 6′(12′)-H_eq_, 10′(13′)-H_eq_], 2.28 (d, *^2^J_HF_* = 6.3 Hz, 2 H, 8′-H_2_), 2.87 [td, *J* = 12.7 Hz, *J’* = 2.6 Hz, 2 H, 2′′(6′′)-H_ax_], 3.23 [m, 2 H, 5′(11′)-H], 3.82 [broad dt, *J* = 12.9 Hz, *J’* =2.5 Hz, 2 H, 2′′(6′′)-H_eq_], 5.33 (broad s, 1 H, NH), 6.83 [d, *J* = 9.0 Hz, 2 H, 2′′′(6′′′)-H], 7.08 [m, 2 H, 1′(4′)-H], 7.12 [m, 2 H, 2′(3′)-H], 7.45 [d, *J* = 9.0 Hz, 2 H, 3′′′(5′′′)-H]. ^13^C-NMR (100.6 MHz, CDCl_3_) δ: 31.3 [CH_2_, C3′′(5′′)], 33.5 (CH, C4′′), 38.7 [CH_2_, C6′(12′)], 39.6 [d, *^3^J_CF_* = 13.2 Hz, CH, C5′(11′)], 40.1 [d, *^2^J_CF_* = 20.2 Hz, CH_2_, C10′(13′)], 44.4 (CH_2_, C2), 46.0 (d, *^2^J_CF_* = 18.4 Hz, CH_2_, C8′), 47.9 [CH_2_, C2′′(6′′)], 58.0 (d, *^3^J_CF_* = 11.4 Hz, C, C7′), 94.2 (d, *^1^J_CF_* = 177.6 Hz, C, C9′), 99.5 (C, CN), 114.4 [CH, C2′′′(6′′′)], 120.3 (C, C4′′′), 127.1 [CH, C2′(3′)], 128.3 [CH, C1′(4′)], 133.6 [CH, C3′′′(5′′′)], 144.7 [C, C4a(11a)], 153.3 (C, C1′′′), 170.7 (C, CONH). Anal. Calcd for C_29_H_32_FN_3_O: C 76.12, H 7.05, N 9.18; Calcd for C_29_H_32_FN_3_O · 0.5 CH_2_Cl_2_: C 70.86, H 6.65, N 8.40. Found: C 70.78, H 6.67, N 8.17. HRMS: Calcd for [C_29_H_32_FN_3_O + H]^+^: 458.2602, found: 458.2593.

#### 3.1.23. Synthesis of 4-(4-(2-((9-Fluoro-5,6,8,9,10,11-hexahydro-7*H*-5,9:7,11-dimethanobenzo[9]annulen-7-yl)amino)-2-oxoethyl)piperidin-1-yl)benzoic Acid, **10c**

To a solution of 9-fluoro-5,6,8,9,10,11-hexahydro-7*H*-5,9:7,11-dimethanobenzo[9]annulen-7-amine (82 mg, 0.36 mmol), **2d**, in DMF (2 mL) were added 2-(1-(4-(*t*-butoxycarbonyl)phenyl)piperidin-4-yl)acetic acid (125 mg, 0.39 mmol), **9c**, HATU (203 mg, 0.53 mmol), and DIPEA (92 mg, 0.71 mmol). The mixture was stirred at room temperature for 24 h. Solvent was concentrated in vacuo and EtOAc (15 mL) was added. The mixture was washed with brine (2 × 30 mL). The organic phase was dried over anh. Na_2_SO_4_, filtered, and concentrated under vacuum. The resulting crude was purified by column chromatography in silica gel (SiO_2_, Hexane/EtOAc mixtures). Fractions containing the desired product were collected and concentrated in vacuo. Then, HCl 4 M in dioxane (2 mL) with some drops of water were added to the solid and the mixture was stirred at RT overnight. EtOAc (10 mL) was added, and the mixture was washed with water acidified at pH = 4 with HCl 2 M (2 x 20 mL). The organic layer was dried over anh. Na_2_SO_4_, filtered, and solvents were concentrated under vacuum. The resulting crude was purified by column chromatography in silica gel (SiO_2_, DCM/MeOH mixtures) and gave **10c** as an off-white solid (40 mg, 24% yield), mp: 267–268 °C. IR (ATR) *v*: 2923, 2853, 1665, 1634, 1601, 1519, 1431, 1418, 1318, 1283, 1211, 1189, 1089, 993, 827, 767, 697, 632, 553 cm^−1^. ^1^H-NMR (400 MHz, CD_3_OD) δ: 1.34 [qd, *J* = 12.6 Hz, *J’* = 4.1 Hz, 2 H, 3′(5′)-H_ax_], 1.76 [broad d, *J* = 12.1 Hz, 2 H, 3′(5′)-H_eq_], 1.82 [broad d, *J* = 10.4 Hz, 2 H, 10′′′(13′′′)-H_ax_], 1.95 (m, 1 H, 4′-H), 2.05–2.13 [complex signal, 4 H, 1′′-H, 6′′′(12′′′)-H_ax_], 2.13–2.19 [complex signal, 4 H, 6′′′(12′′′)-H_eq_, 10′′′(13′′′)-H_ax_], 2.21 (d, *^2^J_HF_* = 6.4 Hz, 2 H, 8′′′-H), 2.84 [td, *J* = 12.7 Hz, *J’* = 2.6 Hz, 2 H, 2′(6′)-H_ax_], 3.24 (broad s, 2 H, 5′′′(11′′′)-H], 3.92 [broad d, *J* = 13.3 Hz, 2 H, 2′(6′)-H_eq_], 6.93 [d, *J* = 9.1 Hz, 2 H, 3(5)-H], 7.07–7.13 [complex signal, 4 H, 1′′′(4′′′)-H, 2′′′(3′′′)-H], 7.85 [d, *J* = 9.1 Hz, 2 H, 2(6)-H]. ^13^C-NMR (100.6 MHz, CD_3_OD) δ: 32.4 [CH_2_, C3′(5′)], 35.1 (CH, C4′), 39.3 [CH_2_, C6′′′(12′′′)], 41.0 [d, *^3^J_CF_* = 13.1 Hz, CH, C5′′′(11′′′)], 41.4 [d, *^2^J_CF_* = 20.2 Hz, CH_2_, C10′′′(13′′′)], 44.7 (CH_2_, C1′′), 46.9 (d, *^2^J_CF_* = 18.4 Hz, CH_2_, C8′′′), 49.1 [CH_2_, C2′′(6′′)], 58.8 (d, *^3^J_CF_* = 11.4 Hz, C, C7′′′), 94.8 (d, *^1^J_CF_* = 177.1 Hz, C, C9′′′), 114.9 [CH, C3(5)], 120.1 (C, C1), 128.0 [CH, C2′′′(3′′′)], 129.2 [CH, C1′′′(4′′′)], 132.5 [CH, C2(6)], 146.3 [C, C4a’’(11a’’)], 155.9 (C, C4), 170.4 (C, CONH), 174.1 (C, CO_2_H). Anal. Calcd for C_29_H_33_FN_2_O_3_: C 73.09, H 6.98, N 5.88. Found: C 72.64, H 7.16, N 5.39. HRMS: Calcd for [C_29_H_33_FN_2_O_3_−H]^−^: 475.2402, found: 475.2400.

#### 3.1.24. Synthesis of 3-Cyclopropyl-4-(4-(2-((9-fluoro-5,6,8,9,10,11-hexahydro-7*H*-5,9:7,11-dimethanobenzo[9]annulen-7-yl)amino)-2-oxoethyl)piperidin-1-yl)benzoic Acid, **10d**

To a solution of 9-fluoro-5,6,8,9,10,11-hexahydro-7*H*-5,9:7,11-dimethanobenzo[9]annulen-7-amine (100 mg, 0.45 mmol), **2d**, in DMF (2 mL) were added 2-(1-(2-cyclopropyl-4-(methoxycarbonyl)phenyl)piperidin-4-yl)acetic acid (125 mg, 0.39 mmol), **9e**, HATU (203 mg, 0.53 mmol), and DIPEA (92 mg, 0.71 mmol). The mixture was stirred at room temperature for 24 h. Solvent was concentrated in vacuo and EtOAc (10 mL) was added. The mixture was washed with NaHCO_3_ sat. (2 × 20 mL). The organic phase was dried over anh. Na_2_SO_4_, filtered, and concentrated under vacuum. The resulting crude was purified by column chromatography in silica gel (SiO_2_, Hexane/EtOAc mixtures). Fractions containing the desired product were collected and concentrated in vacuo. MeOH (1 mL) and KOH (116 mg, 2.07 mmol) were added, and the mixture was stirred at 50 °C for 4 h. Amberlite^®^ 120 H+ was added until pH = 4 and the mixture was filtered, using MeOH as an eluting agent. Solvents were concentrated in vacuo to afford a white solid that was purified by column chromatography in silica gel (SiO_2_, DCM/MeOH mixtures) and gave **10d** as a reddish solid (16 mg, 7% yield), mp: 190–191 °C. IR (ATR) *v*: 2920, 2854, 1645, 1602, 1539, 1495, 1442, 1382, 1359, 1303, 1228, 1180, 1116, 1089, 1011, 937, 864, 758, 715, 641, 569 cm^−1^. ^1^H-NMR (400 MHz, CD_3_OD) δ: 0.73 [m, 2 H, 2′(3′)-H_ax_], 1.02 [m, 2 H, 2′(3′)-H_eq_], 1.47 [qd, *J* = 12.0 Hz, *J’* = 3.8 Hz, 2 H, 3′′(5′′)-H_ax_], 1.76–1.86 [complex signal, 4 H, 3′′(5′′)-H_eq_, 10′′′′(13′′′′)-H_ax_], 1.90 (m, 1 H, 4′′-H), 2.06–2.21 [complex signal, 9 H, 1′-H, 1′′′-H, 6′′′′(12′′′′)-H_2_, 10′′′′(13′′′′)-H_eq_], 2.22 (d, *^2^J_HF_* = 6.4 Hz, 2 H, 8′′′′-H), 2.71 [td, *J* = 11.9 Hz, *J’* = 2.2 Hz, 2 H, 2′′(6′′)-H_ax_], 3.25 [broad t, *J* = 5.7 Hz, 2 H, 5′′′′(11′′′′)-H], 3.43 [broad d, *J* = 12.1 Hz, 2 H, 2′′(6′′)-H_eq_], 7.03 (d, *J* = 8.4 Hz, 1 H, 5-H), 7.10 [broad signal, 4 H, 1′′′′(4′′′′)-H, 2′′′′(3′′′′)-H], 7.44 (d, *J* = 2.0 Hz, 1 H, 2-H), 7.75 (dd, *J* = 8.4 Hz, *J’* = 2.1 Hz, 1 H, 6-H). ^13^C-NMR (100.6 MHz, CD_3_OD) δ: 9.9 [CH_2_, C2′(3′)], 12.2 (CH, C1′), 33.6 [CH_2_, C3′′(5′′)], 35.0 (CH, C4′′), 39.4 [CH_2_, C6′′′′(12′′′′)], 41.0 [d, *^3^J_CF_* = 13.2 Hz, CH, C5′′′′(11′′′′)], 41.4 [d, *^2^J_CF_* = 20.1 Hz, CH_2_, C10′′′′(13′′′′)], 44.9 (CH_2_, C1′′′), 46.9 (d, *^2^J_CF_* = 18.3 Hz, CH_2_, C8′′′′), 53.3 [CH_2_, C2′′(6′′)], 58.8 (d, *^3^J_CF_* = 11.3 Hz, C, C7′′′′), 94.8 (d, *^1^J_CF_* = 177.2 Hz, C, C9′′′′), 119.1 (CH, C5), 125.6 (C, C1), 126.4 (CH, C2), 128.0 [CH, C2′′′′(3′′′′)], 129.0 (CH, C6), 129.2 [CH, C1′′′′(4′′′′)], 138.1 (C, C3), 146.3 [C, C4a’’’(11a’’’)], 158.3 (C, C4), 170.5 (C, CONH), 174.3 (C, CO_2_H). HRMS: Calcd for [C_32_H_37_FN_2_O_3_ + H]^+^: 517.2861, found: 517.2847.

#### 3.1.25. Synthesis of 4-(4-(2-(9-Chloro-5,6,8,9,10,11-hexahydro-7*H*-5,9:7,11-dimethanobenzo[9]annulen-7-yl)ureido)piperidin-1-yl)benzoic Acid, **10e**

To a solution of 9-chloro-5,6,8,9,10,11-hexahydro-7*H*-5,9:7,11-dimethanobenzo[9]annulen-7-amine (88 mg, 0.36 mmol), **2e**, in DMF (2 mL), 2-(1-(4-(*t*-butoxycarbonyl)phenyl)piperidin-4-yl)acetic acid (125 mg, 0.39 mmol), **9c**, HATU (203 mg, 0.53 mmol), and DIPEA (124 µL, 92 mg, 0.71 mmol) were added. The mixture was stirred at room temperature overnight. EtOAc (15 mL) was added, and the mixture was washed with brine (2 x 30 mL). The organic layer was dried over anh. Na_2_SO_4_, filtered, and solvents were concentrated. The resulting crude was purified by column chromatography in silica gel (using as eluent mixtures of EtOAc in hexane from 0% to 25%). Fractions containing the desired product were collected and concentrated in vacuo. HCl 4 M in dioxane (2 mL) with some drops of water were added and the mixture was stirred at room temperature overnight. EtOAc (10 mL) was added, and the mixture was washed with water acidified at pH = 4 with HCl 2 M (2 × 20 mL). The organic layer was dried over anh. Na_2_SO_4_, filtered, and solvents were concentrated in vacuo. The crude was purified by column chromatography in silica gel (using as eluent mixtures of MeOH in DCM from 0% to 4%). Fractions containing the desired product were collected and concentrated *in vacuo* to afford **10e** as a pink solid (23 mg, 13% yield), mp: 242–243 °C. IR (ATR) *v*: 2920, 2855, 1664, 1638, 1600, 1518, 1415, 1391, 1357, 1283, 1230, 1184, 1110, 1082, 978, 947, 931, 900, 830, 800, 773, 762, 697, 645 cm^−1^. ^1^H-NMR (400 MHz, CD_3_OD) δ: 1.35 [m, 2 H, 3′(5′)-H_ax_], 1.77 [broad d, *J* = 12.9 Hz, 2 H, 3′(5′)-H_eq_], 1.95 (m, 1 H, 4′-H), 2.02–2.11 [complex signal, 6 H, 1′′-H, 6′′′(12′′′)-H_ax_, 10′′′(13′′′)-H_ax_], 2.23 [m, 2 H, 6′′′(12′′′)-H_eq_], 2.40 [m, 2 H, 10′′′(13′′′)-H_eq_], 2.49 (s, 2 H, 8′′′-H), 2.85 [td, *J* = 12.6 Hz, *J’* = 2.6 Hz, 2 H, 2′(6′)-H_ax_], 3.19 [broad t, *J* = 6.5 Hz, 2 H, 5′′′(11′′′)-H], 3.92 [broad d, *J* = 13.4 Hz, 2 H, 2′(6′)-H_eq_], 6.93 [d, *J* = 9.1 Hz, 2 H, 3(5)-H], 7.06–7.12 [complex signal, 4 H, 1′′′(4′′′)-H, 2′′′(3′′′)-H], 7.85 [d, *J* = 9.0 Hz, 2 H, 2(6)-H]. ^13^C-NMR (101 MHz, CD_3_OD) δ: 32.4 [CH_2_, C3′(5′)], 35.1 (CH, C4′), 39.0 [CH_2_, C6′′′(12′′′)], 42.6 [CH, C5′′′(11′′′)], 44.7 (CH_2_, C1′′), 45.9 [CH_2_, C10′′′(13′′′)], 49.1 [signal overlapped, CH_2_, C2′(6′)], 51.0 (CH_2_, C8′′′), 57.6 (C, C7′′′), 70.3 (C, C9′′′), 114.9 [CH, C3(5)], 120.1 (C, C1), 128.0 [CH, C2′′′(3′′′)], 129.1 [CH, C1′′′(4′′′)], 132.5 [CH, C2(6)], 146.2 [C, C4a’’’(11a’’’)], 155.9 (C, C4), 170.4 (C, CONH), 174.1 (C, CO_2_H). Anal. Calcd for C_29_H_33_ClN_2_O_3_: C 70.65, H 6.75, N 5.68; Calcd for C_29_H_33_ClN_2_O_3_ · ¾ H_2_O: 68.76, H 6.86, N 5.53. Found: C 68.77, H 6.66, N 5.18. HRMS: Calcd for [C_29_H_33_ClN_2_O_3_−H]^−^: 491.2107, found: 491.2106.

### 3.2. Microsomal Stability

The human and murine pooled microsomes employed were purchased from Tebu-Xenotech (Barcelona, Spain). The compound was incubated at 37 °C with the microsomes in a 50 mM phosphate buffer (pH = 7.4) containing 3 mM MgCl_2_, 1 mM NADP, 10 mM glucose-6-phosphate and 1 U/mL glucose-6-phosphate-dehydrogenase. Samples (75 µL) were taken from each well at 0, 10, 20, 40 and 60 min and transferred to a plate containing 4 °C 75 µL acetonitrile and 30 µL of 0.5% formic acid in water were added for improving the chromatographic conditions. The plate was centrifuged (46000 g, 30 min) and supernatants were taken and analyzed in a UPLC-MS/MS (Xevo-TQD, Waters) by employing a BEH C18 column and an isocratic gradient of 0.1% formic acid in water: 0.1% formic acid acetonitrile (60:40). The metabolic stability of the compounds was calculated from the logarithm of the remaining compounds at each of the time points studied [19].

### 3.3. Cytotoxicity Assay in SH-SY5Y Cells

Cytotoxicity was evaluated in the human neuroblastoma SH-SY5Y cell line (ATCC Number: CRL-2266). Cells were cultured in minimum essential medium/HAM’s-F12 (1:1, *v*/*v*) medium, supplemented with non-essential amino acids, L-glutamine 1 mM, gentamycin 50 µM and 10% heat inactivated fetal bovine serum (FBS). For experiments, the cells were seeded at 3 × 10^5^ cells/mL (100 µL/well) in 96-well plates. After 24 h, either **10c** or the reference compound, TPPU, were added concentrated to the wells to yield a range of concentrations up to 100 µM. DMSO 0.1% was used as vehicle. Treated cultures were returned to the incubator for a 24 h test exposure. One hour before termination, cell death was measured by PI staining. Briefly, PI (Molecular Probes) at the final concentration of 7.5 µg/mL was added to the cells and incubated for 1 h. PI enters into cells with damaged membrane and binds to DNA yielding intense red fluorescence. Fluorescence was measured in a SAFAS FLX-Xenius microplate reader (Monaco) at 530 nm excitation and 645 nm emission. Percentage of cell death for a given fluorescence value (Ft) was calculated according to the values given by Triton X100 killed cells (Fmax; 100% cell death) and vehicle treated cells (Fmin; 0% cell death) [% = ((Ft-Fmin)/(Fmax-Fmin)) × 100]. Each treatment was assayed in 3–4 wells per experiment and the experiment was repeated 3 times in cultures of different cell passages [35]. Data were analyzed by ANOVA.

### 3.4. Inflammatory Assay in the Microglial BV2 Cell Line

Inflammatory changes were determined by the measure of nitric oxide generation, as the endpoint of iNOS activation, in microglial BV2 cells. BV2 cells (HyperCLDB, Banca Biologica e Cell Factory, ICLC ATL 03001) were grown in RPMI 1640 medium, supplemented with L-glutamine 2 mM, gentamycin 50 µM and 10% FBS. For experiments, cells were seeded in 96-well plates at 2 × 10^5^ cells/mL, 100 µL/well. After 24 h, the medium was replaced with fresh culture medium without FBS containing the vehicle or the anti-inflammatory agent **10c** at 50 µM or 100 µM. After 1 h of incubation, the cells were treated with the pro-inflammatory chemical LPS (0.1 μg/mL or 1 μg/mL; strain E. coli 026:B26, Sigma-Aldrich, L-2654, batch #120M4028) and further incubated for 24 h. TPPU at 100 µM was also assayed as a reference anti-inflammatory compound. DMSO 0.1% was used as vehicle. Cells were maintained at 37 °C in a humidified incubator with 5% CO_2_ throughout the procedures. Nitric oxide released to the culture medium was measured by the colorimetric Griess reaction [36] that detects nitrite (NO_2_^–^), a stable reaction product of nitric oxide and molecular oxygen. Briefly, 50 µL of conditioned medium were incubated with 50 µL of Griess reagent for 10 min at room temperature. Optical density was measured at 540 nm. Nitrite concentration was determined from a sodium nitrite standard curve. Results were normalized by cell protein content in the well and expressed as a percentage of the average maximal values given by the pro-inflammatory agent (LPS 1 µg/mL). Each treatment was performed in 3–5 wells and the whole experiment was repeated 3–4 times in cultures of different cell passage. Data were analyzed by ANOVA followed by Tukey’s multiple comparisons test.

## 4. Conclusions

Starting from a previous series of ureas with high potency as sEHI but poor microsomal stability, several amides were synthesized and fully characterized. Amide **10c**, endowed with excellent potency, tolerable microsomal stability, and no cytotoxicity, emerged as a promising compound. **10c** was highly effective in the inhibition of nitric oxide generated in microglial BV2 cells activated by LPS. Furthermore, it showed higher effectiveness than TPPU, the reference sEHI, that was reported to have anti-inflammatory properties in the brain of mouse models of Alzheimer’s disease [37,38]. The results indicate **10c** as an excellent candidate for further in vitro characterization as an anti-inflammatory and neuroprotective agent although its limited microsomal stability may prevent in vivo development. Overall, the results emphasize the significance of sEH as a druggable target in therapies involving inflammatory processes.

## 5. Patents

A PCT patent application has been filed. See PCT WO2019/243414A1 (priority data 20 June 2018).

## Figures and Tables

**Table 1:** IC_50_ in human and murine sEH and microsomal stability values of **1**, **6a–h** and **10a–e**.

Cmpd #	Human sEH	Murine sEH	Microsomal Stability ^1^	
			Human	Mouse
	IC_50_ (nM) ^2^		% Remaining at 1 h	
**1**	3.1	6.0	1.0	1.0
**6a**	34.5	0.4	0.3	0.0
**6b**	30.2	0.4	0.0	0.0
**6c**	1.6	0.4	5.5	5.1
**6d**	1.0	0.4	7.0	1.4
**6e**	2.8	0.5	0.6	0.2
**6f**	2.4	0.4	1.3	0.5
**6g**	37.5	3.3	0.1	0.1
**6h**	21.4	12.9	0.3	0.1
**10a**	0.6	0.7	0.1	0.1
**10b**	3.4	0.4	0.0	0.1
**10c**	0.4	0.7	60	29
**10d**	0.4	0.2	11	2
**10e**	1.7	0.7	52	6

^1^ Percentage of remaining compound after 60 min of incubation with human and mouse microsomes obtained from Tebu-Xenotech in the presence of NADPH at 37 °C. See Materials and Methods for further details. The ^2^ IC_50_ values are the average of three replicates. The fluorescent assay as performed here has a standard error between 10 and 20% suggesting that differences of two-fold or greater are significant. Because of the limitations of the assay, it is difficult to distinguish among potencies < 0.5 nM [28].

**Table 2:**
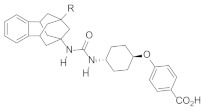
Structures of sEHI **11a–c** and their microsomal stabilities [19].

Cmpd #	R	Microsomal Stability ^1^	
		Human	Mouse
		% Remaining at 1 h	
**11a**	CH_3_	70	10
**11b**	F	77	36
**11c**	Cl	89	60

^1^ Percentage of remaining compound after 60 min of incubation with human and mouse microsomes obtained from Tebu-Xenotech in the presence of NADPH at 37 °C. See Materials and Methods for further details.

## Data Availability

Data is contained within the article and Supplementary Materials.

## Supplementary Materials

The following are available online at https://www.mdpi.com/article/10.3390/ph14121323/s1, ^1^H and ^13^C NMR spectra of the evaluated compounds.
