# Supplementary material for: 2-(Piperidin-4-yl)acetamides as Potent Inhibitors of Soluble Epoxide Hydrolase with Anti-Inflammatory Activity
Source: Pharmaceuticals (Basel). 2021 Dec 17;14(12):1323. doi: 10.3390/ph14121323 (PMC8703317; doi:10.3390/ph14121323)

# SUPPLEMENTARY MATERIAL FOR

## 2-(Piperidin-4-yl)acetamides as potent inhibitors of soluble epoxide hydrolase with anti-inflammatory activity


**Juan Martín-López[1], Sandra Codony[1], Clara Bartra[2], Christophe Morisseau[3], M. Isabel Loza[4], Coral Sanfeliu[2], Bruce D. Hammock[3], José Brea[4,*] and Santiago Vázquez[1,*]**

[1] Laboratori de Química Farmacèutica (Unitat Associada al CSIC), Facultat de Farmàcia i Ciències de l'Alimentació, and Institute of Biomedicine (IBUB), Universitat de Barcelona, Av. Joan XXIII, 27-31, 08028 Barcelona, Spain; juanxi.martin@gmail.com (J.M.-L.); sandra.codony@ub.edu (S.C.)

[2] Department of Entomology and Nematology and Comprehensive Cancer Center, University of California Davis, One Shields Avenue, Davis CA 95616, USA; chmorisseau@ucdavis.edu (C.M.); bdhammock@ucdavis.edu (B.D.H.)

[3] Drug Screening Platform/Biofarma Research Group, CIMUS Research Center. Departamento de Farmacoloxía, Farmacia e Tecnoloxía Farmacéutica. University of Santiago de Compostela (USC), 15782 Santiago de Compostela, Spain; mabel.loza@usc.es (M.I.L.)

* Correspondences: pepo.brea@usc.es (J.B.); svazquez@ub.edu (S.V.); Tel.: +34-881-815-459 (J.B.); +34-934-024-533 (S.V.)

# Index

$^{1}H$ and $^{13}C$ NMR spectra of the synthesized compounds:


*t*-Butyl 4-(2-((9-methyl-5,6,8,9,10,11-hexahydro-7*H*-5,9:7,11-dimethanobenzo[9] annulen-7-yl)amino)-2-oxoethyl)piperidine-1-carboxylate, **4a** Page S1

*N*-(9-methyl-5,6,8,9,10,11-hexahydro-7*H*-5,9:7,11-dimethanobenzo[9]annulen-7-yl)-2-(piperidin-4-yl)acetamide, **5a** Page S2

2-(1-acetylpiperidin-4-yl)-*N*-(9-methyl-5,6,8,9,10,11-hexahydro-7*H*-5,9:7,11-dimethanobenzo[9]annulen-7-yl)acetamide, **6a** Page S3

2-[1-(isopropylsulfonyl)piperidin-4-yl]-*N*-(9-methyl-5,6,8,9,10,11-hexahydro-7*H*-5,9:7,11-dimethanobenzo[9]annulen-7-yl)acetamide, **6b** Page S4

*N*-(5,6,8,9,10,11-hexahydro-7*H*-5,9:7,11-dimethanobenzo[9]annulen-7-yl)-2-(piperidin-4-yl)acetamide, **5b** Page S5

*N*-(5,6,8,9,10,11-hexahydro-7*H*-5,9:7,11-dimethanobenzo[9]annulen-7-yl)-2-(1-(isopropylsulfonyl)piperidin-4-yl)acetamide, **6e** Page S6

*N*-(5,6,8,9,10,11-hexahydro-7*H*-5,9:7,11-dimethanobenzo[9]annulen-7-yl-9-d)-2-(piperidin-4-yl)acetamide, **5c** Page S7

*N*-(5,6,8,9,10,11-hexahydro-7*H*-5,9:7,11-dimethanobenzo[9]annulen-7-yl-9-*d*)-2-(1-(isopropylsulfonyl)piperidin-4-yl)acetamide, **6f** Page S8

*t*-butyl 4-(2-((9-fluoro-5,6,8,9,10,11-hexahydro-7*H*-5,9:7,11-dimethanobenzo[9] annulen-7-yl)amino)-2-oxoethyl)piperidine-1-carboxylate, **4d** Page S9

*N*-(9-fluoro-5,6,8,9,10,11-hexahydro-7*H*-5,9:7,11-dimethanobenzo[9]annulen-7-

yl)-2-(piperidin-4-yl)acetamide, **5d** Page S10

*N*-(9-fluoro-5,6,8,9,10,11-hexahydro-7*H*-5,9:7,11-dimethanobenzo[9]annulen-7-yl)-2-(1-(isopropylsulfonyl)piperidin-4-yl)acetamide, **6c** Page S11

*t*-butyl 4-(2-((9-chloro-5,6,8,9,10,11-hexahydro-7*H*-5,9:7,11-dimethanobenzo[9]annulen-7-yl)amino)-2-oxoethyl)piperidine-1-carboxylate, **4e** Page S12

*N*-(9-chloro-5,6,8,9,10,11-hexahydro-7*H*-5,9:7,11-dimethanobenzo[9]annulen-7-yl)-2-(piperidin-4-yl)acetamide hydrochloride, **5e** Page S13

*N*-(9-chloro-5,6,8,9,10,11-hexahydro-7*H*-5,9:7,11-dimethanobenzo[9]annulen-7-yl)-2-(1-(isopropylsulfonyl)piperidin-4-yl)acetamide, **6d** Page S14

2-(1-benzylpiperidin-4-yl)-*N*-(9-methyl-5,6,8,9,10,11-hexahydro-7*H*-5,9:7,11-dimethanobenzo[9]annulen-7-yl)acetamide, **6g** Page S15

2-(1-benzylpiperidin-4-yl)-N-(9-fluoro-5,6,8,9,10,11-hexahydro-7*H*-5,9:7,11-dimethanobenzo[9]annulen-7-yl)acetamide, **6h** Page S16

2-(1-(4-(*t*-butoxycarbonyl)phenyl)piperidin-4-yl)acetic acid, **9c** Page S17

2-(1-(2-bromo-4-(methoxycarbonyl)phenyl)piperidin-4-yl)acetic acid, **9d** Page S18

2-(1-(2-cyclopropyl-4-(methoxycarbonyl)phenyl)piperidin-4-yl)acetic acid, **9e** Page S19

2-(1-(4-acetylphenyl)piperidin-4-yl)-N-(9-fluoro-5,6,8,9,10,11-hexahydro-7*H*-5,9:7,11-dimethanobenzo[9]annulen-7-yl)acetamide, **10a** Page S20

2-(1-(4-cyanophenyl)piperidin-4-yl)-N-(9-fluoro-5,6,8,9,10,11-hexahydro-7*H*-5,9:7,11-dimethanobenzo[9]annulen-7-yl)acetamide, **10b** Page S21

4-(4-(2-((9-fluoro-5,6,8,9,10,11-hexahydro-7*H*-5,9:7,11-dimethanobenzo[9]annulen-

7-yl)amino)-2-oxoethyl)piperidin-1-yl)benzoic acid, **10c** Page S22

3-cyclopropyl-4-(4-(2-((9-fluoro-5,6,8,9,10,11-hexahydro-7*H*-5,9:7,11-dimethanobenzo
[9]annulen-7-yl)amino)-2-oxoethyl)piperidin-1-yl)benzoic acid, **10d** Page S23

4-(4-(2-((9-chloro-5,6,8,9,10,11-hexahydro-7*H*-5,9:7,11-dimethanobenzo[9]annulen-
7-yl)amino)-2-oxoethyl)piperidin-1-yl)benzoic acid, **10e** Page S24

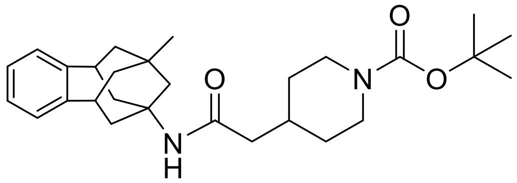


**4a**

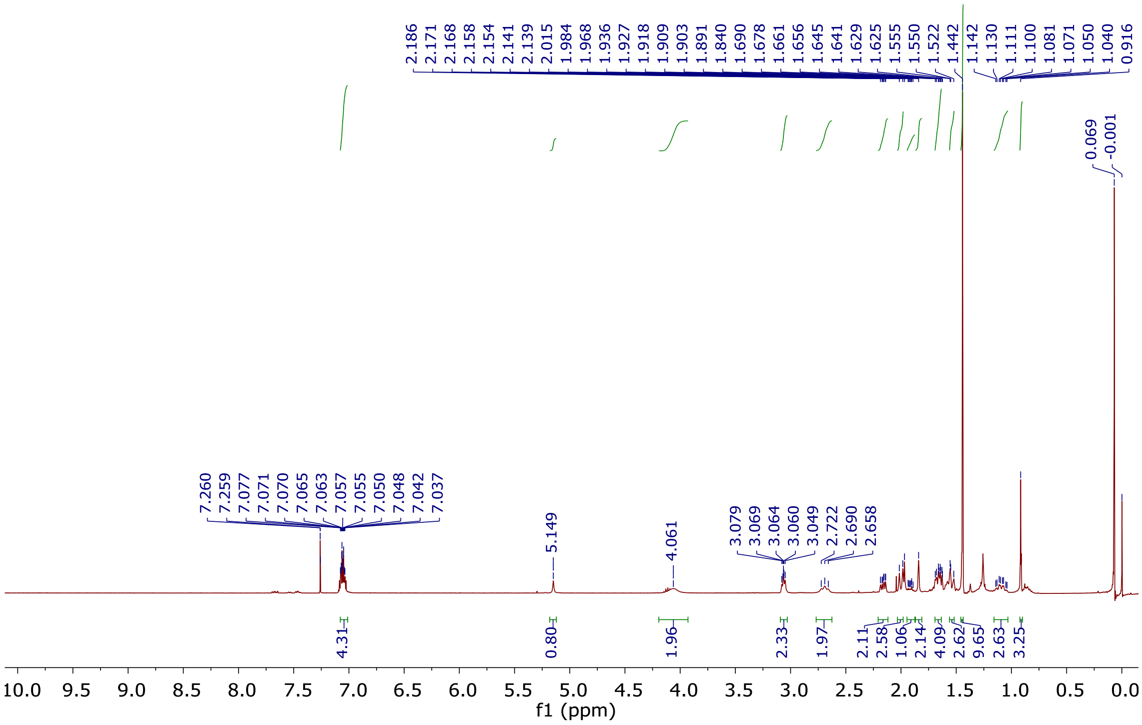

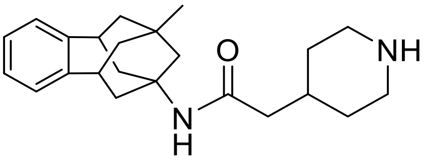
O
NH
N
H

**5a**

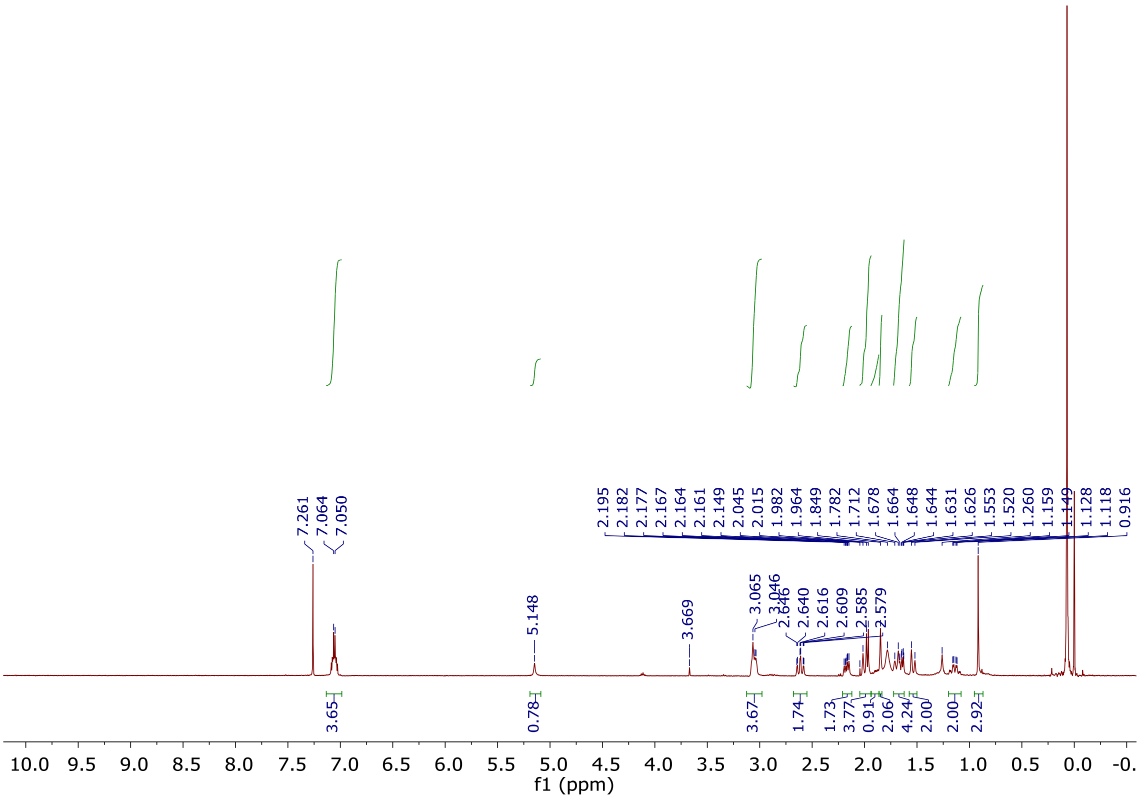
7.261
7.064
7.050
5.148
3.669
3.065
3.046
2.640
2.616
2.609
2.585
2.579
2.195
2.182
2.177
2.167
2.164
2.161
2.149
2.045
2.015
1.982
1.964
1.849
1.782
1.712
1.678
1.664
1.648
1.644
1.631
1.626
1.553
1.520
1.260
1.159
1.149
1.128
1.118
0.916
3.65
0.78
3.67
1.74
1.73
3.77
0.91
2.06
4.24
2.00
2.00
2.92
10.0 9.5 9.0 8.5 8.0 7.5 7.0 6.5 6.0 5.5 5.0 4.5 4.0 3.5 3.0 2.5 2.0 1.5 1.0 0.5 0.0 -0.
f1 (ppm)

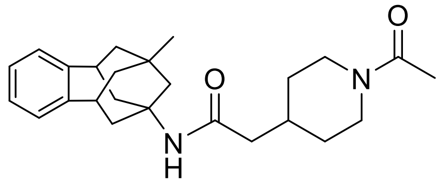
O
N
O
N
H

**6a**

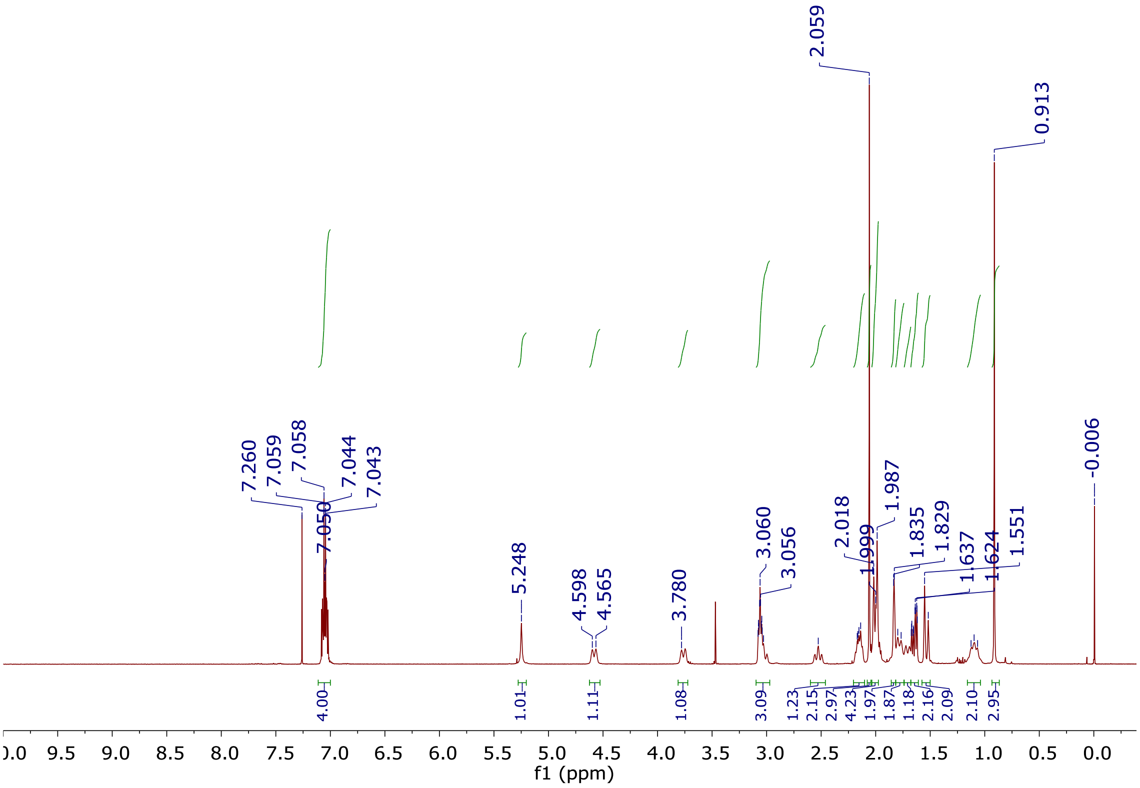
2.059
0.913
7.260
7.059
7.058
7.050
7.044
7.043
5.248
4.598
4.565
3.780
3.060
3.056
2.018
1.999
1.987
1.835
1.829
1.637
1.624
1.551
-0.006
4.00
1.01
1.11
1.08
3.09
1.23
2.15
2.97
4.23
1.97
1.87
1.18
2.16
2.09
2.10
2.95
f1 (ppm)

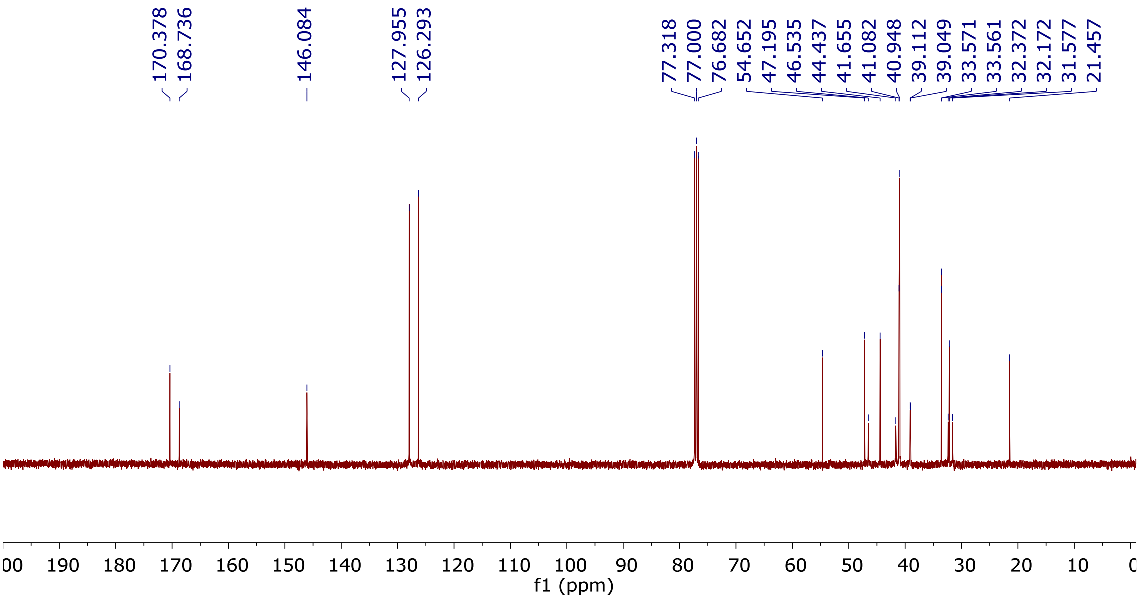
170.378
168.736
146.084
127.955
126.293
77.318
77.000
76.682
54.652
47.195
46.535
44.437
41.655
41.082
40.948
39.112
39.049
33.571
33.561
32.372
32.172
31.577
21.457
f1 (ppm)

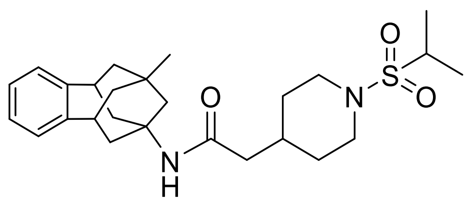

**6b**

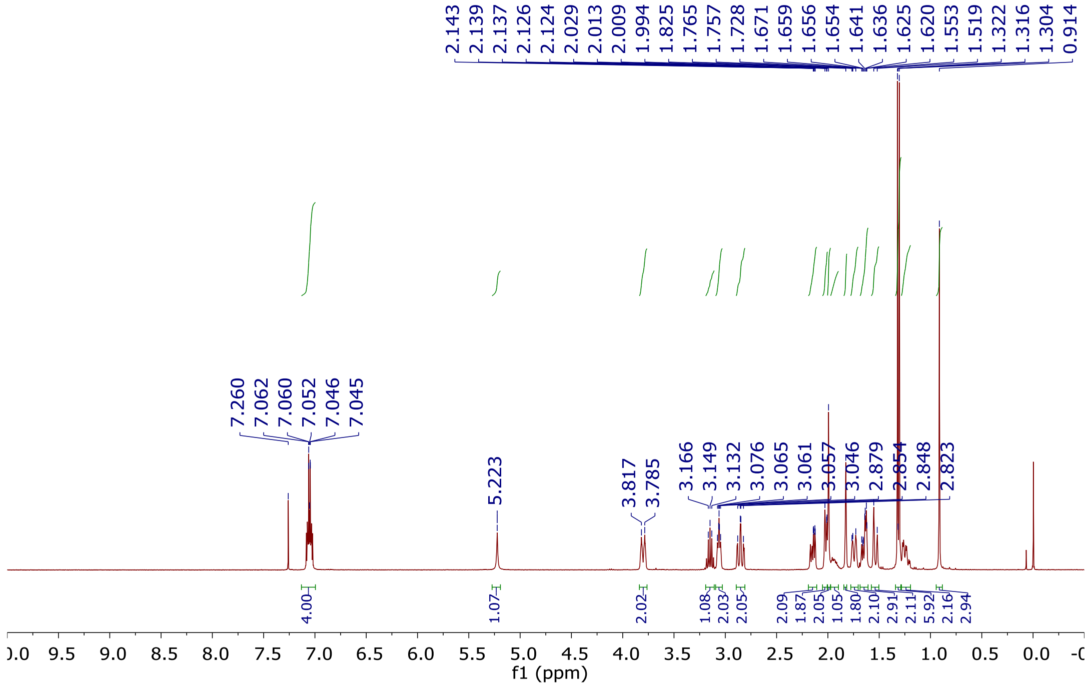

2.143
2.139
2.137
2.126
2.124
2.029
2.013
2.009
1.994
1.825
1.765
1.757
1.728
1.671
1.659
1.656
1.654
1.641
1.636
1.625
1.620
1.553
1.519
1.322
1.316
1.304
0.914
7.260
7.062
7.060
7.052
7.046
7.045
5.223
3.817
3.785
3.166
3.149
3.132
3.076
3.065
3.061
3.057
3.046
2.879
2.854
2.848
2.823
4.00
1.07
2.02
1.08
2.03
2.05
2.09
1.87
2.05
1.05
1.80
2.10
2.91
2.11
5.92
2.16
2.94
f1 (ppm)


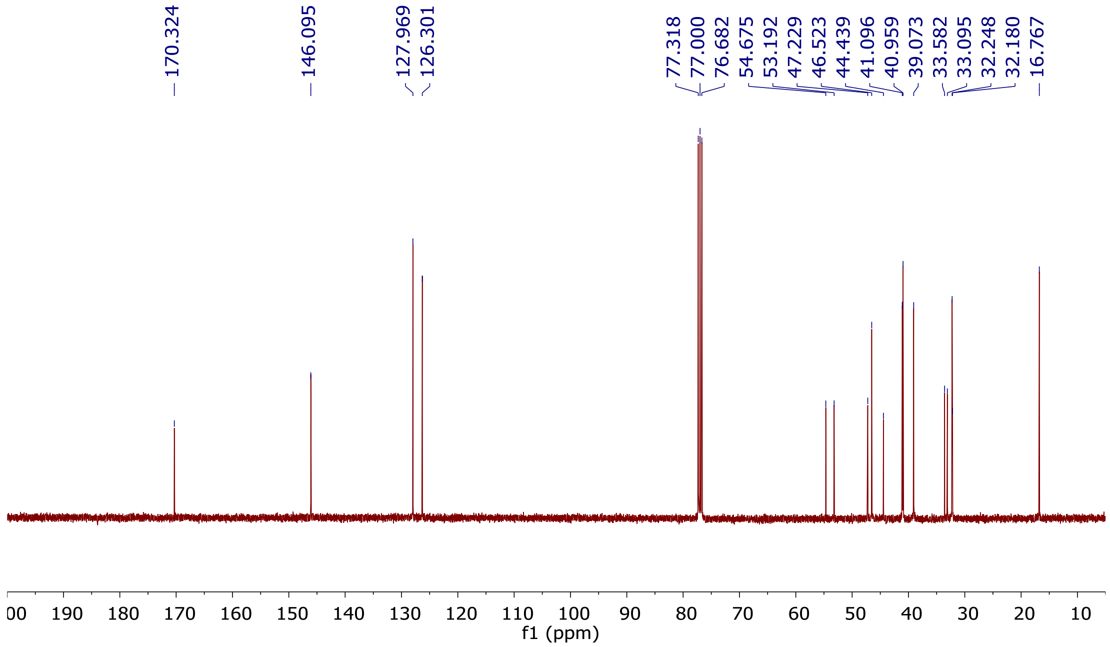

170.324
146.095
127.969
126.301
77.318
77.000
76.682
54.675
53.192
47.229
46.523
44.439
41.096
40.959
39.073
33.582
33.095
32.248
32.180
16.767
f1 (ppm)

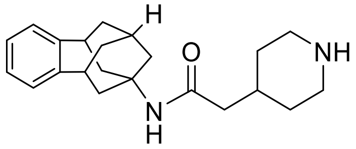
H
O
NH
N
H

**5b**

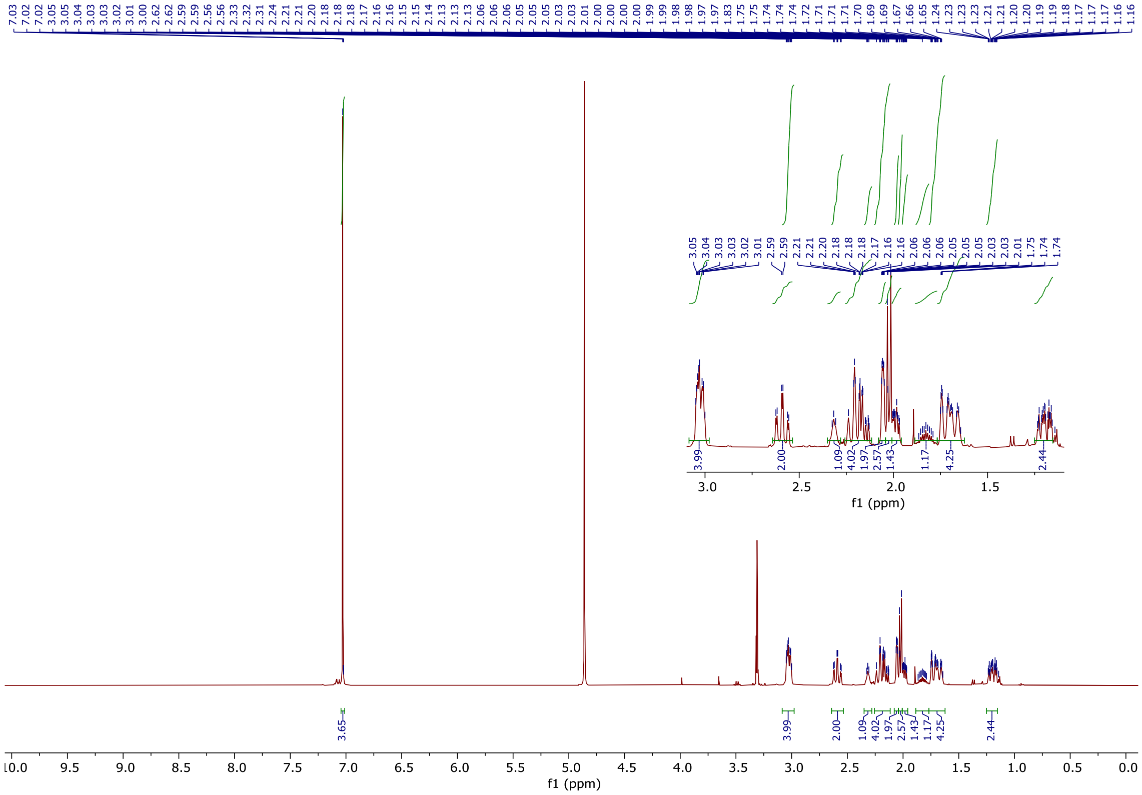
7.03 7.02 7.02 3.05 3.05 3.04 3.03 3.03 3.02 3.01 3.00 2.62 2.59 2.59 2.56 2.56 2.33 2.32 2.31 2.24 2.21 2.20 2.18 2.18 2.17 2.16 2.16 2.15 2.15 2.14 2.13 2.13 2.13 2.06 2.06 2.06 2.05 2.05 2.05 2.03 2.03 2.01 2.00 2.00 2.00 1.99 1.99 1.98 1.98 1.97 1.97 1.83 1.75 1.75 1.74 1.74 1.74 1.72 1.71 1.71 1.71 1.70 1.69 1.69 1.67 1.66 1.65 1.24 1.23 1.23 1.23 1.21 1.21 1.20 1.20 1.19 1.19 1.18 1.17 1.17 1.17 1.16 1.16
3.05 3.04 3.03 3.03 3.02 3.01 2.59 2.59 2.21 2.21 2.20 2.18 2.18 2.18 2.17 2.16 2.16 2.06 2.06 2.06 2.05 2.05 2.05 2.03 2.03 2.01 1.75 1.74 1.74
3.99 2.00 1.09 4.02 1.97 2.57 1.43 1.17 4.25 2.44
3.0 2.5 2.0 1.5
f1 (ppm)
3.65 3.99 2.00 1.09 4.02 1.97 2.57 1.43 1.17 4.25 2.44
10.0 9.5 9.0 8.5 8.0 7.5 7.0 6.5 6.0 5.5 5.0 4.5 4.0 3.5 3.0 2.5 2.0 1.5 1.0 0.5 0.0
f1 (ppm)

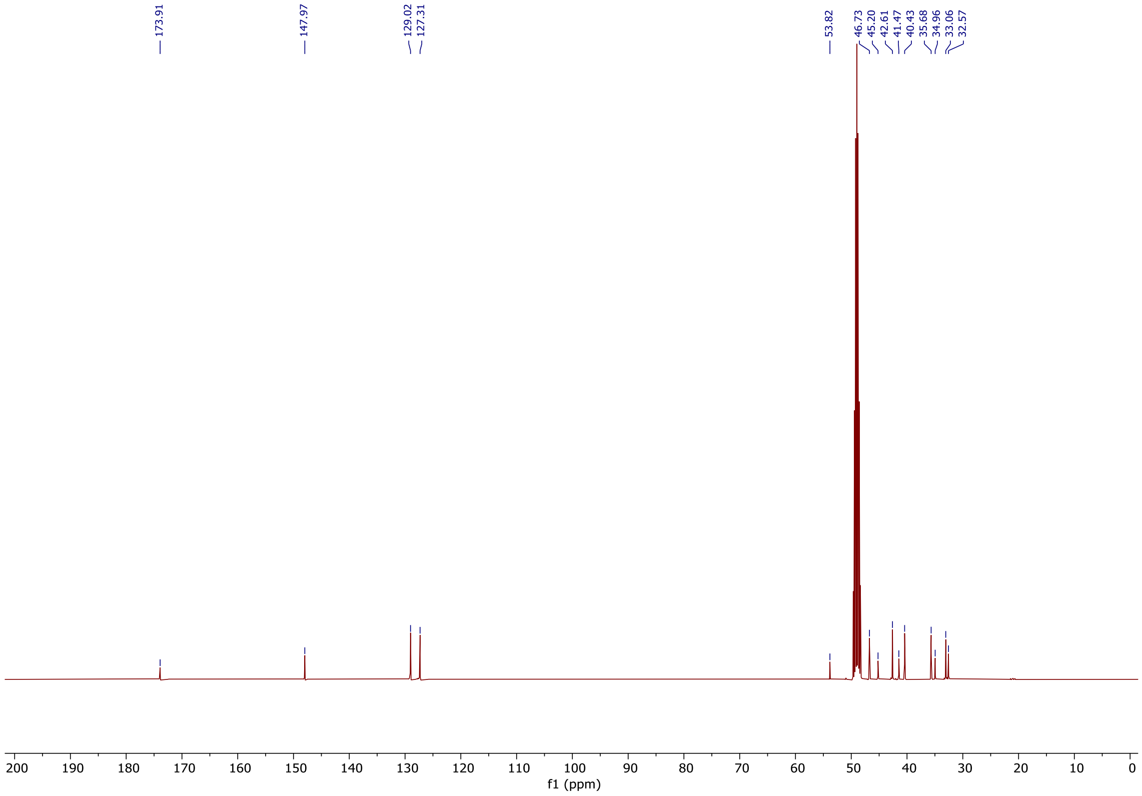
173.91
147.97
129.02 127.31
53.82
46.73 45.20 42.61 41.47 40.43 35.68 34.96 33.06 32.57
200 190 180 170 160 150 140 130 120 110 100 90 80 70 60 50 40 30 20 10 0
f1 (ppm)

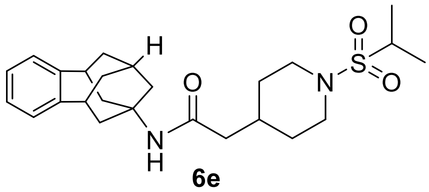

**6e**

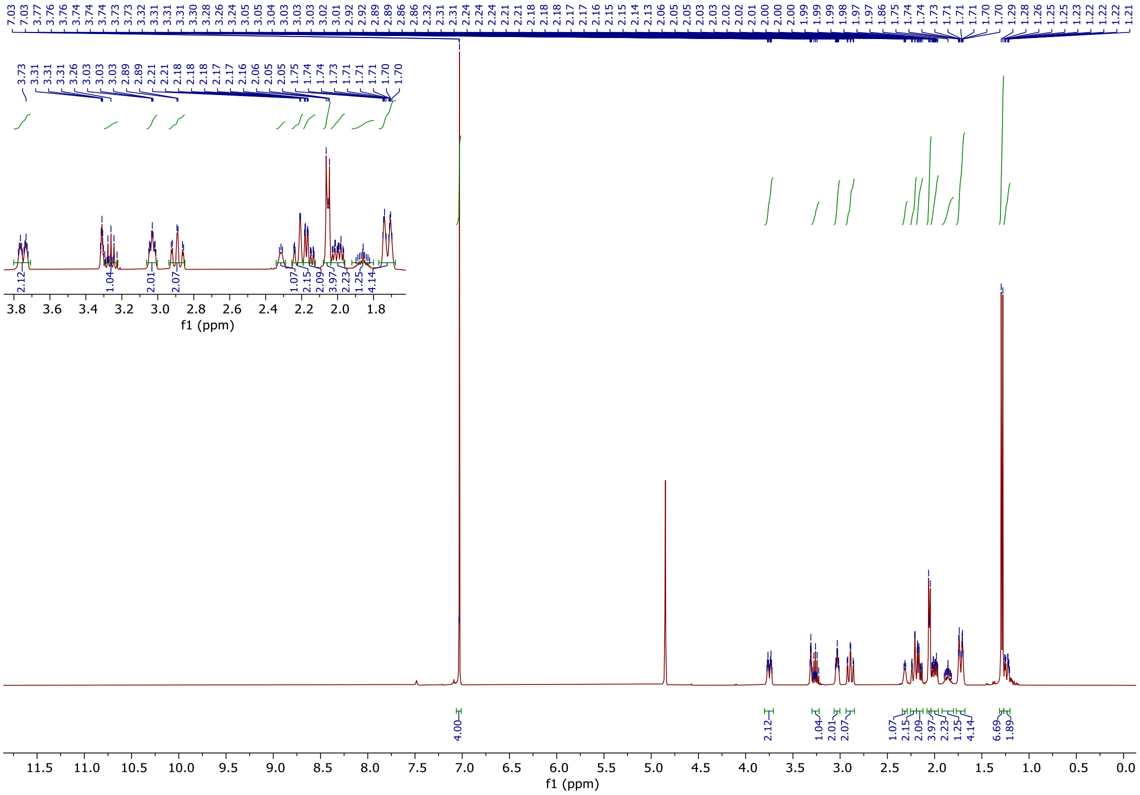

7.03 7.03 3.77 3.76 3.76 3.74 3.74 3.73 3.73 3.32 3.31 3.31 3.31 3.30 3.28 3.26 3.24 3.05 3.05 3.04 3.03 3.03 3.03 3.02 3.01 2.92 2.92 2.89 2.89 2.86 2.86 2.32 2.31 2.31 2.24 2.24 2.24 2.21 2.21 2.18 2.18 2.18 2.17 2.16 2.15 2.15 2.14 2.13 2.06 2.05 2.05 2.03 2.03 2.02 2.02 2.01 2.00 2.00 2.00 1.99 1.99 1.99 1.98 1.97 1.97 1.86 1.75 1.74 1.74 1.73 1.71 1.71 1.71 1.70 1.70 1.29 1.28 1.26 1.25 1.25 1.23 1.23 1.22 1.22 1.21
3.73 3.31 3.31 3.31 3.03 3.03 3.03 2.89 2.89 2.21 2.21 2.18 2.18 2.17 2.16 2.06 2.05 2.05 1.75 1.74 1.74 1.73 1.71 1.71 1.71 1.70 1.70
2.12 1.04 2.01 2.07 1.07 2.15 2.09 3.97 2.23 1.25 4.14
3.8 3.6 3.4 3.2 3.0 2.8 2.6 2.4 2.2 2.0 1.8
f1 (ppm)
4.00 2.12 1.04 2.01 2.07 1.07 2.15 2.09 3.97 2.23 1.25 4.14 6.69 1.89
11.5 11.0 10.5 10.0 9.5 9.0 8.5 8.0 7.5 7.0 6.5 6.0 5.5 5.0 4.5 4.0 3.5 3.0 2.5 2.0 1.5 1.0 0.5 0.0
f1 (ppm)


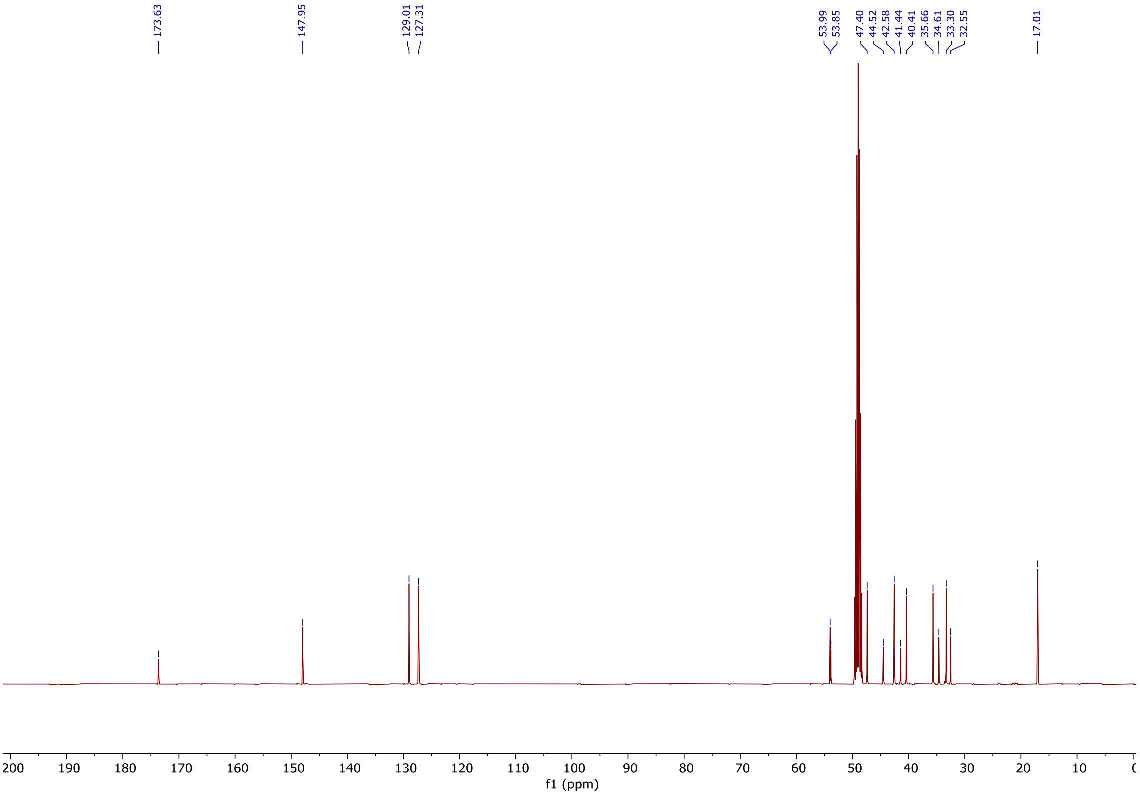

173.63
147.95
129.01 127.31
53.99 53.85
47.40 44.52 42.58 41.44 40.41 35.66 34.61 33.30 32.55
17.01
200 190 180 170 160 150 140 130 120 110 100 90 80 70 60 50 40 30 20 10 0
f1 (ppm)

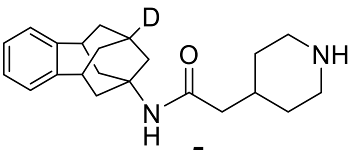
D
O
NH
N
H

**5c**

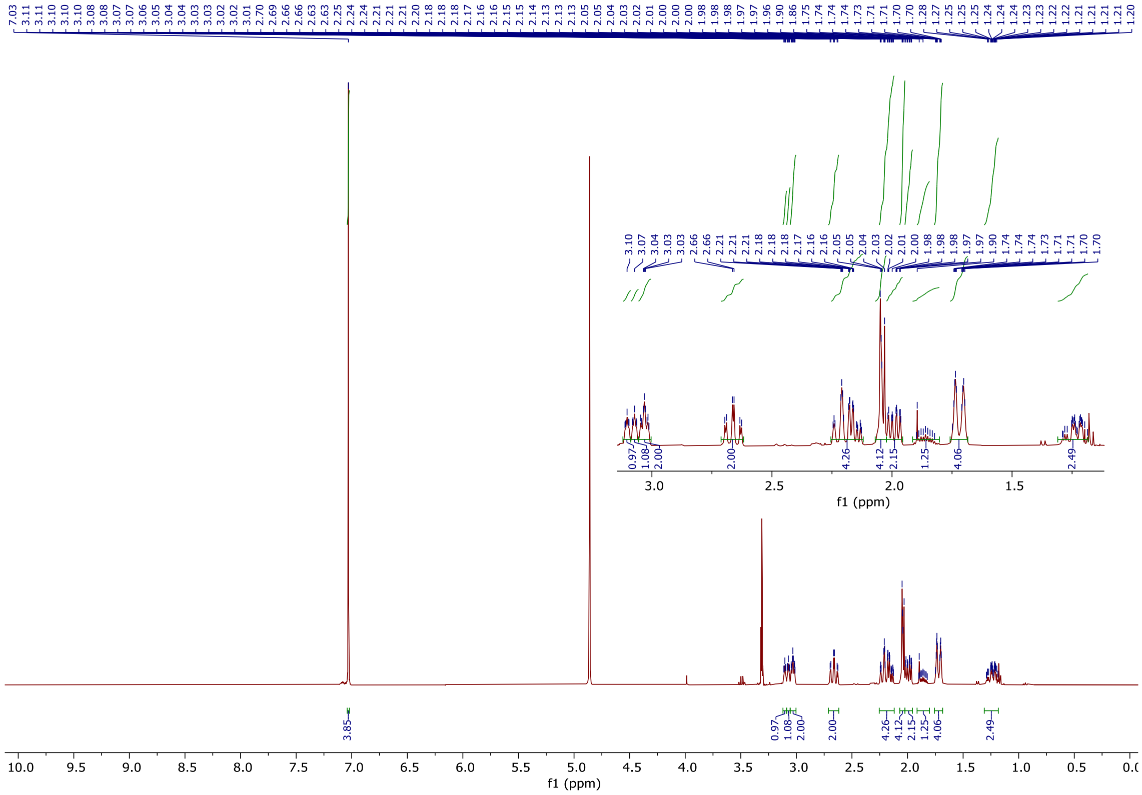
7.03
3.11
3.11
3.10
3.10
3.08
3.08
3.07
3.06
3.05
3.04
3.03
3.03
3.02
3.02
3.01
2.70
2.69
2.66
2.66
2.63
2.63
2.25
2.24
2.24
2.21
2.21
2.20
2.18
2.18
2.18
2.17
2.16
2.16
2.15
2.15
2.14
2.13
2.13
2.13
2.05
2.05
2.04
2.03
2.02
2.01
2.00
2.00
1.98
1.98
1.98
1.97
1.97
1.96
1.90
1.86
1.75
1.74
1.74
1.73
1.71
1.71
1.70
1.70
1.28
1.27
1.25
1.25
1.24
1.24
1.24
1.23
1.23
1.22
1.22
1.21
1.21
1.21
1.20
3.85
0.97
1.08
2.00
2.00
4.26
4.12
2.15
1.25
4.06
2.49
f1 (ppm)

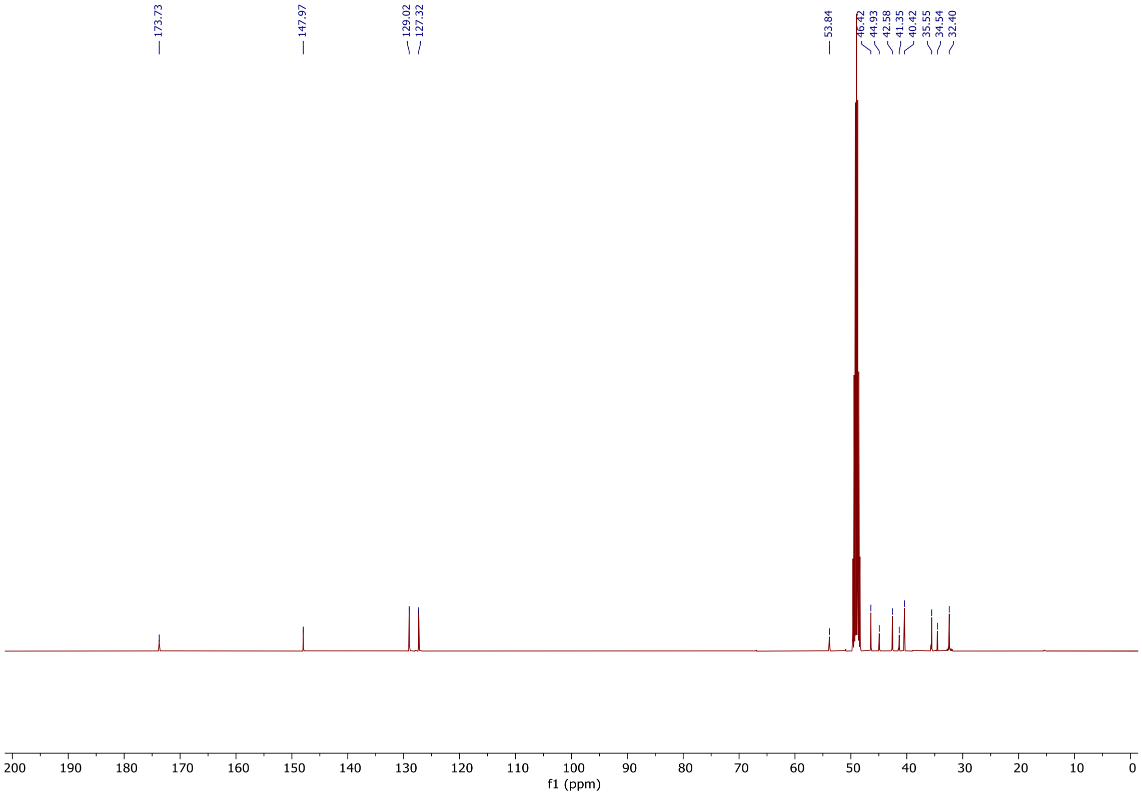
173.73
147.97
129.02
127.32
53.84
46.42
44.93
42.58
41.35
40.42
35.55
34.54
32.40
f1 (ppm)

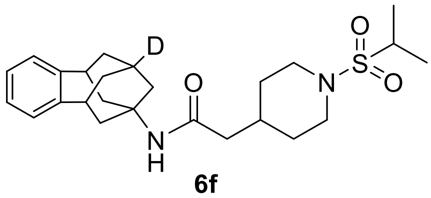
D
O
N
H
S
O
O
N

**6f**

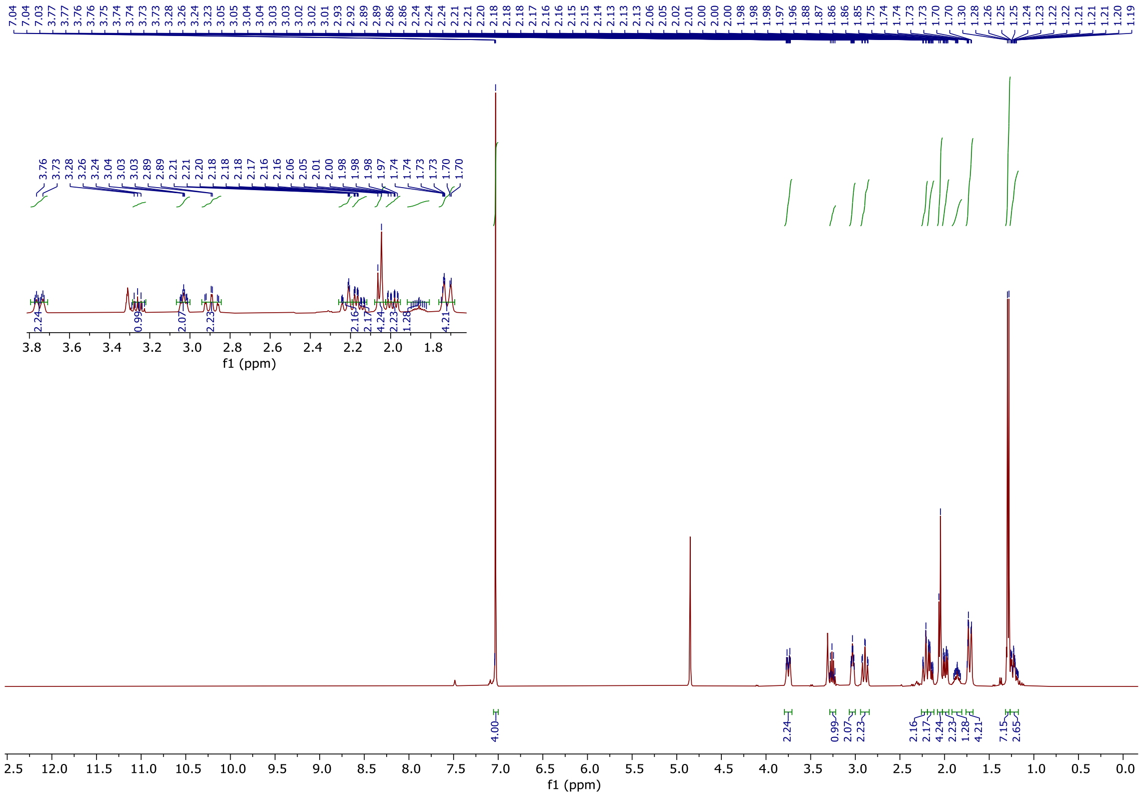
f1 (ppm)
f1 (ppm)

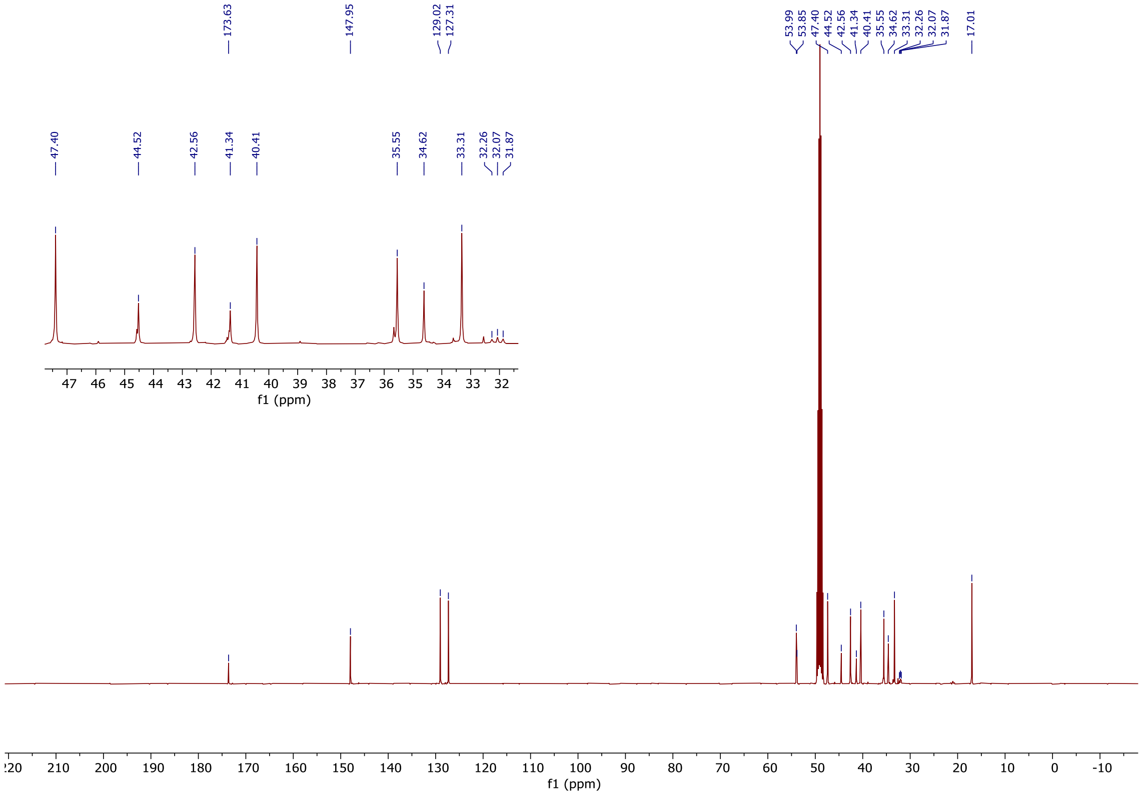
173.63
147.95
129.02
127.31
53.99
53.85
47.40
44.52
42.56
41.34
40.41
35.55
34.62
33.31
32.26
32.07
31.87
17.01
f1 (ppm)
f1 (ppm)

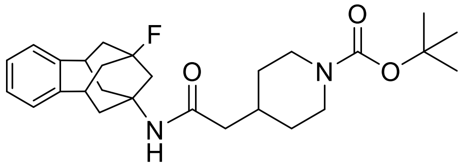

**4d**

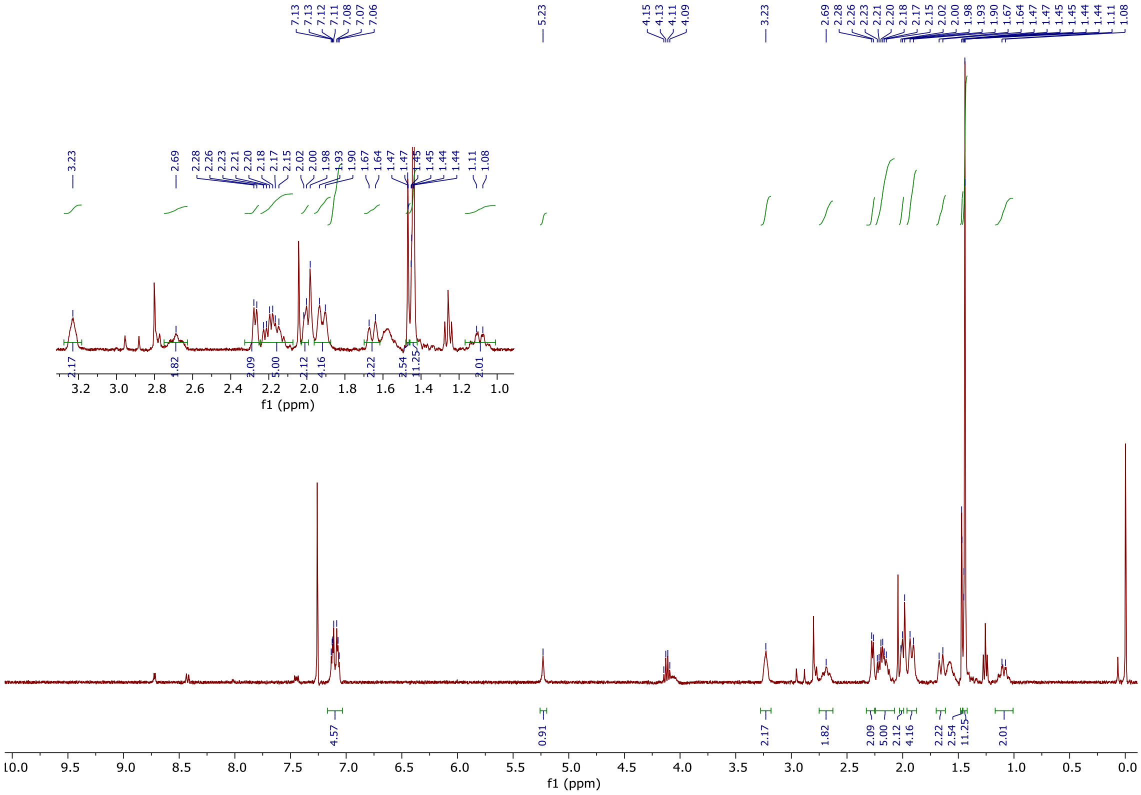

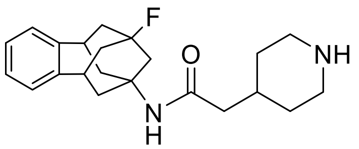
F
O
NH
N
H

**5d**

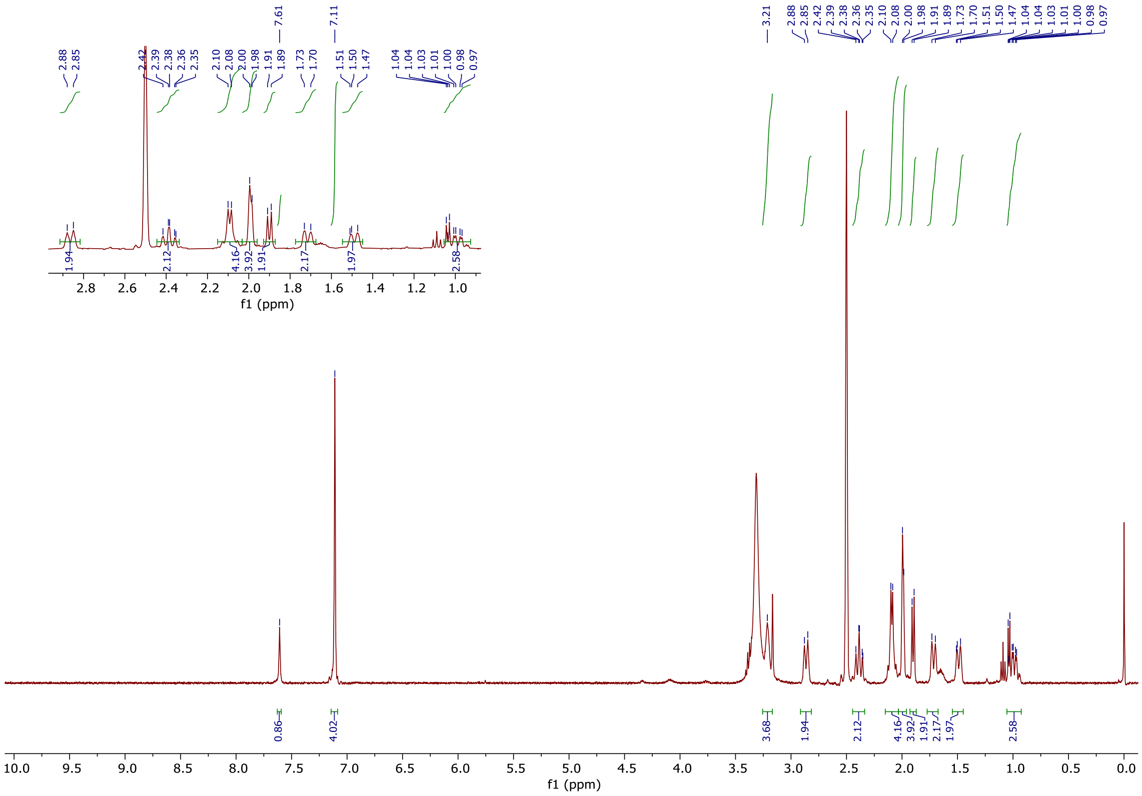
3.21
2.88
2.85
2.42
2.39
2.38
2.36
2.35
2.10
2.08
2.00
1.98
1.91
1.89
1.73
1.70
1.51
1.50
1.47
1.04
1.04
1.03
1.01
1.00
0.98
0.97
7.61
7.11
1.94
2.12
4.16
3.92
1.91
2.17
1.97
2.58
0.86
4.02
3.68
f1 (ppm)

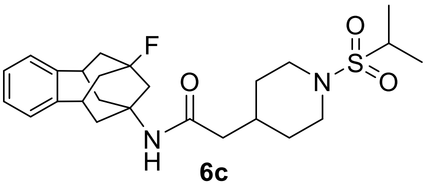
F
O
N
H
S
O
O

**6c**

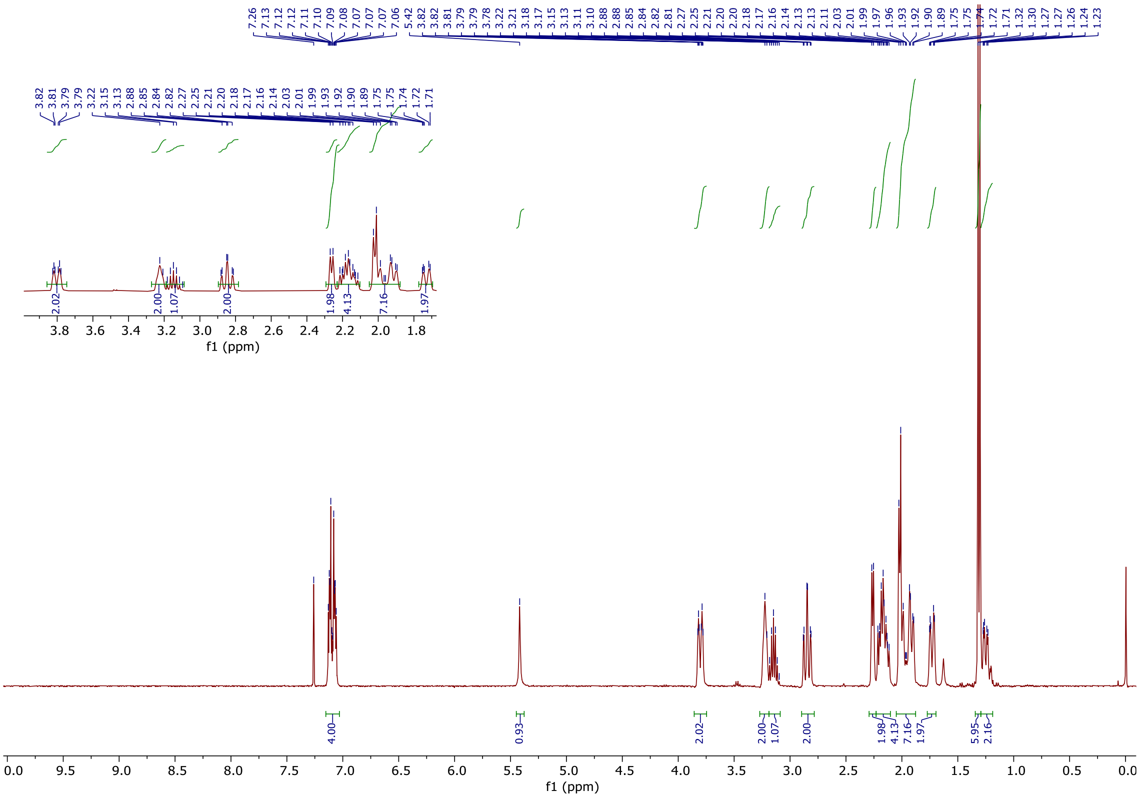
7.26 7.13 7.12 7.12 7.11 7.10 7.09 7.08 7.07 7.07 7.07 7.06 5.42 3.82 3.82 3.81 3.79 3.79 3.78 3.22 3.21 3.18 3.17 3.15 3.13 3.11 3.10 2.88 2.88 2.85 2.84 2.82 2.81 2.27 2.25 2.21 2.20 2.20 2.18 2.17 2.16 2.14 2.13 2.13 2.11 2.03 2.01 1.99 1.97 1.96 1.93 1.92 1.90 1.89 1.75 1.75 1.74 1.72 1.71 1.32 1.30 1.27 1.26 1.24 1.23
3.82 3.81 3.79 3.79 3.22 3.15 3.13 2.88 2.85 2.84 2.82 2.27 2.25 2.21 2.20 2.18 2.17 2.16 2.14 2.03 2.01 1.99 1.93 1.92 1.90 1.89 1.75 1.75 1.74 1.72 1.71
2.02 2.00 1.07 2.00 1.98 4.13 7.16 1.97
3.8 3.6 3.4 3.2 3.0 2.8 2.6 2.4 2.2 2.0 1.8
f1 (ppm)
4.00 0.93 2.02 2.00 1.07 2.00 1.98 4.13 7.16 1.97 5.95 2.16
10.0 9.5 9.0 8.5 8.0 7.5 7.0 6.5 6.0 5.5 5.0 4.5 4.0 3.5 3.0 2.5 2.0 1.5 1.0 0.5 0.0
f1 (ppm)

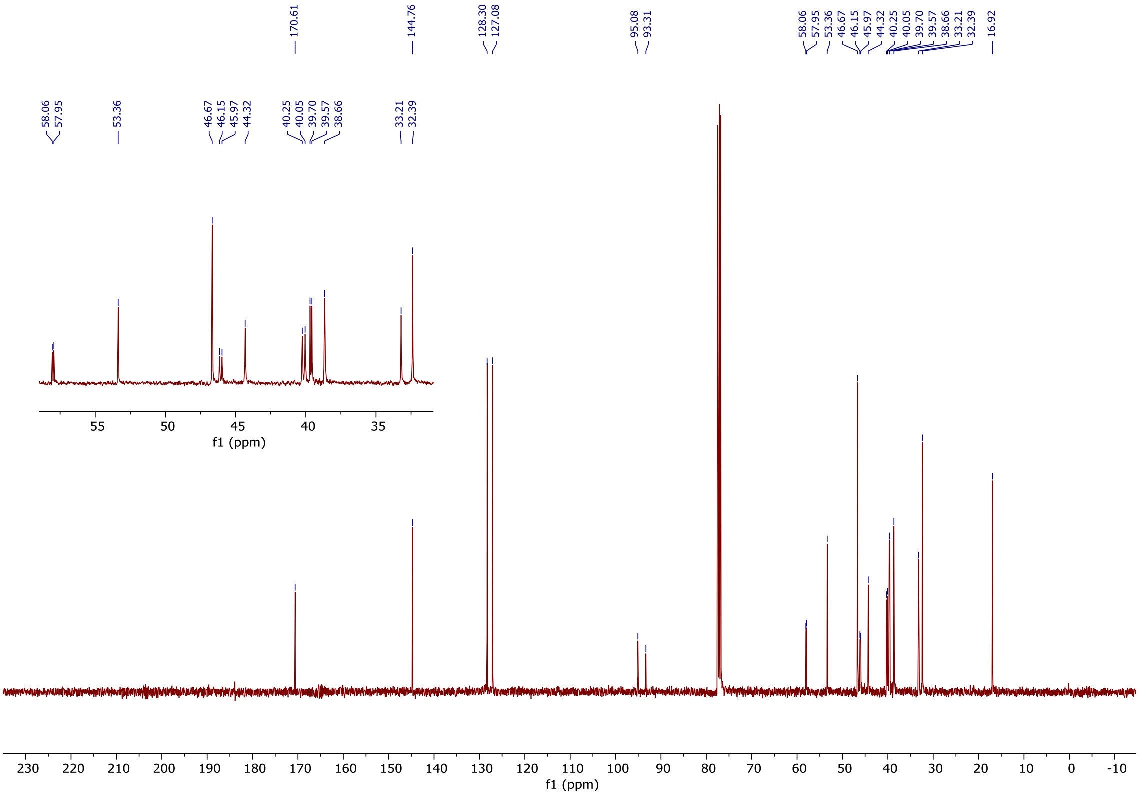
170.61 144.76 128.30 127.08 95.08 93.31 58.06 57.95 53.36 46.67 46.15 45.97 44.32 40.25 40.05 39.70 39.57 38.66 33.21 32.39 16.92
58.06 57.95 53.36 46.67 46.15 45.97 44.32 40.25 40.05 39.70 39.57 38.66 33.21 32.39
55 50 45 40 35
f1 (ppm)
230 220 210 200 190 180 170 160 150 140 130 120 110 100 90 80 70 60 50 40 30 20 10 0 -10
f1 (ppm)

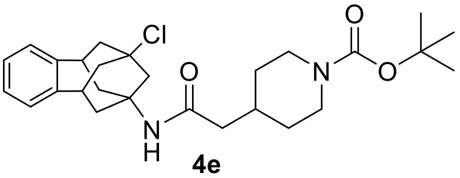

**4e**

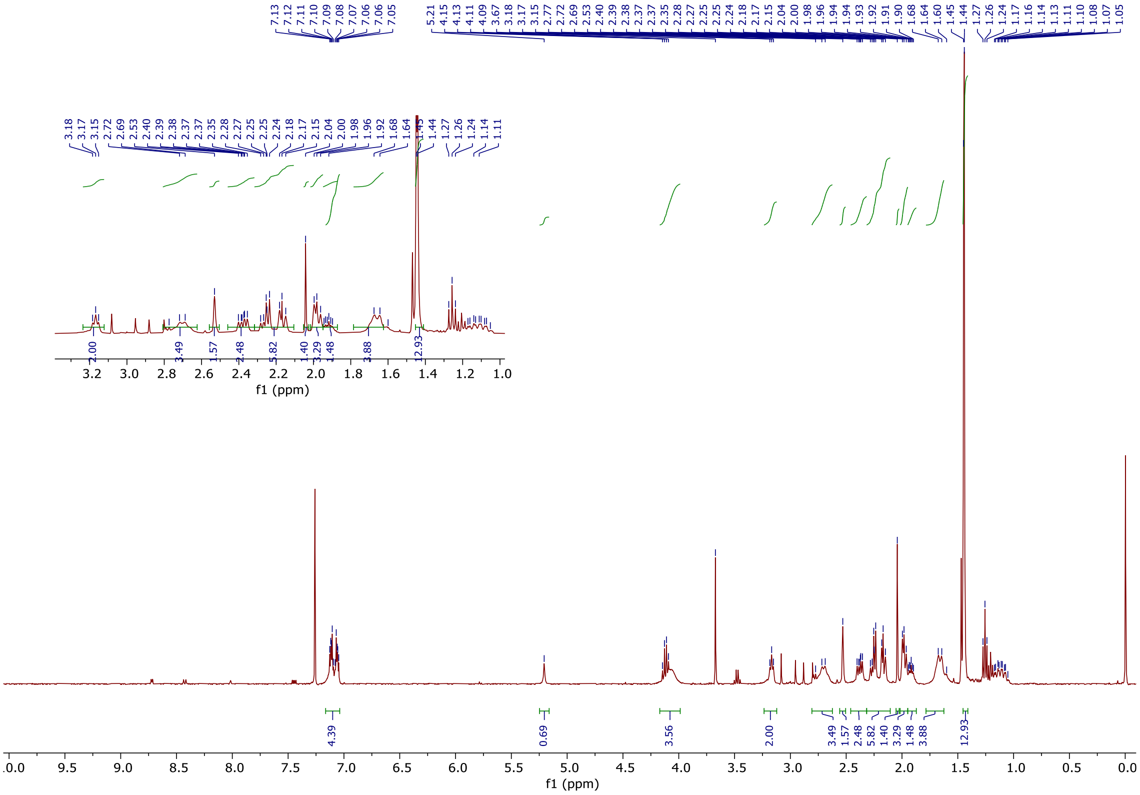

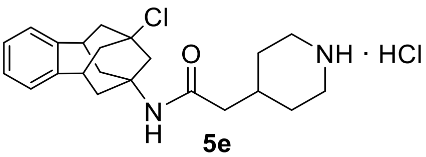
Cl
O
NH · HCl
N
H

**5e**

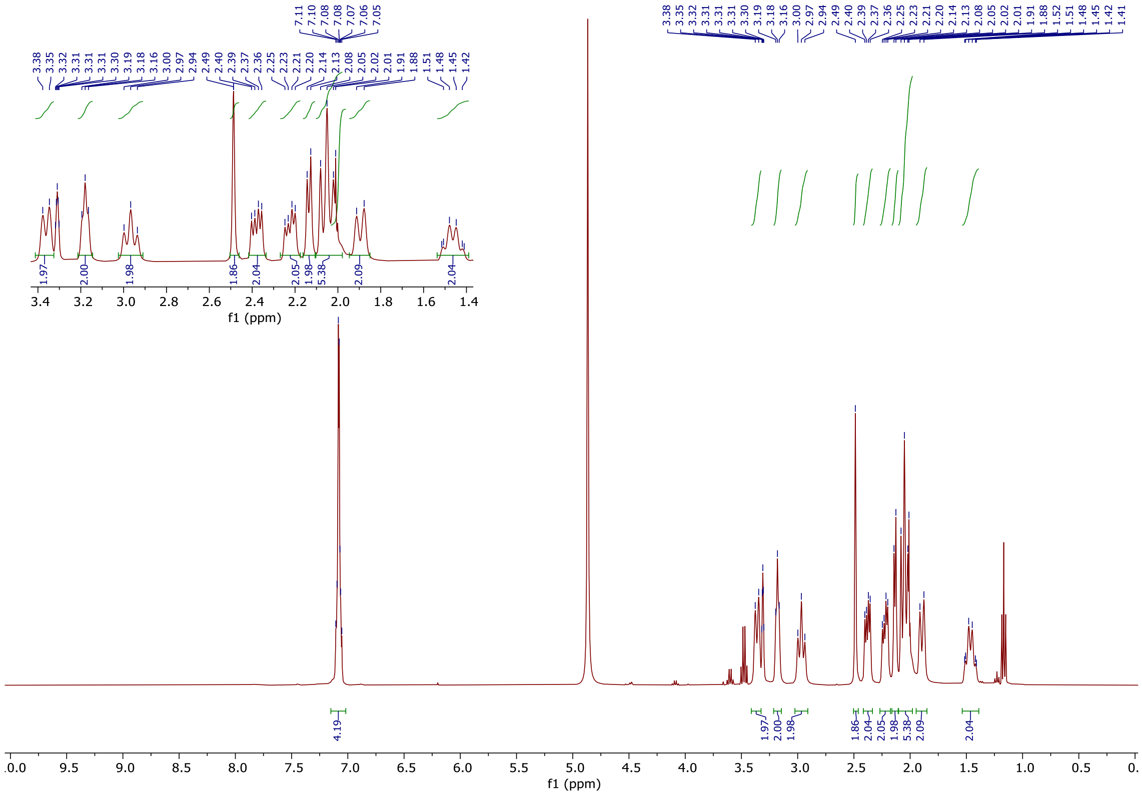
7.11 7.10 7.08 7.08 7.07 7.06 7.05
3.38 3.35 3.32 3.31 3.31 3.31 3.30 3.19 3.18 3.16 3.00 2.97 2.94 2.49 2.40 2.39 2.37 2.36 2.25 2.23 2.21 2.20 2.14 2.13 2.08 2.05 2.02 2.01 1.91 1.88 1.52 1.51 1.48 1.45 1.42 1.41
1.97 2.00 1.98 1.86 2.04 2.05 1.98 5.38 2.09 2.04
4.19
f1 (ppm)

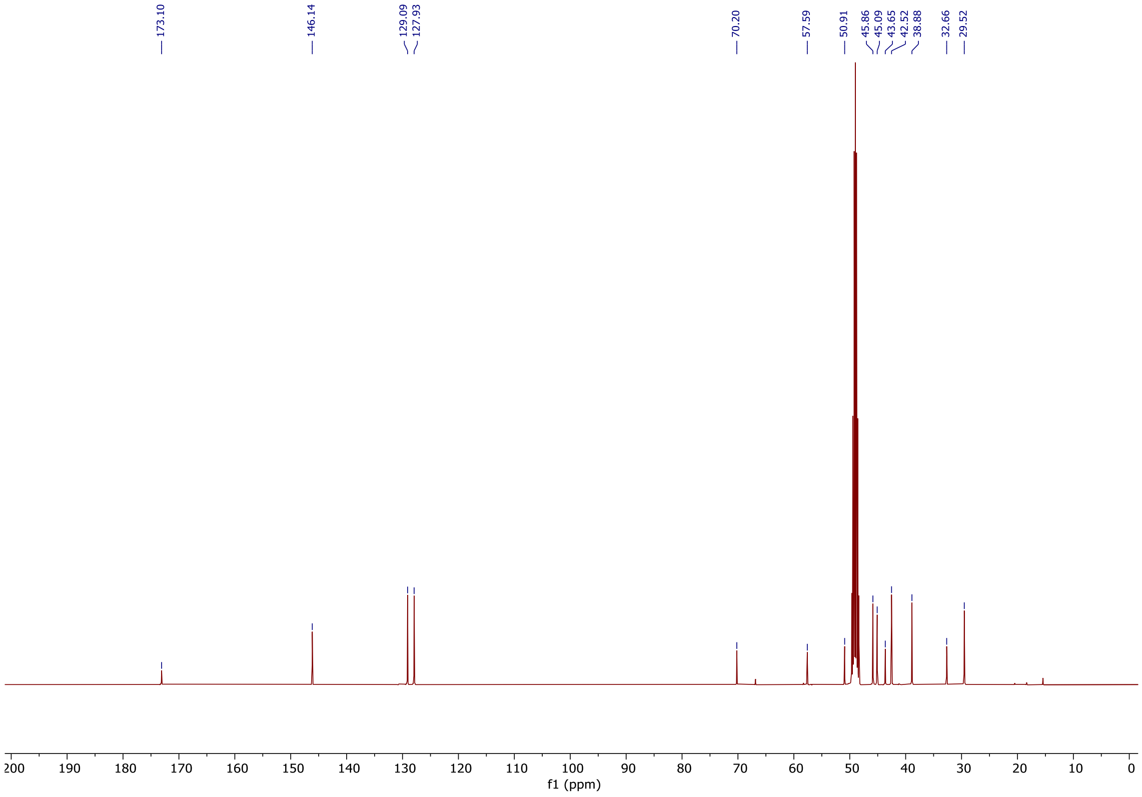
173.10
146.14
129.09 127.93
70.20
57.59
50.91 45.86 45.09 43.65 42.52 38.88
32.66 29.52
f1 (ppm)

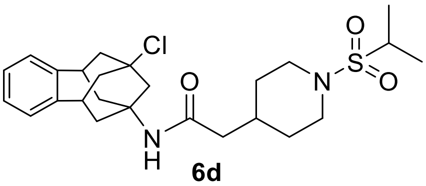


**6d**

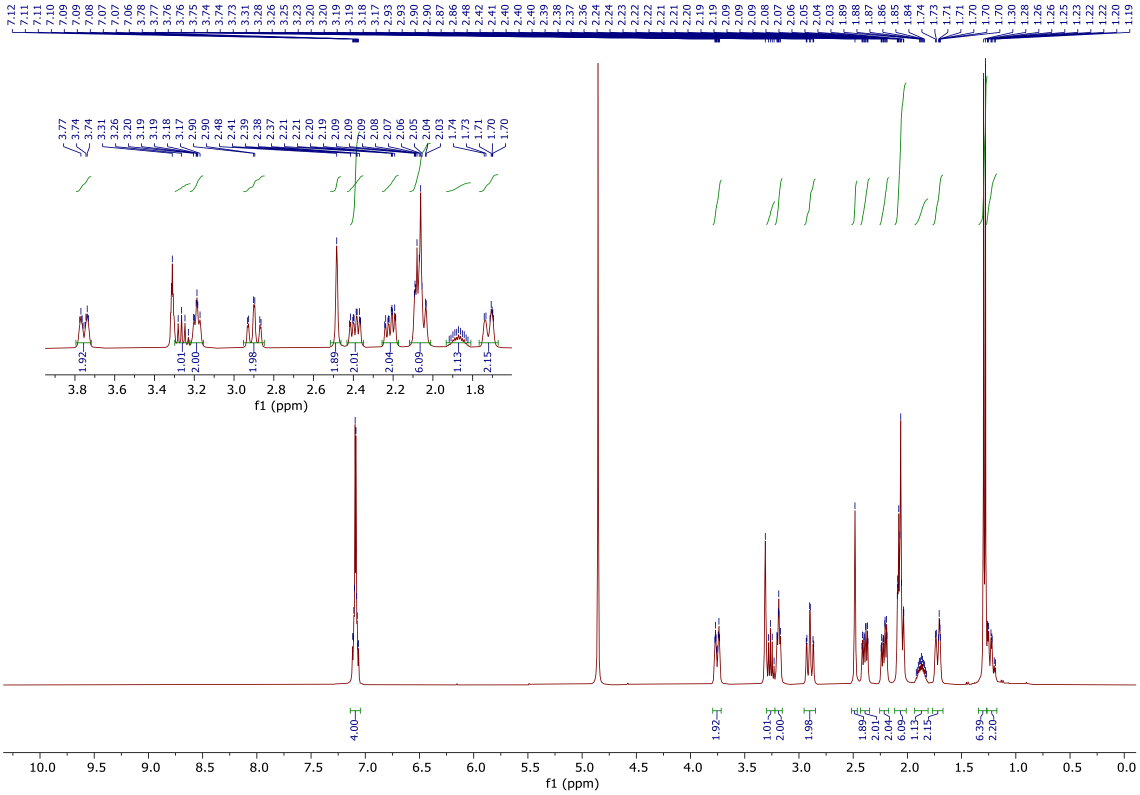


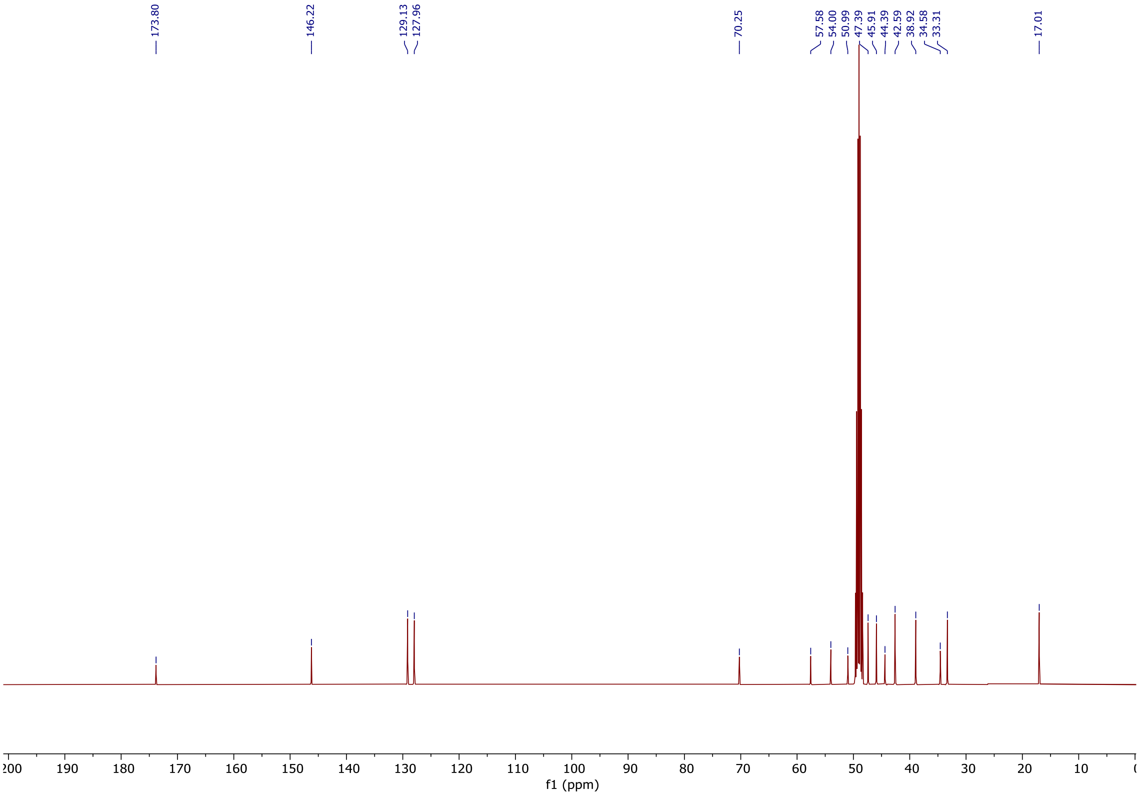

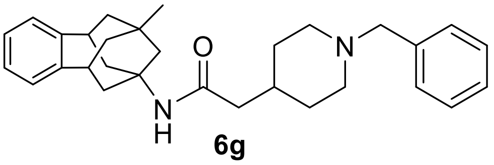

**6g**

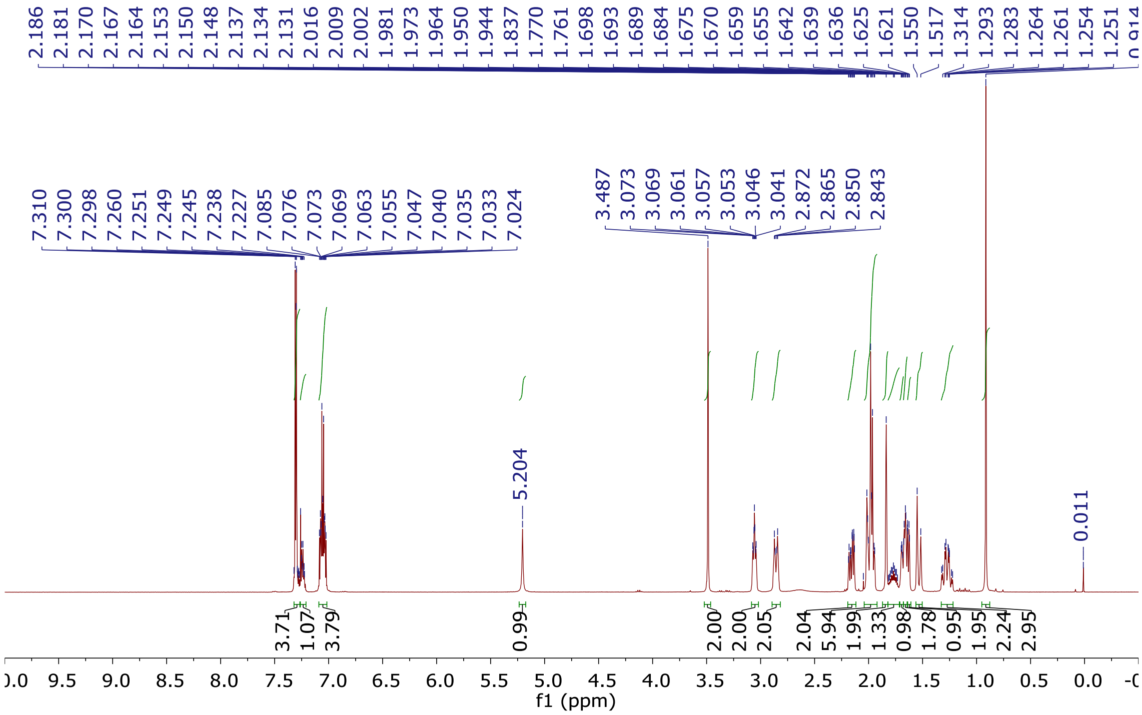

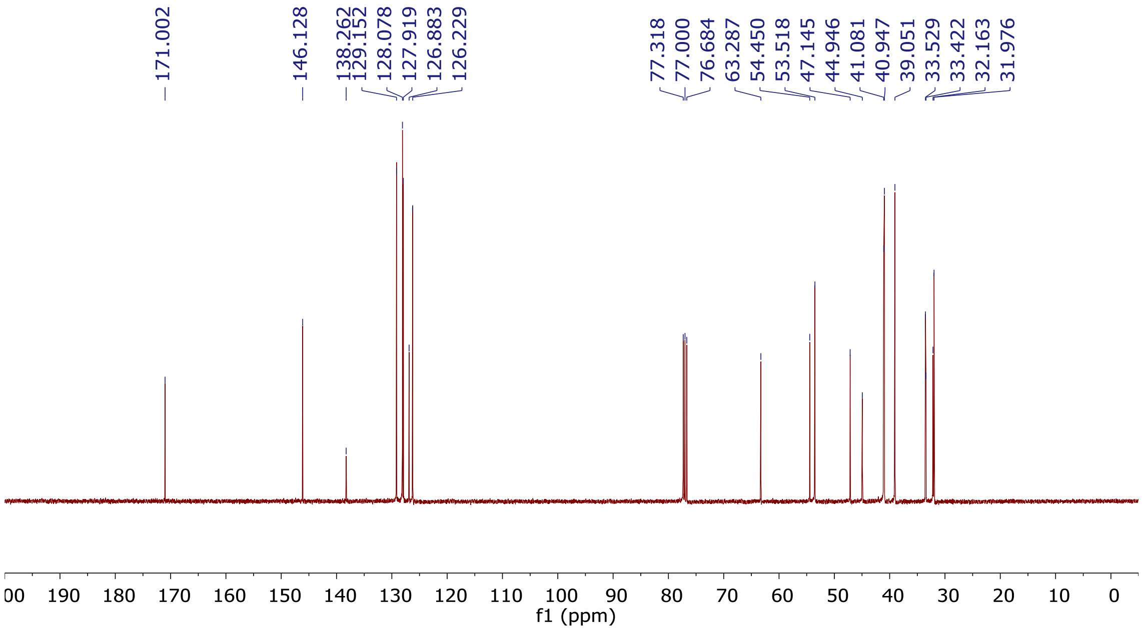

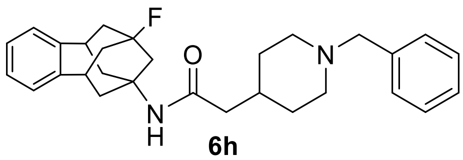
F
O
N
N
H

**6h**

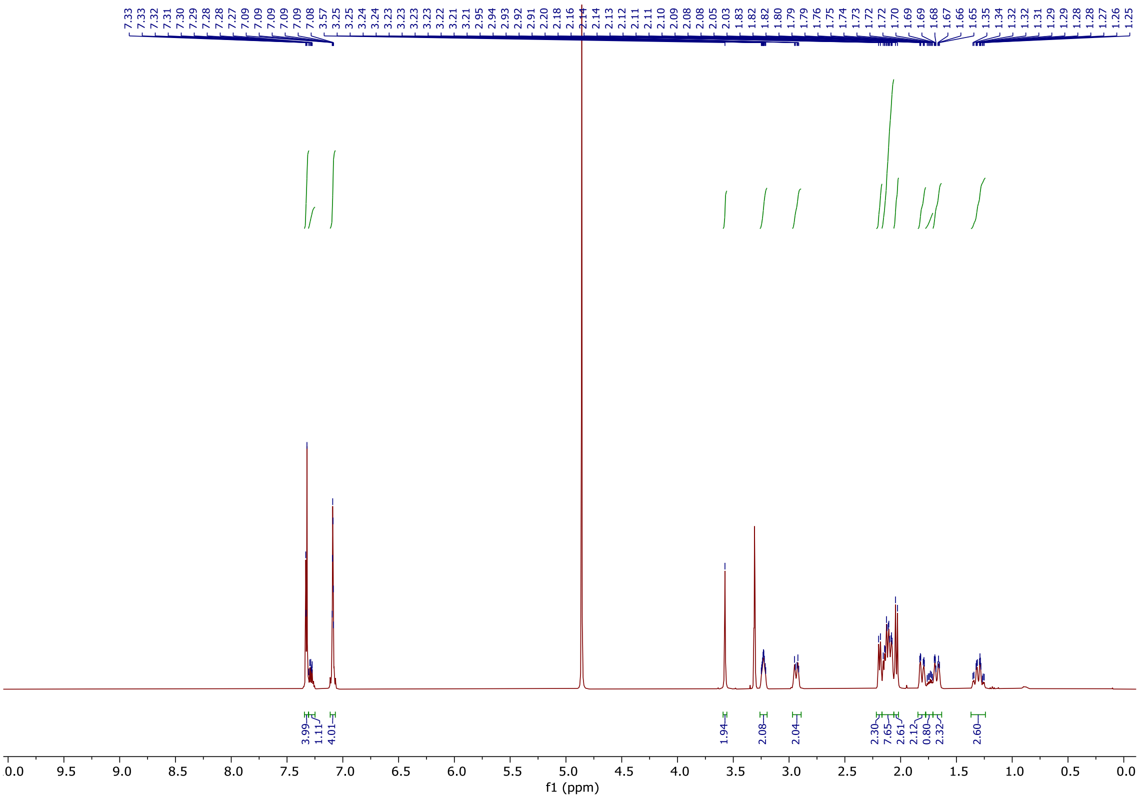
7.33 7.33 7.32 7.31 7.30 7.29 7.28 7.28 7.27 7.09 7.09 7.09 7.09 7.09 7.08 3.57 3.25 3.24 3.23 3.23 3.23 3.23 3.22 3.21 3.21 2.95 2.94 2.93 2.92 2.91 2.20 2.18 2.16 2.14 2.14 2.13 2.12 2.11 2.10 2.09 2.08 2.08 2.05 2.03 1.83 1.82 1.82 1.80 1.79 1.79 1.76 1.75 1.74 1.73 1.72 1.72 1.70 1.69 1.68 1.67 1.66 1.65 1.35 1.34 1.32 1.32 1.31 1.29 1.29 1.28 1.28 1.27 1.26 1.25
3.99 1.11 4.01 1.94 2.08 2.04 2.30 7.65 2.61 2.12 0.80 2.32 2.60
f1 (ppm)

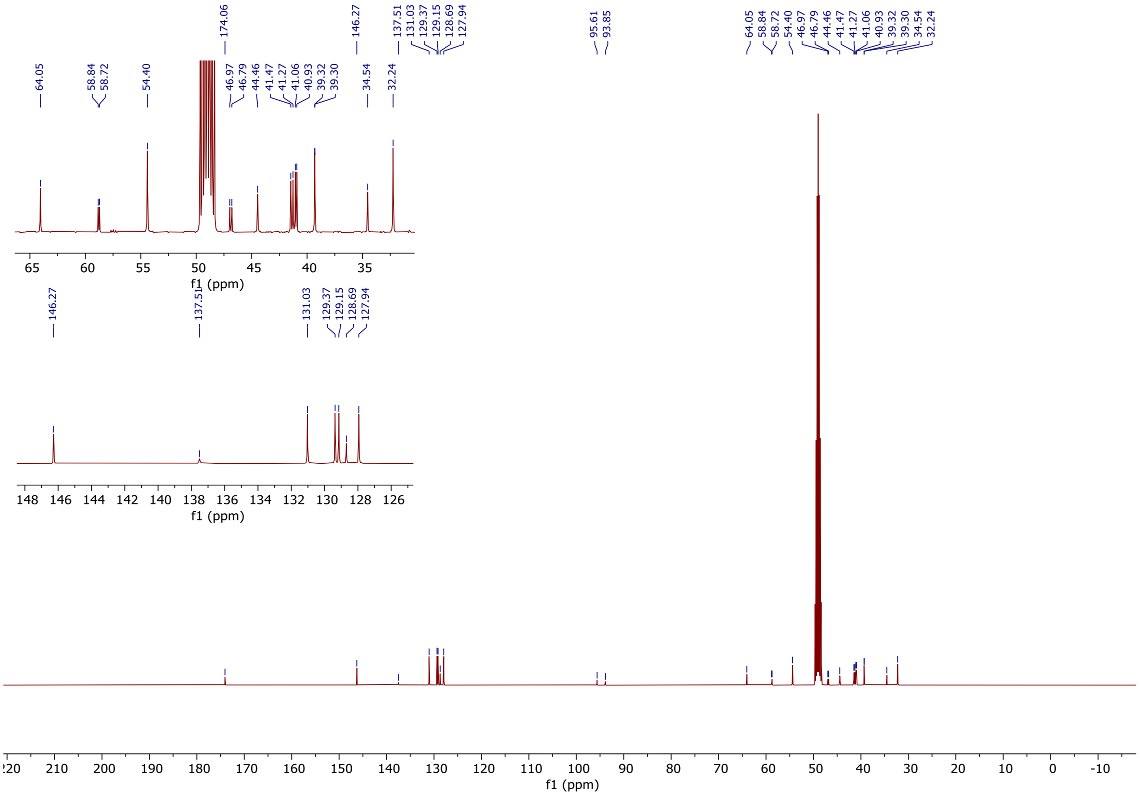
174.06 146.27 137.51 131.03 129.37 129.15 128.69 127.94 95.61 93.85 64.05 58.84 58.72 54.40 46.97 46.79 44.46 41.47 41.27 41.06 40.93 39.32 39.30 34.54 32.24
f1 (ppm)

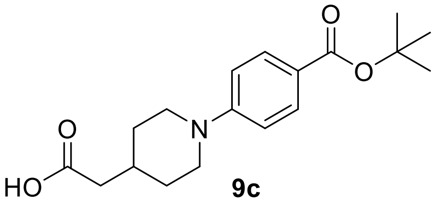

**9c**

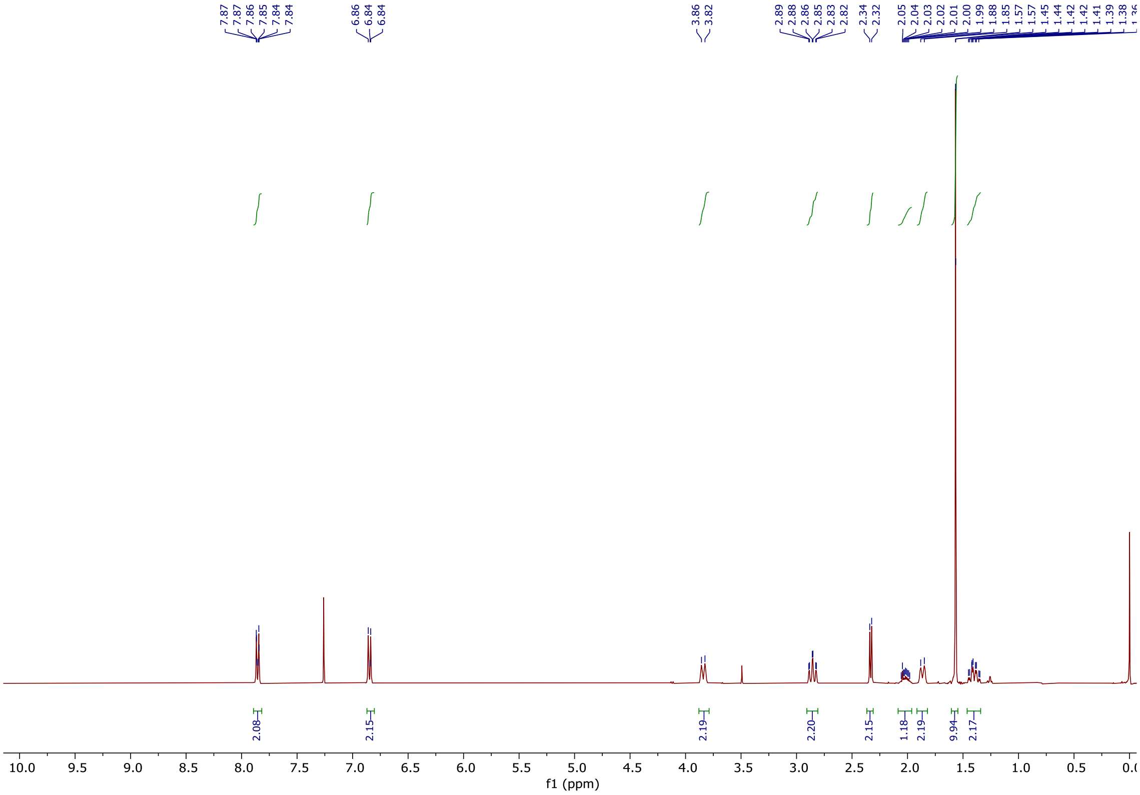
7.87 7.87 7.86 7.85 7.84 7.84
6.86 6.84 6.84
3.86 3.82
2.89 2.88 2.86 2.85 2.83 2.82
2.34 2.32
2.05 2.04 2.03 2.02 2.01 2.00 1.99 1.88 1.85 1.57 1.57 1.45 1.44 1.42 1.42 1.41 1.39 1.38
2.08 2.15 2.19 2.20 2.15 1.18 2.19 9.94 2.17
10.0 9.5 9.0 8.5 8.0 7.5 7.0 6.5 6.0 5.5 5.0 4.5 4.0 3.5 3.0 2.5 2.0 1.5 1.0 0.5
f1 (ppm)

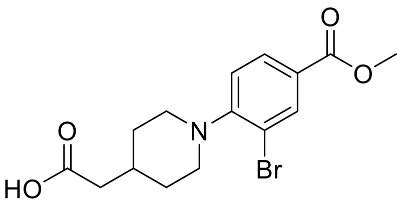
O
O
N
O
HO
Br

**9d**

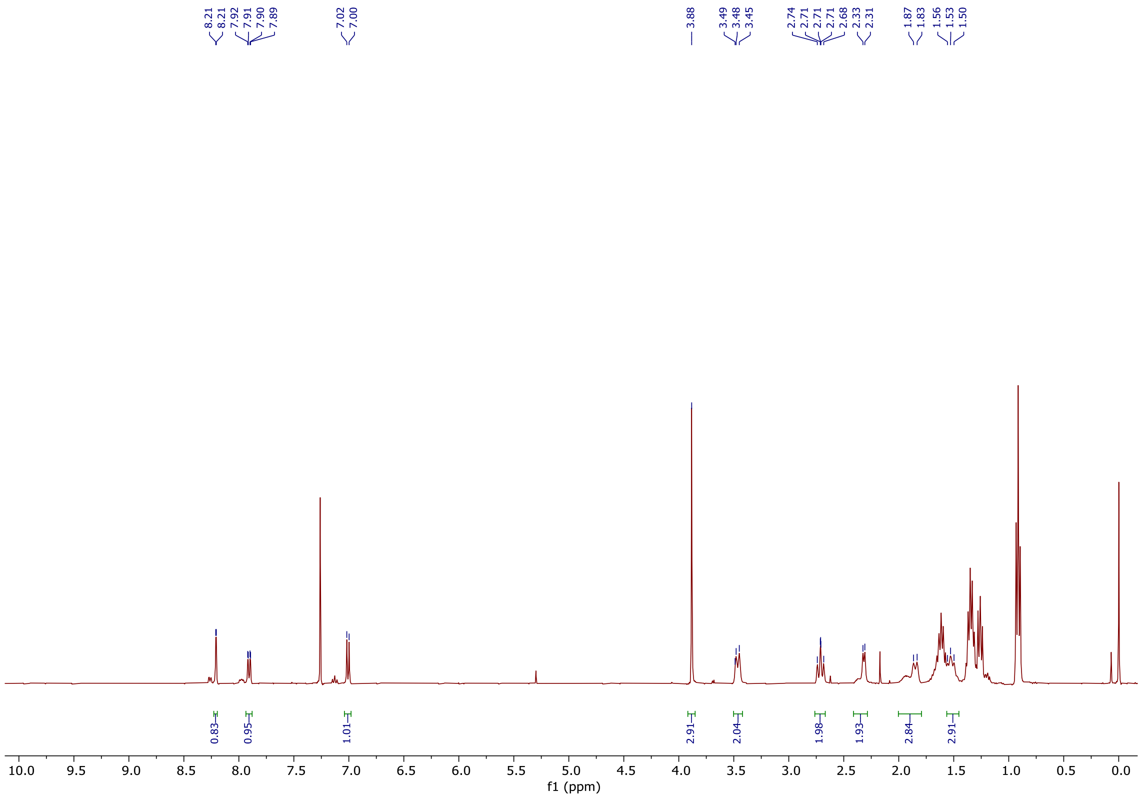
8.21
8.21
7.92
7.91
7.90
7.89
7.02
7.00
3.88
3.49
3.48
3.45
2.74
2.71
2.71
2.71
2.68
2.33
2.31
1.87
1.83
1.56
1.53
1.50
0.83
0.95
1.01
2.91
2.04
1.98
1.93
2.84
2.91
10.0
9.5
9.0
8.5
8.0
7.5
7.0
6.5
6.0
5.5
5.0
4.5
4.0
3.5
3.0
2.5
2.0
1.5
1.0
0.5
0.0
f1 (ppm)

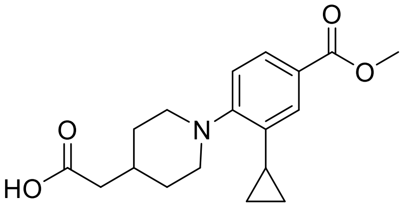
O
O
O
HO
N


**9e**

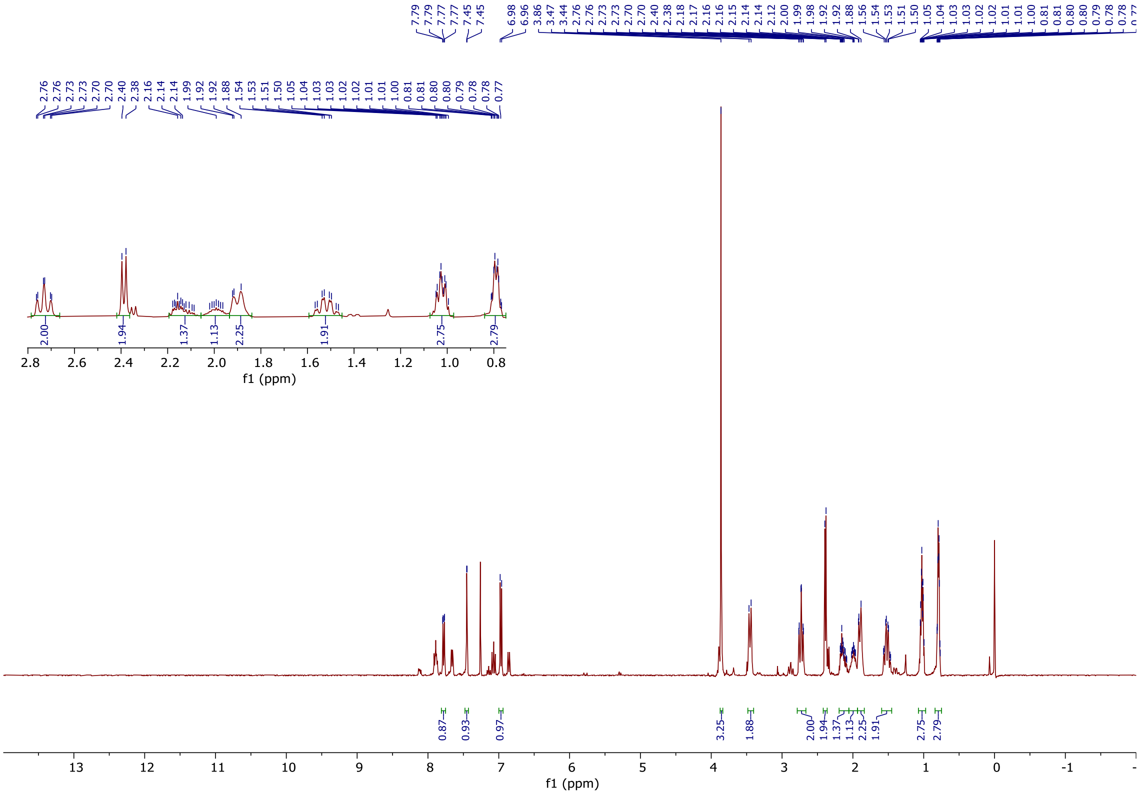
2.00
1.94
1.37
1.13
2.25
1.91
2.75
2.79
2.8 2.6 2.4 2.2 2.0 1.8 1.6 1.4 1.2 1.0 0.8
f1 (ppm)
0.87
0.93
0.97
3.25
1.88
13 12 11 10 9 8 7 6 5 4 3 2 1 0 -1
f1 (ppm)

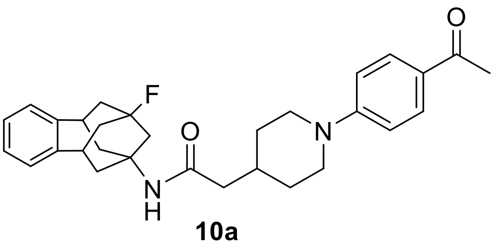
F
O
N
H
O
N

**10a**

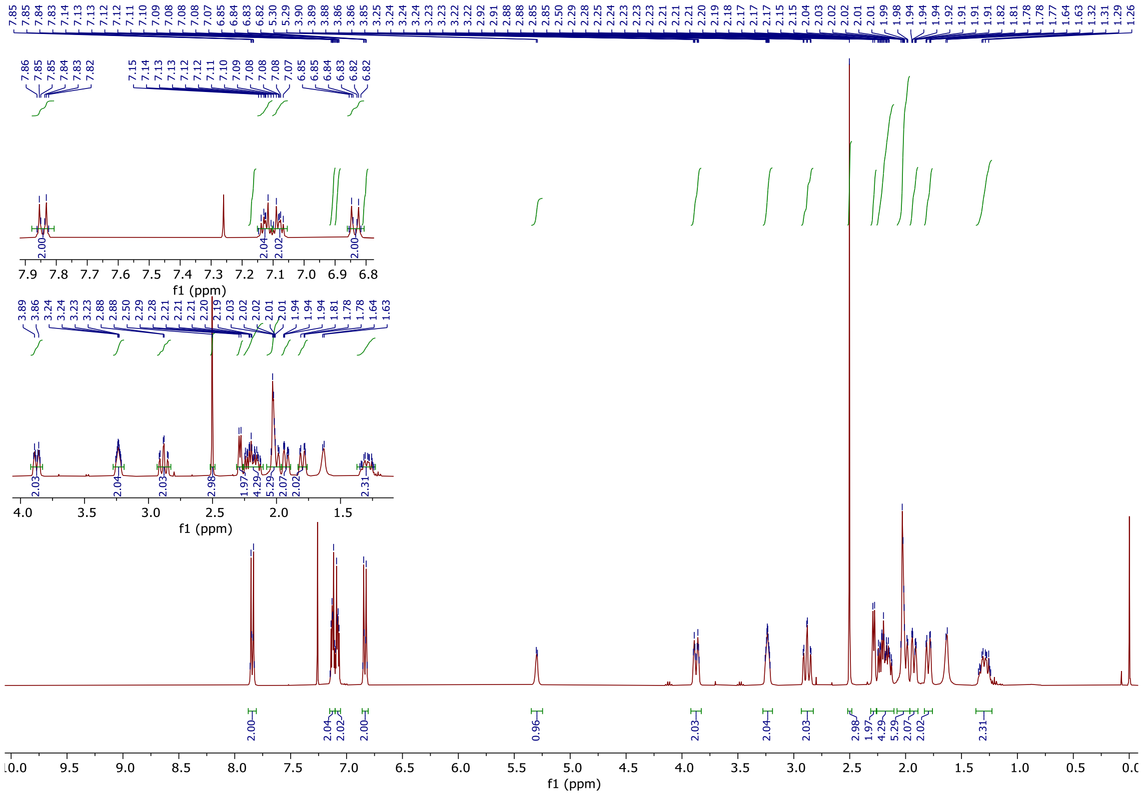
2.00
2.04
2.02
2.00
0.96
2.03
2.04
2.03
2.98
1.97
4.29
5.29
2.07
2.02
2.31
f1 (ppm)

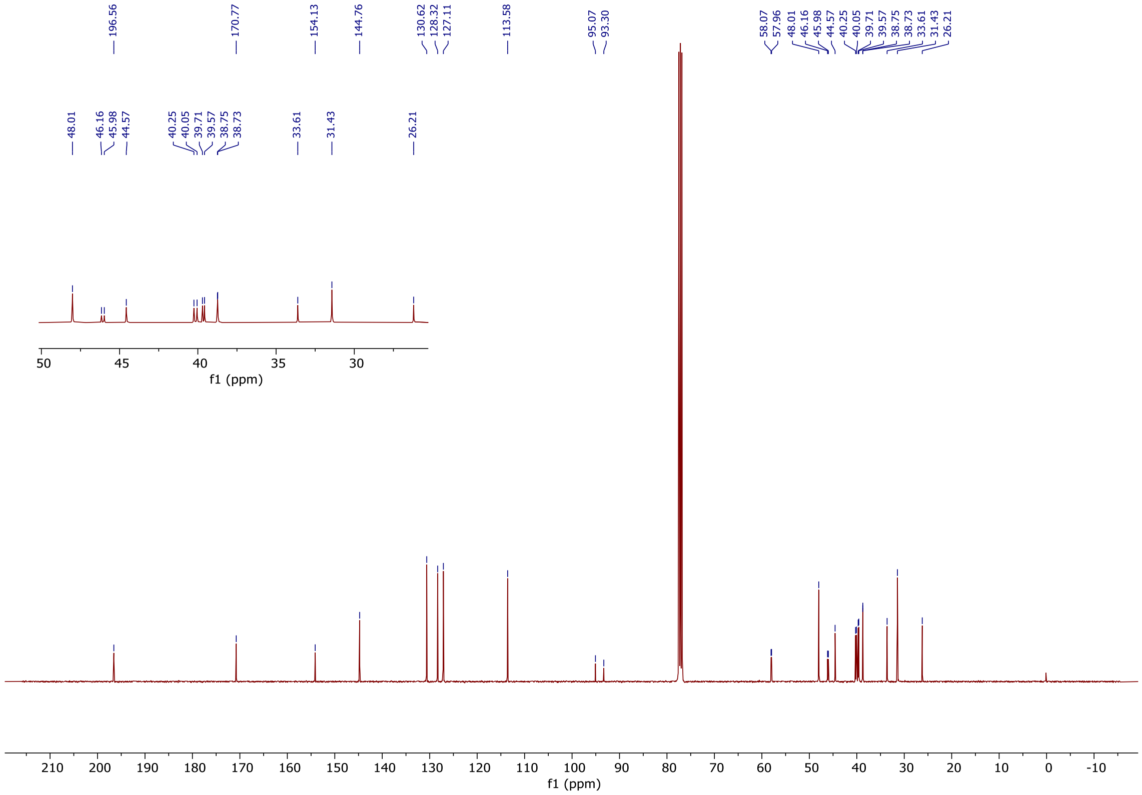
196.56
170.77
154.13
144.76
130.62
128.32
127.11
113.58
95.07
93.30
58.07
57.96
48.01
46.16
45.98
44.57
40.25
40.05
39.71
39.57
38.75
38.73
33.61
31.43
26.21
f1 (ppm)

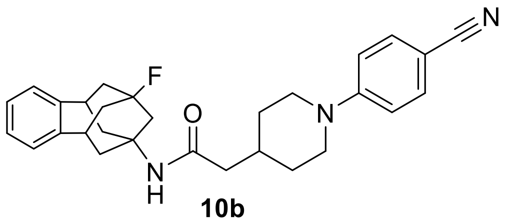

**10b**

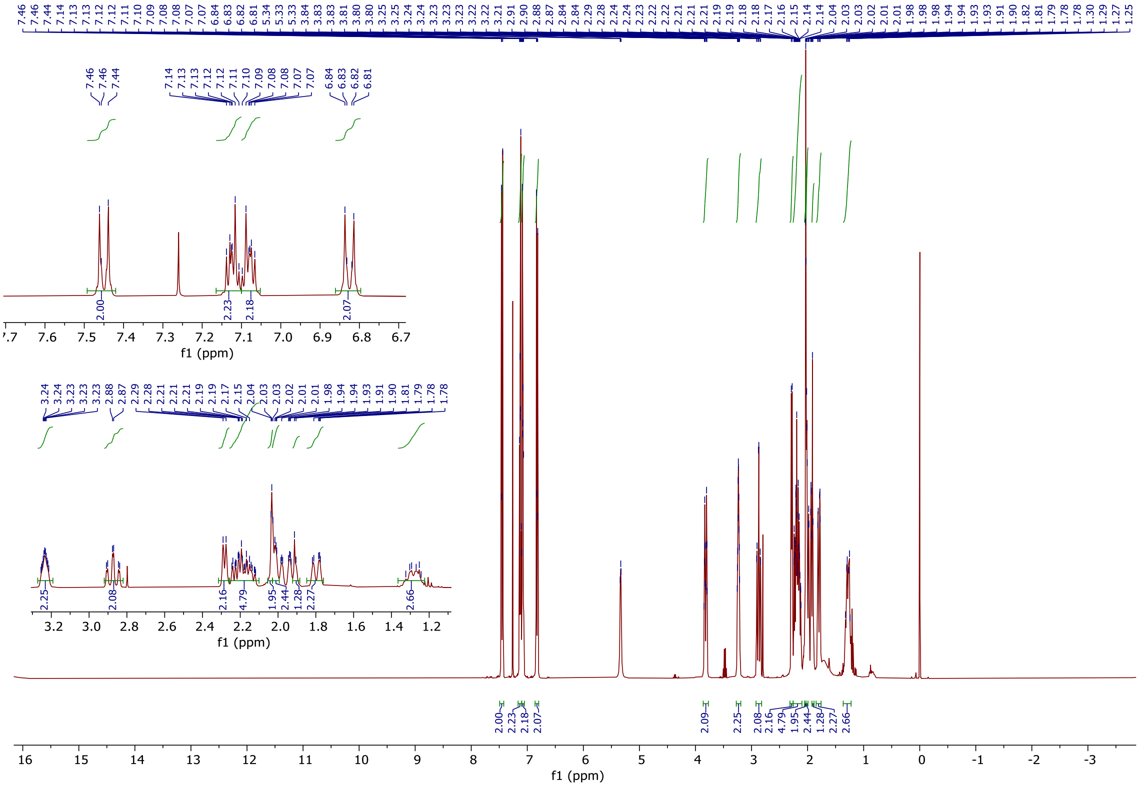
7.46 7.46 7.44 7.14 7.13 7.13 7.12 7.12 7.11 7.10 7.09 7.08 7.08 7.07 7.07 6.84 6.83 6.82 6.81 5.34 5.33 5.33 3.84 3.83 3.81 3.80 3.25 3.25 3.24 3.24 3.23 3.23 3.22 3.22 3.21 2.91 2.90 2.88 2.87 2.84 2.84 2.29 2.28 2.24 2.24 2.23 2.23 2.22 2.22 2.21 2.21 2.19 2.19 2.18 2.18 2.17 2.16 2.15 2.14 2.14 2.04 2.03 2.03 2.02 2.01 2.01 1.98 1.98 1.98 1.94 1.94 1.93 1.93 1.91 1.90 1.82 1.81 1.79 1.78 1.78 1.30 1.29 1.27 1.25
2.00 2.23 2.18 2.07 2.09 2.25 2.08 2.16 4.79 1.95 2.44 1.28 2.27 2.66
f1 (ppm)

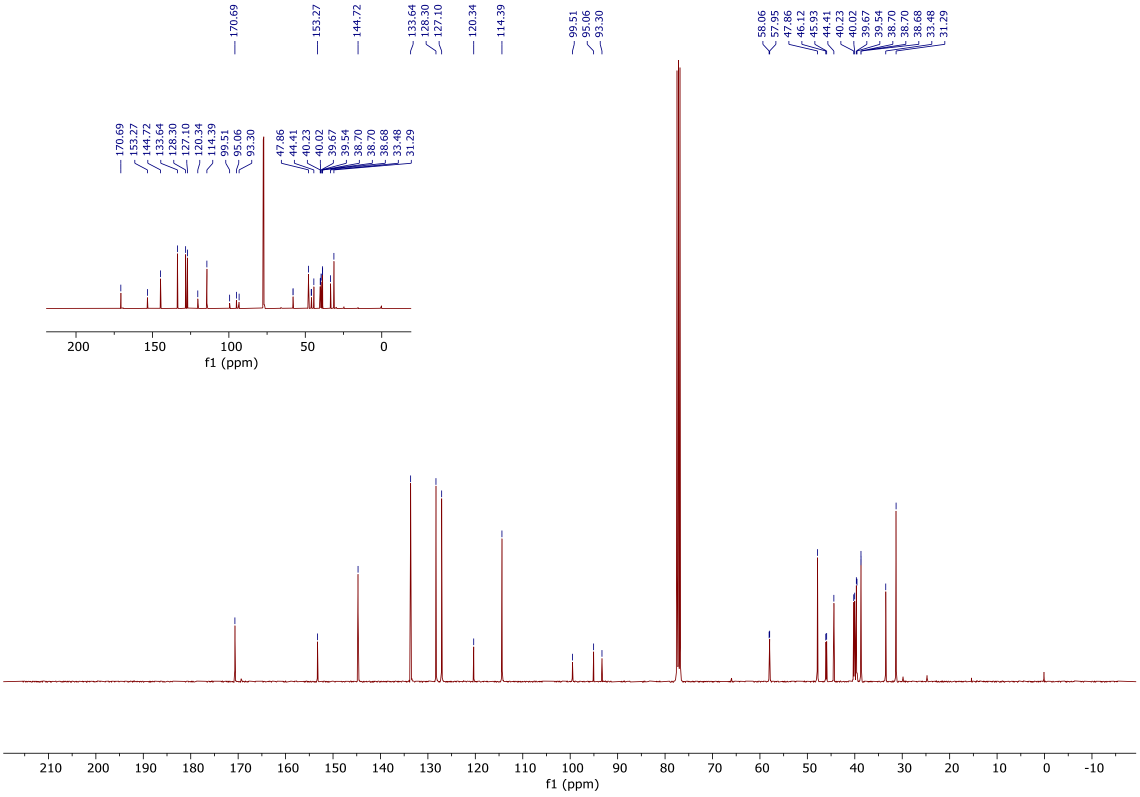
170.69 153.27 144.72 133.64 128.30 127.10 120.34 114.39 99.51 95.06 93.30 58.06 57.95 47.86 46.12 45.93 44.41 40.23 40.02 39.67 39.54 38.70 38.70 38.68 33.48 31.29
f1 (ppm)

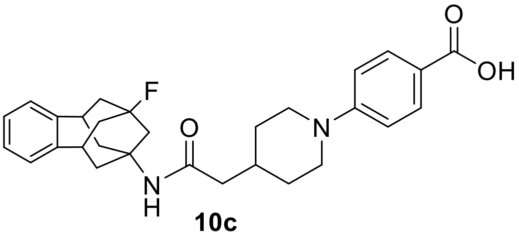
F
O
OH
N
N
H

**10c**

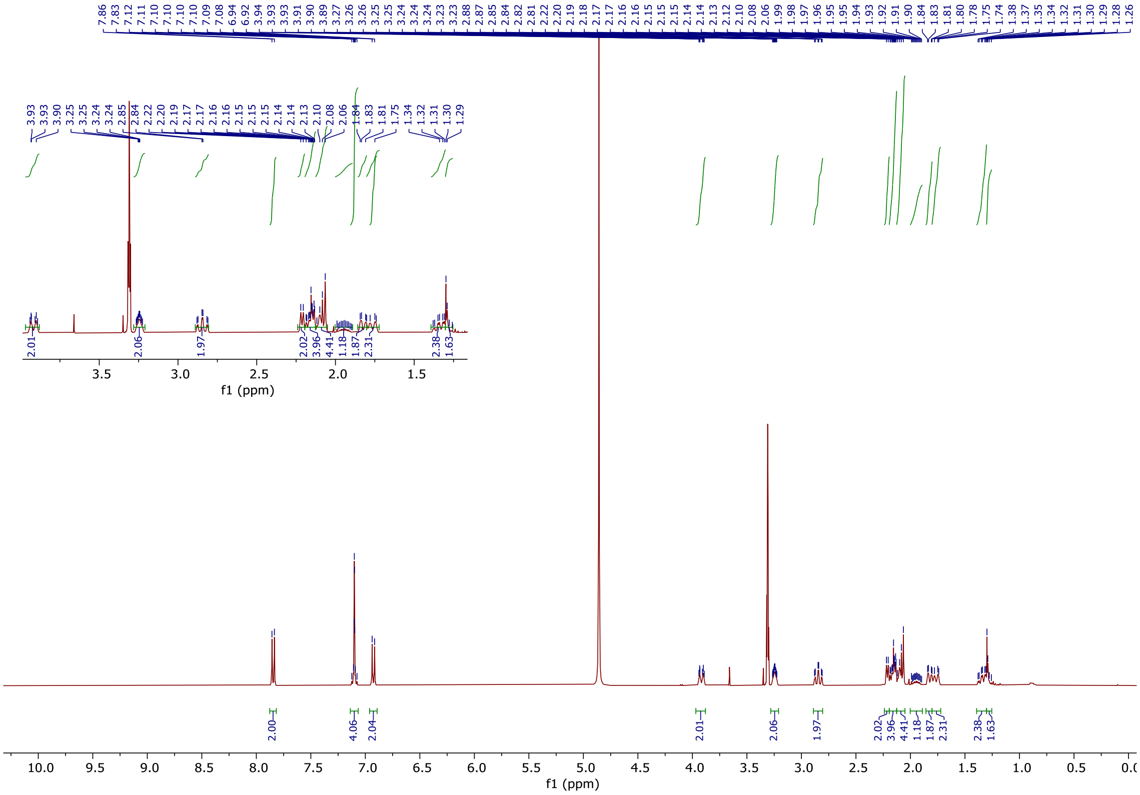
7.86 7.83 7.12 7.11 7.10 7.10 7.10 7.10 7.09 7.08 6.94 6.92 3.94 3.93 3.93 3.91 3.90 3.89 3.27 3.26 3.26 3.25 3.25 3.24 3.24 3.24 3.23 3.23 2.88 2.87 2.85 2.84 2.82 2.81 2.22 2.20 2.19 2.18 2.17 2.17 2.16 2.16 2.15 2.15 2.15 2.14 2.14 2.13 2.12 2.10 2.08 2.06 1.99 1.98 1.97 1.96 1.95 1.95 1.94 1.93 1.92 1.91 1.90 1.84 1.83 1.81 1.80 1.78 1.75 1.74 1.38 1.37 1.35 1.34 1.32 1.31 1.30 1.29 1.28 1.26
2.00 4.06 2.04 2.01 2.06 1.97 2.02 3.96 4.41 1.18 1.87 2.31 2.38 1.63
f1 (ppm)

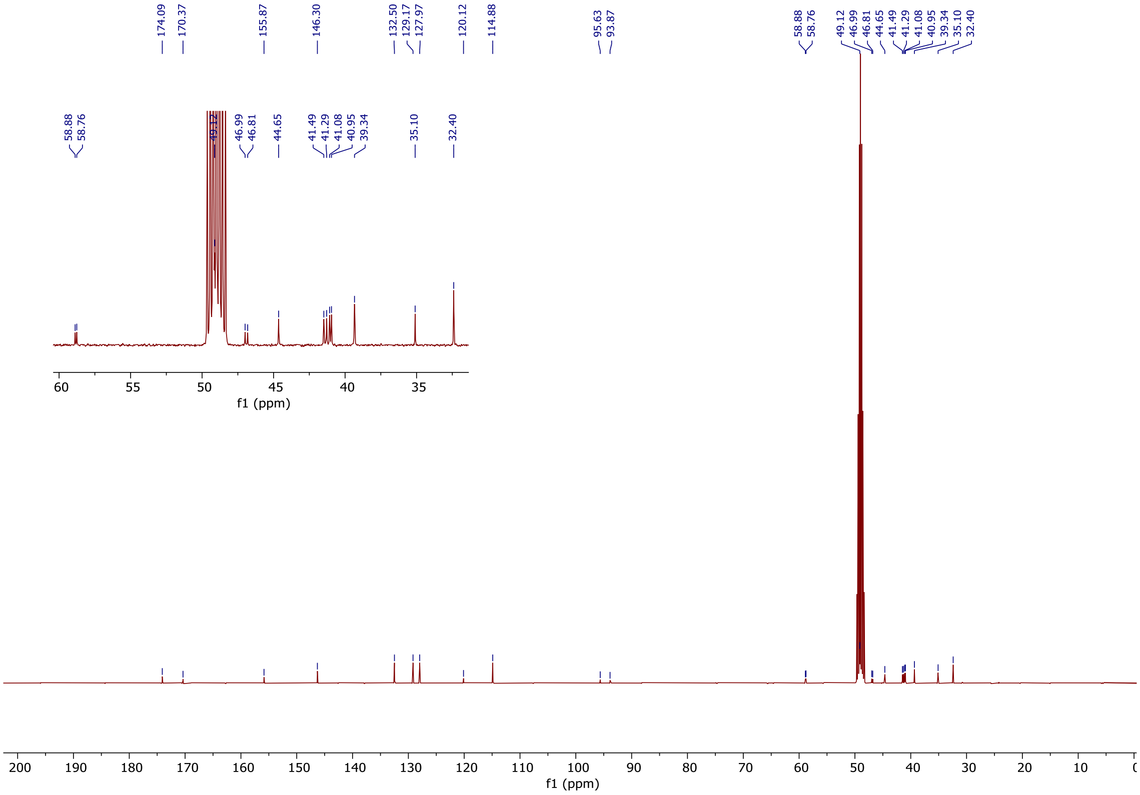
174.09 170.37 155.87 146.30 132.50 129.17 127.97 120.12 114.88 95.63 93.87 58.88 58.76 49.12 46.99 46.81 44.65 41.49 41.29 41.08 40.95 39.34 35.10 32.40
f1 (ppm)

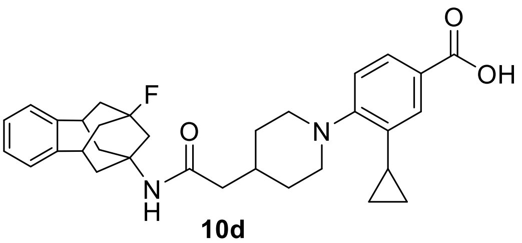

**10d**

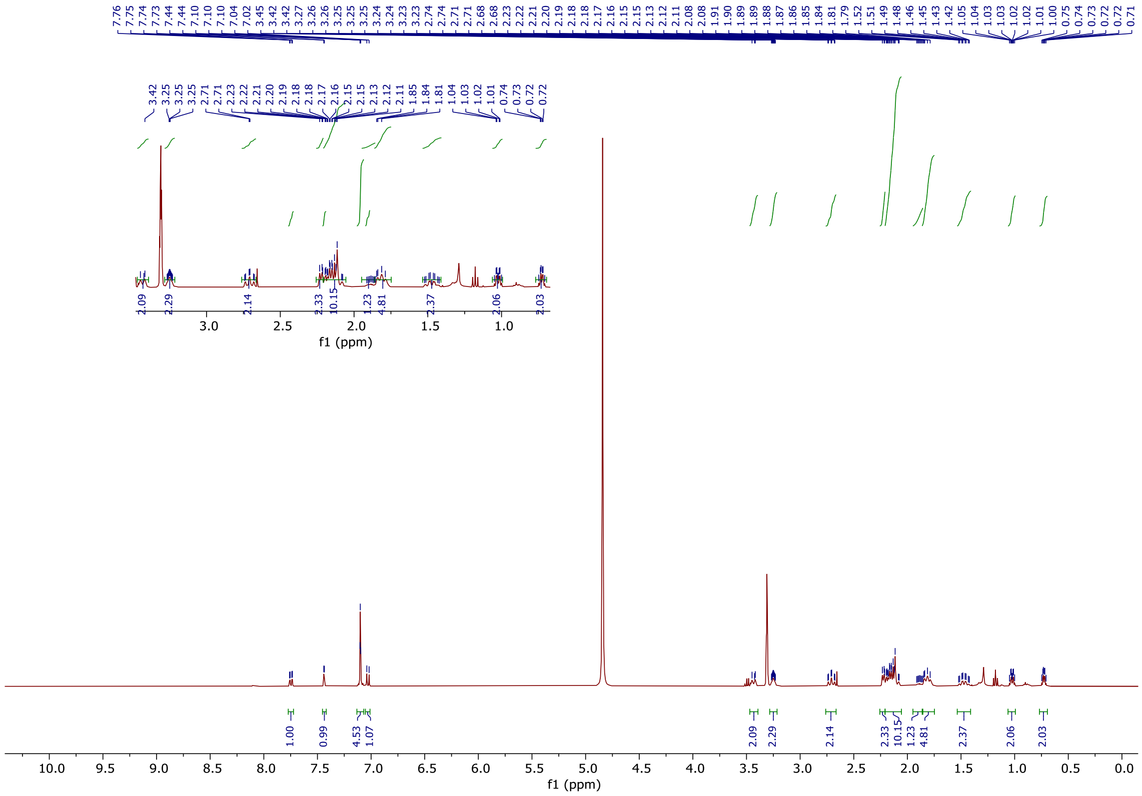

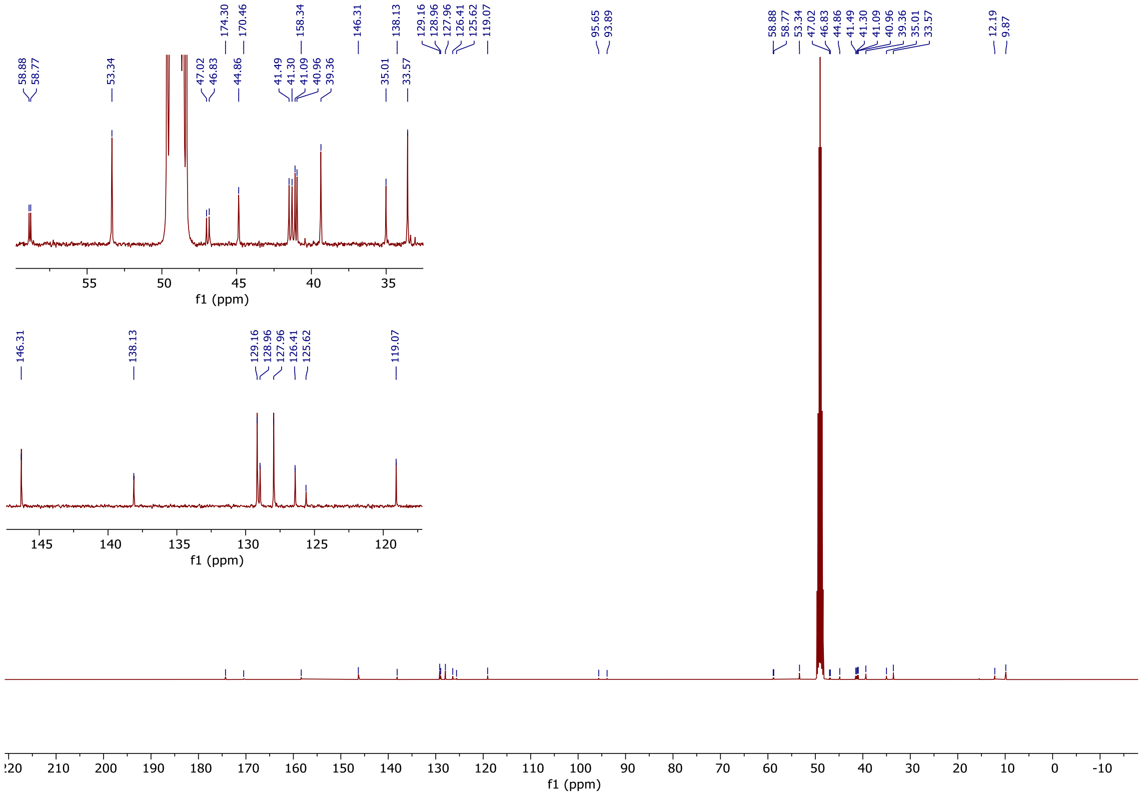

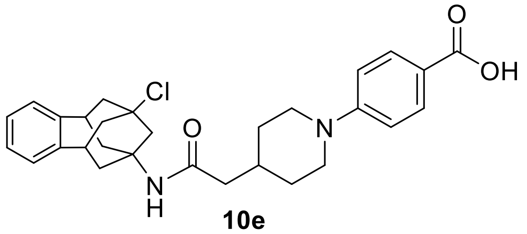

**10e**

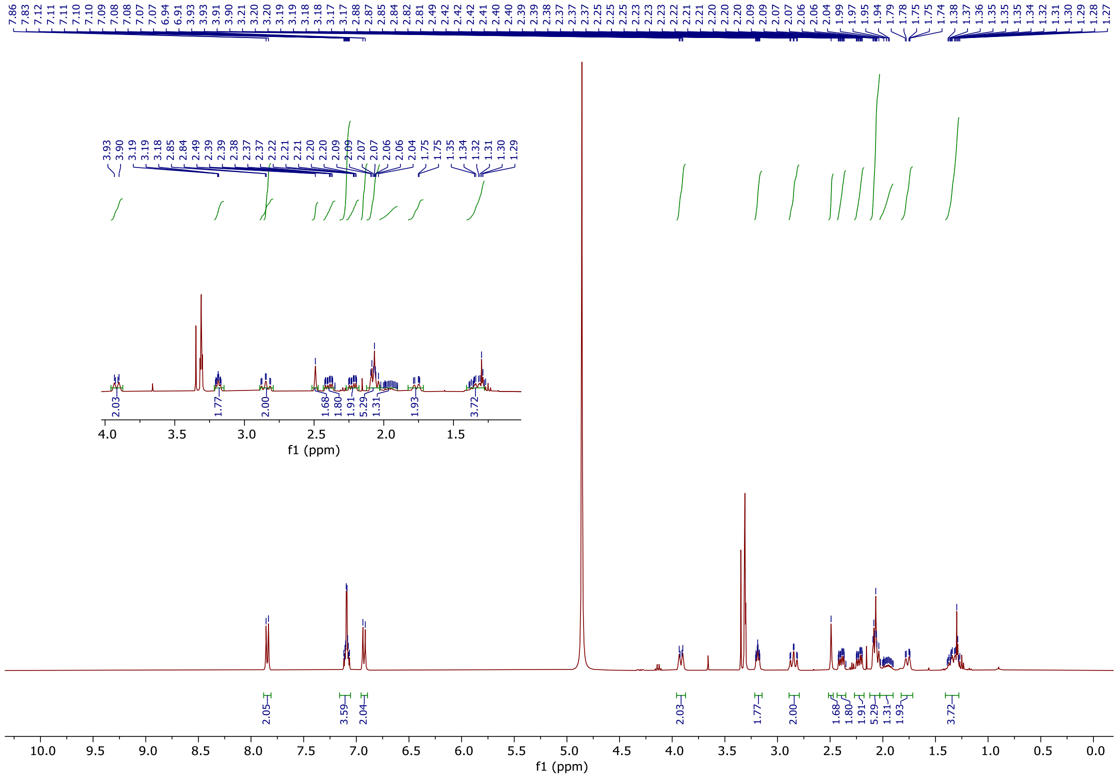

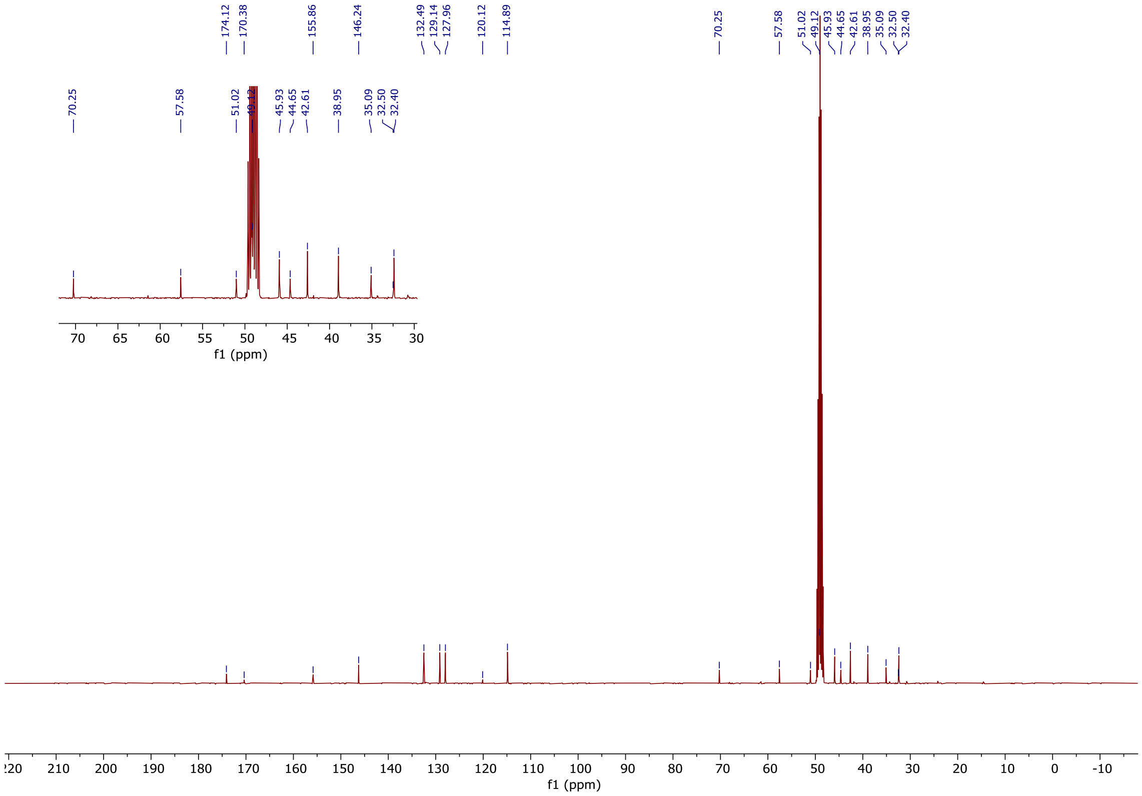

Supplement: Supplementary file 1 [file pharmaceuticals-14-01323-s001.zip › pharmaceuticals-1505615-supplementary.pdf]
